# Methodological characteristics of evaluative studies of drug prevention programs in Brazil: scoping review^*^

**DOI:** 10.1590/1518-8345.7709.4578

**Published:** 2025-07-11

**Authors:** Daniela Ribeiro Schneider, Charlene Fernanda Thurow, Tallita Franzoloso, Leila Gracieli da Silva, Guilherme Gomes Silva, Elaine Lucas dos Santos, Liz Paola Domingues, Leila Pimentel dos Anjos, Fernanda Machado Lopes, Ana Regina Noto

**Affiliations:** 1 Universidade Federal de Santa Catarina, Departamento de Psicologia, Florianópolis, SC, Brazil.; 2 Scholarship holder at the Conselho Nacional de Desenvolvimento Científico e Tecnológico (CNPq), Brazil.; 3 Scholarship holder at the Coordenação de Aperfeiçoamento de Pessoal de Nível Superior (CAPES), Brazil.; 4 Universidade Estadual do Norte do Paraná, Departamento de Enfermagem, Jacarezinho, PR, Brazil.; 5 Universidade Federal de São Paulo, Departamento de Psicobiologia, São Paulo, SP, Brazil.

**Keywords:** Programs, Prevention, Review, Drug Abuse, Brazil, Public Policy

## Abstract

to map studies that have developed evaluation processes of prevention programs for the use of alcohol and other drugs in Brazil, analyzing their methodological characteristics.

a scoping review guided by the Joanna Briggs Institute Manual and the Preferred Reporting Items for Systematic reviews and Meta-Analyses extension for Scoping Reviews. The searches were carried out in eight databases and a theses and dissertations database, screened with double-blind in Rayyan®, analyzed with a focus on the methodology of the evaluative studies and through qualitative synthesis.

of the 56 publications included, the majority used an observational design, generally related to process evaluations (n = 26), followed by experimental (n = 23) and quasi-experimental (n = 8) analyses of results in terms of effectiveness and efficacy, respectively. Of the eleven actions analyzed, eight are universal prevention programs, one is selective prevention and two are a preventive strategy.

most of the programs evaluated are universal, aimed at adolescents, carried out in schools and focused on various drugs. It is recommended to focus on new groups, such as Indigenous populations and other risk conditions, and to develop selective programs in future government initiatives to prevent drug use in Brazil.

## Highlights

**(1)** Nine drug prevention programs with evaluation processes were found in Brazil. **(2)** Of the nine programs, eight are universal prevention programs and one is selective prevention. **(3)** Some programs have consistent evidence to support public policies. **(4)** PROERD has not demonstrated its effectiveness in prevention and needs to be remodeled. **(5)** There is a need to broaden the scope of preventive programs with evidence in Brazil.

## Introduction

Since the 1970s, prevention has gradually consolidated as a science based on consistent theoretical foundations and methodological rigor. The construction of evidence has become essential to ensure the effectiveness of preventive actions and the achievement of expected results, with the aim of formulating public policies that prioritize quality, cost-effectiveness and health promotion. In this context, it is essential that countries adopt this scientific perspective in order to guarantee the quality of their policies^(1)^.

In Brazil, although many preventive actions are carried out, evaluation to prove the effectiveness of these initiatives is not so common. This fact, coupled with the one-off nature of the interventions, makes it difficult for the field to promote its evidence effectively and shows that the process of consolidating prevention science in the country is still at an emerging stage^(1)^.

One of the first review studies on drug use prevention programs in Brazil gathered research published between 1991 and 2001 and concluded that prevention actions in the 1990s were driven by the prevention model adopted to face the challenges of Sexually Transmitted Diseases^(2)^. These actions were criticized for their reductionist nature, focusing only on the disease and following the logic of the “war on drugs” that predominated in this field.

In this context, they began to look more closely at the relationship between personal and contextual vulnerabilities, and the first projects based on the logic of harm reduction appeared. Scientific production was still very much focused on the pharmacological aspects of the drug itself and the treatment of addiction. Most of the preventive projects analyzed were aimed at schools, influenced by specialists who advocated the school environment as a privileged place to develop this type of intervention. Pilot projects were developed in public schools monitored by the Ministry of Health, but they still used traditional methodologies, based on lectures and passing on information, mainly carried out by non-school agents, such as doctors and police officers. In this review, carried out until 2001, there were no studies on the efficacy and effectiveness of the programs analyzed^(2)^.

A subsequent systematic review of articles published up to 2012 analyzed publications evaluating mental health programs in Brazil, including behavioral problems, violence, sexual abuse, alcohol and drug abuse and existential emptiness^(3)^. A predominance of pre-experimental and quasi-experimental studies were found, with no follow-up evaluations and small samples. Most of the evaluations were process or needs assessments, or pilot tests of programs, with few studies evaluating efficacy, effectiveness and dissemination^(3)^. So far, there has been some development of prevention science in Brazil, but it is still in its infancy.

Another more recent study, published in 2020, carried out with 1151 school leaders, on preventive actions and programs carried out in Brazilian public and private schools, found that the programs are still implemented without regularity and with short duration, aimed mainly at students, using different theoretical models and promoted mainly by the Military Police^(4)^. Thus, more than three decades of research and reviews demonstrate the gradual evolution of Brazilian prevention science, which still faces many difficulties and needs more incentives for its development.

The year 2013 was a milestone for the strengthening of Brazilian prevention science, due to an important initiative by the Federal Government that boosted the main research and implementation of evidence-based programs in the country, contributing to changing the landscape of prevention science in the country. The action was carried out in partnership between the Ministry of Health’s Coordination of Mental Health, Alcohol and Other Drugs, with the United Nations Office on Drugs and Crime (UNODC), the Ministry of Justice’s National Drug Policy Secretariat (SENAD) and the Oswaldo Cruz Foundation (FIOCRUZ). Three programs then underwent cultural adaptation processes in Brazil: ELOS: Building Collectives (ELOS) (from the original Good Behavior Game), #tamojunto (Unplugged) and Strengthening Families Programme, seeking to cover preventive actions throughout the life cycle. The first is aimed at children, the second at adolescents and the third at strengthening parental relationships within families^(5)^. Independent initiatives have also gained ground, such as the Brazilian adaptation of the Australian program The School Health and Alcohol Harm Reduction Project (SHAHRP)^(6)^. This experience has generated a much more consistent and robust process of evidence production, strengthening the relationship between those who formulate and implement preventive policies and the research institutes that evaluate these initiatives^(5)^. On the other hand, this experience has been challenging, as importing structured programs can bring difficulties related to cultural adaptations to the local reality^(7-8)^. This can result in a loss of evidence due to changes in outcomes, which can become unfavorable, as well as compromising fidelity to the core aspects of the original program and facing the challenges inherent in large-scale implementation^(5)^. Therefore, strengthening evaluation processes is a necessity and a major challenge.

A better understanding of the progress made in implementing preventive actions for drug use and health promotion in Brazil is necessary to support the implementation of public policies, such as the National System for the Prevention of the Use of Alcohol and Other Drugs, linked to SENAD. Therefore, a detailed study of the methodological characteristics of strategies and programs for alcohol and other drug use that have already undergone evaluation processes could help provide a menu of preventive actions, which is necessary for the implementation of preventive policies. In this way, Brazil could begin to consolidate the development of a platform for the certification of strategies and programs, based on clear scientific standards, as is already the case in other countries. Likewise, this analysis helps Brazilian prevention science, which is still in the process of maturing, to look at its history and think about its advances and challenges.

Considering the problem described above, this current scoping review aims to map studies that have developed evaluative processes of prevention programs for the use of alcohol and other drugs in Brazil, analyzing their methodological characteristics, published between 2011 and 2023. It seeks to contribute to the development of a menu of evidence-based preventive initiatives available in Brazil, which can subsidize preventive public policies on drugs and assist in decision-making by managers, professionals and researchers.

## Method

### Type of study

The scoping review was the method chosen to respond to the objective of this research because it makes it possible to map the existing literature in a given field and systematize the conceptual and methodological characteristics of research^(9)^. This review was guided by the Joanna Briggs Institute Handbook and the guidelines of the Preferred Reporting Items for Systematic reviews and Meta-Analyses extension for Scoping Reviews Checklist (PRISMA-ScR)^(10)^ and carried out the following steps: 1) drawing up the protocol (registered with PROSPERO under number 2022 CRD42022330854, access link: https://www.crd.york.ac.uk/prospero/display_record.php?ID=CRD42022330854), specifying the research question, defining the eligibility criteria, databases and search strategies; 2) identifying the relevant studies by applying the search strategies in the selected databases; 3) selecting the studies based on the eligibility criteria; 4) extracting and analyzing the data; 5) systematizing and summarizing the data and reporting the results. The protocol (available on request from the corresponding author) was developed by the research team, made up of specialists in the science of prevention in psychology, professionals with experience in systematic reviews in the health area and a librarian.

The research question guiding this review was: What are the methodological characteristics of studies investigating drug use prevention programs evaluated in Brazil? The components of the question followed the acronym PCC (item 4 of the PRISMA ScR), in which “participants” (P) were complete empirical studies conducted with human beings; “concept” (C) were drug prevention programs that underwent some evaluation process (of temporal effect, efficacy, effectiveness or implementation process); and “context” (C) was the Brazilian context.

### Selection criteria and scenario

Included were experimental and quasi-experimental studies, methodological studies, analytical and descriptive observational studies, qualitative approaches, theses and dissertations whose central theme was the application of a drug prevention program evaluated in Brazil. Studies published between January 1, 2010 and December 31, 2023 were included, with no language restrictions. Studies with a non-human population were excluded (wrong population); editorials, letters, errata, books, book chapters and papers presented at congresses (wrong type of publication); theoretical studies and reviews of any kind - integrative, narrative, systematic, meta-analysis (wrong study design); studies that investigated prevention programs on a focus other than drugs, or that did not present program evaluation results, or that were not conducted in Brazil (wrong theme).

The systematic search was carried out in two stages: the first, on August 31, 2021, when this review began, and an update on January 31, 2024. The databases consulted were: Medical Literature Analysis and Retrieval System Online (Medline) via Publisher MEDLINE (PubMed); Excerpta Medica Database (EMBASE); Psychological Information Database (PsycINFO); Web of Science, Scientific Electronic Library Online (SciELO); *Literatura Latino-Americana e do Caribe em Ciências da Saúde* (LILACS) and Índice de Literatura Técnico-Científica em Psicologia (IndexPsi), both part of the *Biblioteca Virtual em Saúde* - *Psicologia Brasil* (BVS-Psi Brasil); the database provider Elton B. Stephens Company (EBSCO); and *Banco de Saúde*. Stephens Company (EBSCO); and Thesis and Dissertation Database of the Coordination for the Improvement of Higher Education Personnel (CAPES). The databases were chosen on the basis of their global scope with a multidisciplinary focus (e.g. Web of Science), with a wide range of articles in the health area (e.g. Medline via PubMed) and psychology (e.g. PsycINFO), and the fact that they index articles published in journals from Latin America and the Caribbean (e.g. LILACS and SciELO), since the focus was on studies carried out in Brazil.

The indexes used in the searches were “Program”, “Prevention”, “Evaluation”, “Drug”, and “Brazil”, and the complete search strategies, including the descriptors and Boolean operators used, can be accessed at https://www.crd.york.ac.uk/PROSPEROFILES/330854_STRATEGY_20220506.pdf. In addition to the databases, experts on the subject of prevention science in Brazil were consulted in order to reduce selection bias and broaden the possibilities of accessing the largest number of studies on the subject. We did not systematically check all the references of the articles included, as a preliminary analysis of these did not reveal any new articles within our search period.

The references obtained from the database searches were imported using Endnote, where duplicates were excluded. Subsequently, the references were imported into Rayyan®^(11)^ to select the relevant studies, applying the eligibility criteria in a double-blind format.

### Data collection, processing and analysis

The first ten abstracts were assessed in an online meeting with the whole team, using Rayyan®, to standardize the list of exclusion reasons. Afterwards, eight independent judges screened the double-blind studies by reading the other titles and abstracts. Discrepancies were resolved in a meeting with at least 50% of the team until a consensus was reached. After screening, the same procedure was used by the eight judges to calibrate the reading and extract the full texts.

To extract the data, the research team prepared a matrix spreadsheet in Excel software, based on the PRISMA-ScR guidelines. The spreadsheet was organized into five major sections to extract data: 1) General characterization of the studies (year of publication and design); 2) Method of the studies, such as specification of the target audience (number and gender), use of assessment instruments before and after the intervention, procedures (e.g. follow-up) and outcomes assessed (type of drug, pattern of consumption); 3) Data on the drug prevention program, such as the name of the Program, the context (e.g. school, health), the scope (e.g. national, regional) and the procedures (number of sessions and duration, type of drug). national, regional) and procedures (number of sessions and duration, type of drug the program targets, type - workshop, lecture, experience - and prevention category); 4) Data on the procedures for evaluating the program’s implementation process, i.e. whether preparatory studies (such as needs assessment and pilot studies), fidelity, satisfaction, feasibility and sustainability studies were carried out; and 5) Data on the evaluation of the program’s results, i.e. outcomes that improved, worsened and were maintained, and data on efficacy, effectiveness and cost-benefit.

Considering that this is a scoping review, which seeks to summarize the characteristics of the mapped studies of drug prevention programs carried out in Brazil, tools for analyzing methodological quality, risk of bias and quality of evidence were not applied, as these items are applicable to systematic reviews aimed at evaluating the effects of interventions.

## Results

**Figure 1 -:**
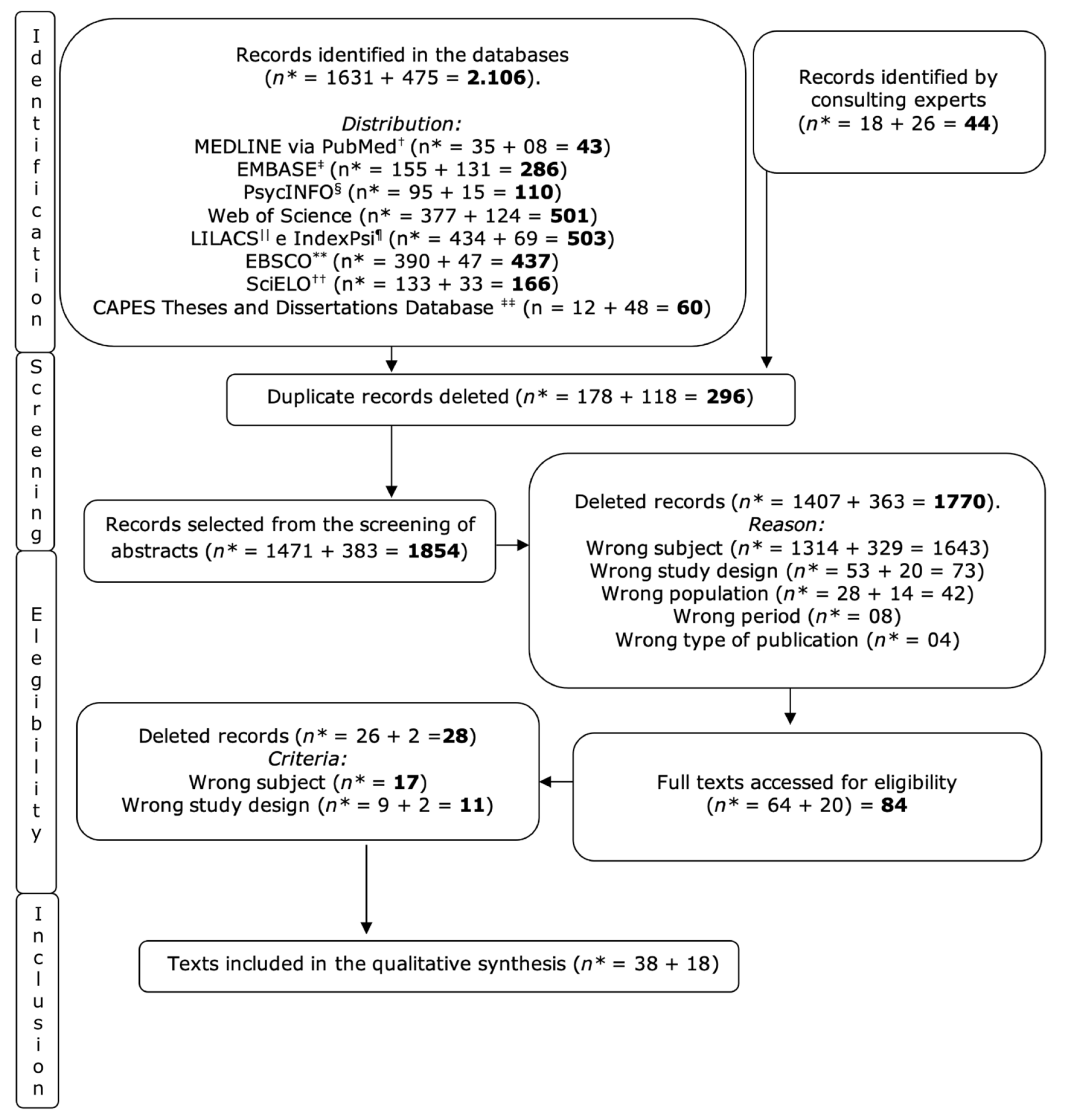
Flowchart of the search, screening, eligibility and inclusion stages

Based on the searches in the selected databases, 2106 texts were identified, of which 1631 in the searches carried out on 31/08/2021 (texts from 1/01/2010 to 31/08/2021) and 475 in the searches carried out on 31/01/2024 (texts from 01/09/2021 to 31/12/2023). We also added 44 texts from consultations with specialists (using the same descriptors), in September 2021 and January 2024, for a total of 2150 publications found. No material was included from reading the references of the selected articles. After excluding 296 duplicate studies (178 in the first and 118 in the second search), 1854 went on to the screening stage. Once the inclusion criteria had been applied after reading the abstracts (reasons for exclusion detailed in Figure 1), 84 studies went on to the full text reading stage. Of these, 28 texts were eliminated by the exclusion criteria, leaving 56 publications included for qualitative analysis and synthesis. Figure 1 shows the flowchart detailing this process.

*n = Number of studies; ^†^Medline via PubMed = Medical Literature Analysis and Retrieval System Online via Publisher Medline; ^‡^EMBASE = Excerpta Medica Database; ^§^PsycINFO = Psychological Information Database; ^||^LILACS = *Literatura Latino-Americana e do Caribe em Ciências da Saúde*; ^¶^IndexPsi = Índice de Literatura Técnico-Científica em Psicologia; **EBSCO = Elton B. Stephens Company; ^††^SciELO = Scientific Electronic Library Online; ^‡‡^CAPES = *Coordenação de Aperfeiçoamento de Pessoal de Nível Superior*

### Types of psychoactive substance prevention programs in Brazil

Fifty-six studies were selected, including 41 articles, eight theses and seven dissertations. The first publication in the area appeared in 2011, with searches reaching up to December 2023. The highest frequency of publications was in the years 2021 (n = 11), 2016 (n = 8), 2020 (n = 8) and 2023 (n = 8). It can be seen that there was an increase in publications in the sequence of years following the implementation of programs coordinated by the macro-projects of the Ministry of Health and the National Secretariat for Drug Policy and Asset Management/Ministry of Justice, and evaluated in partnerships with public universities. The 15 theses or dissertations available in Brazilian catalogs were developed at the Federal University of Santa Catarina (UFSC; n = 06), the Federal University of São Paulo (UNIFESP; n = 05), the University of Brasília (UnB; n = 3) and Harvard University (n = 1).

Eleven preventive actions evaluated in Brazil were identified, of which nine are preventive programs. Of these, two have revised versions, which should be considered as separate programs. In addition to the programs, two preventive strategies were found. All of them will be described in more detail in the section on the characteristics of the programs analyzed: 1. Unplugged/#Tamojunto, evaluated in 19 studies, eight of which were theses and dissertations. In addition, six other articles analyzed its revised version, called #Tamojunto 2.0; 2. The Strengthening Families Program was evaluated in 10 publications, two of which were theses; 3. The Drug Abuse Resistance Education (DARE)/*Programa Educacional de Resistência às Drogas e à Violência* (PROERD) was evaluated in nine publications, one of which was a thesis; 4. The Good Behavior Game/ELOS was evaluated in six publications, three of which were theses or dissertations. In addition to these, there is another publication on its revised version, called ELOS 2.0. With only one publication, we have the SHAHRP (called in Brazil the Program to Stimulate Health and Reduce Risks Associated with Alcohol Use Applied to the Educational Environment/PERAE), the *Descolado* Program, the Women’s Program (Program M, presented in the form of a thesis), the Digital Wave Game (*Jogo da Onda Digital*) and the Drug Education and Social Skills intervention.

It is worth noting that the greatest concentration of research and production on this topic is in inter-institutional actions between the research groups at UNIFESP and UFSC, on the #Tamojunto and ELOS programs, totaling 25 collaborations, since even in the dissertations and theses from each university, professors from the others participated in the examination boards and the resulting articles together. UNIFESP still has the leading individual role in another 17 publications, albeit from different groups within the same institution. UnB has 10 publications, some of them in partnership with other institutions.

### Brazilian regions and sample of prevention programs on alcohol and other drugs in Brazil

Of the 56 studies selected, the majority were conducted with a nationwide sample (n = 27), covering more than one Brazilian region. In second place are the municipal implementations (n = 15), followed by the regional ones, predominantly in the Northeast (n = 7), and the state ones, developed in the state of São Paulo (n = 7). The most investigated regions were the Southeast (n = 39, 37 in the state of São Paulo), Northeast (n = 28, 22 in Ceará), South (n = 22, all in the state of Santa Catarina) and Midwest (n = 11, 10 in the Federal District). There were no studies carried out in the North.

The studies included in this review were mostly conducted with students/adolescents (n = 38), program facilitators/multipliers (n = 11), teachers (n = 8) and school pedagogical teams (n = 5), as well as with parents (n = 6). Predominantly, they were carried out in the context of schools (n = 44) or social services (n = 9), with one study carried out in a community setting (n = 1) and another in a mental health service (n = 1). The number of participants ranged from two to 6637 (mean = 2212; SD = 2513; median = 809). The majority of the studies were conducted with people of both sexes, with the exception of two which used an exclusively female sample^(12-13)^ and one which used an exclusively male sample^(14)^.

### Characteristics of drug use prevention programs evaluated in Brazil

With regard to carrying out preparatory studies for the implementation of preventive programs, only two mentioned a needs assessment. Three studies referred to evaluability studies, while 17 mentioned pilot studies. With regard to design, most of the studies were observational, generally associated with process evaluation (n = 25, five of which were dissertations and four theses), followed by experimental studies (n = 23), linked to effectiveness studies. Quasi-experimental studies were less frequent (n = 4) and are related to efficacy studies. Pre-experimental studies also totaled four (n = 4) and are related to temporal results. Quantitative (n = 23), mixed (n = 20) and qualitative (n = 13) approaches were used. The predominant temporality of the evaluations was longitudinal (n = 36).

Most of the studies evaluated the results of the programs (n = 30), including analyses of target outcomes (n = 22), or in addition to these, also of secondary, moderating or mediating effects of the results (n = 18). Six studies mentioned impact studies and none carried out a cost/benefit analysis. In the discussion of the results, 24 studies reported that the target outcomes improved to some extent, 10 reported that some outcomes worsened and 17 showed null effects on some of the target outcomes.

There were 26 studies aimed at evaluating the implementation process. Most of these studies evaluated different process outcomes, with the majority focusing on some type of fidelity evaluation (n = 22), with an emphasis on analyzing the exposure dimension, which includes checking the dose delivered, dose received and reach (n = 19). This was followed by studies that analyzed the quality of implementation (n = 8), team training (n = 3) and adherence (n = 4). In addition to fidelity, these studies also evaluated other process dimensions, 17 of which mentioned evaluating program satisfaction or acceptability, 12 feasibility studies for the Brazilian reality and 11 investigated internal and external contextual aspects that influenced implementation. Only one study analyzed the sustainability of the Strong Families Program. No studies were found on the dissemination of the programs.

Figure 2 describes the type of evaluation carried out: process or outcome, the methodological design and a summary of the results obtained with the preventive programs, according to the studies included in chronological order.

**Figure 2 -:** Methodological characteristics of the studies found that evaluated drug use prevention programs in Brazil according to author, type of evaluation, name of program/participants, study design and outcome. Florianópolis, SC and São Paulo, SP, Brazil, 2024

Author, Year	Program (Original name)	Type of evaluation	Design/Participants	Target outcome assessment
Rocha, 2011 ^( 13 )^	*Programa Mulheres*	Result	Quasi-experimental, longitudinal Women (n* = 273)	Increased self-efficacy in women’s interpersonal relationships and reduced drug use.
Shamblen et al., 2014 ^( 15 )^	PROERD ^†^ (DARE ^‡^ )	Result	Quasi-experimental, longitudinal Students, Adolescents (n* = 2995)	Harmless for effects on substance use, antisocial behavior and risk for substance use.
Horr, 2015 ^( 16 )^	#Tamojunto (Unplugged)	Process	Observational, Cross-sectional Students, Adolescents (n* = 353)	Positive satisfaction of the students when participating in the program, indicating good acceptability.
Peres, et al., 2015 ^( 17 )^	#Tamojunto (Unplugged)	Process	Observational, Cross-sectional Health professionals, teachers, program multipliers (n* = 19)	Implementation strengthened links between health and education. The program was highly acceptable to managers, coordinators, multipliers, health and education professionals and parents.
Sanchez, et al., 2016 ^( 18 )^	#Tamojunto (Unplugged)	Result	Quasi-Experimental (RCT ^§^ ), Longitudinal Students, Adolescents (n* = 2459)	Decrease in recent marijuana use among students aged 13 to 15. There was no evidence of an effect on students aged 11 to 12.
Medeiros, et al., 2016 ^( 19 )^	#Tamojunto (Unplugged)	Process	Observational, Longitudinal Students, teachers, multipliers and managers (n* = 84)	The implementation and monitoring rates of the lessons reached 94%. The duration of the lessons was insufficient to cover all the proposed activities. Teachers and students were satisfied with the program.
Schneider, et al., 2016 ^( 8 )^	ELOS (Good Behavior Game)	Process	Observational, Longitudinal Teachers, School Pedagogical Staff, Coaches (n* = 43)	High acceptance by teachers and principals, attributed to its classroom management strategy. There were indications of the need for adaptations to reflect the Brazilian social and economic context.
Strelow, 2016 ^( 20 )^	ELOS (Good Behavior Game)	Process	Observational, Cross-sectional Students, Adolescents(n* = 27)	Children showed high acceptability of ELOS and expressed the perceived results of increased collaboration with peers and a desire to continue the game.
Lopes, 2016 ^( 14 )^	#Tamojunto (Unplugged)	Process	Observational, Longitudinal Teachers (n* = 53)	Teachers gradually became more interested in the practice of prevention and the program. There was a change in discourse, from a moralistic and stigmatizing approach to a more understanding perspective.
Medeiros, 2016 ^( 21 )^	#Tamojunto (Unplugged)	Process	Observational, Longitudinal Students, Adolescents (n* = 1336)	94% of classes were implemented, but only 57% followed the manual. Some activities were excluded, there were difficulties in planning by teachers and a lack of support from administrators. They reported improvements in the classroom environment and in personal skills.
Martins, 2016 ^( 22 )^	#Tamojunto (Unplugged)	Result	Correlational, Cross-sectional Students, Adolescents (n* = 5007)	Analysis of the sample’s profile to understand possible moderators of the program’s effects identified a relationship between parenting styles and drug use.
Menezes, 2016 ^( 12 )^	*Famílias Fortes* (Strengthening Families Program)	Process	Observational, Cross-sectional Health professionals, facilitators, program multipliers (n* = 41)	The cultural adaptation was carried out well. However, the program needs to be adapted to aspects related to social inequalities and the illiteracy of the population, which implies further adaptations.
Sanchez, et al., 2017 ^( 23 )^	#Tamojunto (Unplugged)	Result	Experimental (RCT ^§^ ), Longitudinal Students, Adolescents (n* = 4253)	Improved outcome: delayed first use of inhalants. Outcome that worsened: first use of alcohol.
Peres, et al., 2017 ^( 24 )^	#Tamojunto (Unplugged)	Process	Observational, Cross-sectional Health professionals, education professionals (n* = 18)	Professionals’ positive perception of joint strategies for implementing the program and strengthening the connection between health and school. There were challenges in understanding intersectorality in practice.
Conegundes, 2017 ^( 25 )^	#Tamojunto (Unplugged)	Result	Experimental (RCT ^§^ ), Longitudinal Students, Adolescents (n* = 6387)	Students who participated in the intervention group and who did not live with their mothers had an increased chance of alcohol consumption when compared to the control group.
Pedroso, 2017 ^( 26 )^	#Tamojunto (Unplugged)	Process	Observational, Longitudinal Teachers, Facilitators, Multipliers (n* = 671)	For the sustainability and dissemination of the program as a public policy, it is recommended to invest in a paradigmatic change in the approach to drugs by the implementers, increase the use of interactive methodologies, adapt class time and expand intersectoriality.
Sanchez, et al., 2018 ^( 27 )^	#Tamojunto (Unplugged)	Result	Experimental (RCT§), Longitudinal Students, Adolescents (n* = 5007)	Results indicate that the program delayed the use of inhalants, but worsened the first use of alcohol among students who participated in the program.
Murta, et al., 2018 ^( 28 )^	*Famílias Fortes* (Strengthening Families Program SFP 10-14-UK)	Process	Observational, Longitudinal Students, Adolescents, Parents, Facilitators, Program Multipliers (n* = 33)	Program perceived as culturally relevant and partially clear. Cultural adaptations should focus on linguistic aspects of materials and procedures, taking into account cultural, economic and educational differences of families, conducted in a participatory manner.
Medeiros, et al., 2018 ^( 29 )^	#Tamojunto (Unplugged)	Process	Observational, Cross-sectional Students, adolescents, teachers, school staff (n* = 78)	Facilities for implementation include technical support, training, supervision and support from school administrators. The difficulties involved class time, obtaining support materials and complying with the regular curriculum. As a potentiality, improvements in coexistence were highlighted.
Gusmões 2018 ^( 30 )^	#Tamojunto (Unplugged)	Result	Experimental (RCT ^§^ ), Longitudinal Students, Adolescents (n* = 6637)	It reduced bullying in the first 9 months, but the effect was not maintained after 21 months. Risk factors for bullying included previous involvement in violence and use of alcohol and inhalants, mothers with drunken episodes.
D’Tôllis, 2018 ^( 31 )^	ELOS (Good Behavior Game)	Process	Observational, Longitudinal National managers (n* = 2)	Cultural adaptation was assessed as satisfactory. The aspects of implementation planning, cultural sensitivity, fidelity to the original program and evaluation and refinement processes were well developed.
Garcia, 2018 ^( 32 )^	ELOS (Good Behavior Game)	Process	Observational, Longitudinal Teachers, School Staff, Facilitators (n* = 60)	The evaluation of the fidelity of implementation was positive, but the results were partially inconclusive due to a problem in the return of the fidelity forms by teachers and multipliers.
Abdala, 2018 ^( 33 )^	*Famílias Fortes* (Strengthening Families Program)	Process	Observational, Cross-sectional Program Facilitators, Multipliers (n* = 26)	The barriers to implementation pointed out by the facilitators included inadequate working conditions, weak municipal administration, insufficient training methodologies and low professional adherence.
Sanchez, et al., 2019 ^( 34 )^	#Tamojunto (Unplugged)	Result	Experimental (RCT ^§^ ), Longitudinal Students, Adolescents (6391)	The results suggest that there is no effect of the program on normative beliefs in relation to drug use.
Murta, et al., 2020 ^( 7 )^	*Famílias Fortes* (Strengthening Families Program)	Result	Pre-experimental, Longitudinal Students, Adolescents, Parents and Facilitators, Program Multipliers (n* = 126)	Increase in learning self-efficacy and decrease in absence from school without parental permission. No effect on alcohol consumption, binge drinking episodes and antisocial behavior in the last month.
Abdala, et al., 2020 ^( 35 )^	*Famílias Fortes* (Strengthening Families Program)	Process	Observational, Cross-sectional Facilitators, Program Multipliers (n* = 26)	The barriers presented by the facilitators were working conditions, weak municipal administration, poor infrastructure, an inadequate group, methodologies for training facilitators, low adherence from managers and a lack of funding.
Valente, et al., 2020a ^( 36 )^	#Tamojunto (Unplugged)	Result	Experimental (RCT ^§^ ), Longitudinal Students, Adolescents (n* = 6391)	Results showed a reduction in decision-making skills and an increase in drug use and violence.
Valente, et al., 2020 ^( 37 )^	#Tamojunto (Unplugged)	Result	Experimental (RCT ^§^ ), Longitudinal Students, Adolescents (n* = 6391)	No potential “reinforcement” of parental behaviors regarding drug use was identified among those who received intervention. An association between lower parental demands and greater drug use by adolescents was identified.
Menezes, et al., 2020 ^( 38 )^	*Famílias Fortes* (Strengthening Families Program)	Process	Observational, Cross-sectional Facilitators, Program Multipliers (n* = 42)	Regarding fidelity, half of the facilitators reported excluding items from the procedures and 73.2% of the interviewees reported having made one or more modifications when implementing the program.
Murta, et al., 2020 ^( 39 )^	*Famílias Fortes* (Strengthening Families Program SFP 10-14-UK)	Process	Observational, Longitudinal Students, Adolescents, Parents (n* = 74)	Good levels of parental fidelity and involvement and moderate levels of adolescent involvement. The intervention is relevant and the procedures acceptable, but a need for cultural adaptations was identified to facilitate understanding for families with low levels of education.
Coelho and Monteiro, 2020 ^( 40 )^	*Jogo da Onda Digital* (Digital Wave Game)	Process	Observational, Cross-sectional Students, Adolescents (n* = 12)	Perception of improved understanding of topics related to drug use. Participants valued the interactive and playful approach of the game, which stood out in comparison to traditional educational approaches.
Valente, 2020 ^( 41 )^	#Tamojunto (Unplugged)	Result	Experimental (RCT ^§^ ), Longitudinal Students, Adolescents (n* = 6391)	The program changes decision-making skills, but in the opposite direction to that proposed by the program’s theoretical model. The intervention did not change drug use.
Amato et al., 2021 ^( 6 )^	*Programa PERAE* ^||^ (SHAHRP ^¶^ )	Process	Observational, Longitudinal Students, Adolescents (n* = 348)	Implementation fidelity reached 66.8%. The percentage of complete classes implemented ranged from 62.5% to 87.5%. Students and teachers reported good acceptability of the program.
Murta, et al., 2021 ^( 42 )^	*Famílias Fortes* (Strengthening Families Program)	Process	Observational, Longitudinal Students, Adolescents, Parents, Facilitators (n* = 410)	It was assessed that satisfaction/acceptability resulted in increased viability. The program improved family cohesion but worsened facilitator overload.
Valente and Sanchez, 2021 ^( 43 )^	PROERD ^†^ (DARE ^‡^ Keepin’ it REAL)	Result	Experimental (RCT ^§^ ), Longitudinal Students, Adolescents, (n* = 4030)	It improved the school experience, but worsened the intention to use drugs, decision-making skills, and skills in refusing drug offers.
Sanchez, et al., 2021 ^( 44 )^	#Tamojunto 2.0 (Unplugged)	Result	Experimental (RCT ^§^ ), Longitudinal Students, Adolescents (n* =5208)	Reduced the odds of 8th graders initiating alcohol use. No effects were found on any other drug initiation or prevalence of use/month.
Sanchez, et al., 2021 ^( 45 )^	PROERD ^†^ (DARE ^‡^ Keepin’ it REAL - kiR)	Result	Experimental (RCT ^§^ ), Longitudinal Students, Adolescents (n* = 4030)	It presented a negative effect, indicating that 7th grade students who already practiced binge drinking before and attended the program were more likely to maintain this use.
Cogo-Moreira, et al., 2021 ^( 46 )^	#Tamojunto (Unplugged)	Result	Experimental (RCT ^§^ ), Longitudinal Students, Adolescents (n* = 6390)	The results indicated that early violent behavior predicts more drug use among adolescents and shows the lack of evidence that #Tamojunto changes the dynamics between drug use and bullying.
Pinheiro-Carozzo, et al., 2021 ^( 47 )^	*Famílias Fortes* (Strengthening Families Program)	Result	Pre-experimental, Longitudinal Students, Adolescents, Parents (n* = 361)	It affects parenting dimensions differently depending on the initial parenting style and can help improve weaknesses in fulfilling parental duties in the short term.
Ferreira Junior, 2021 ^( 48 )^	PROERD ^†^ (DARE ^‡^ )	Result	Experimental (RCT§), Longitudinal Students, Adolescents (n* = 4045)	Alcohol use was associated with all types of bullying. Low academic performance was also strongly associated with bullying. Black/brown students were 3.35 times more likely to be highly victimized by bullying.
Mariano, et al., 2021 ^( 49 )^	ELOS 2.0 (Good Behavior Game)	Result	Experimental (RCT§), Longitudinal No information	Effectiveness study protocol.
Machado, et al., 2021 ^( 50 )^	*Educação Sobre Drogas e Habilidades Sociais*	Process	Observational, Longitudinal Students/Adolescents (n* = 145)	The majority of students approved of the program, favoring the possibility of continuing it in subsequent years.
Farias, 2021 ^( 51 )^	*Famílias Fortes* (Strengthening Families Program)	Process	Observational, Cross-sectional Managers (n* = 7)	Variables related to organizational capacity, program adaptation, political support, program evaluation and partnerships were cited as essential for program sustainability.
Gusmões et al., 2022 ^( 52 )^	PROERD ^†^ (DARE ^‡^ Keepin’ it REAL - kiR)	Result	Experimental, Longitudinal Students, Adolescents and PROERD Instructors (n* = 4030)	The level of implementation fidelity had no influence on the program’s ability to reduce drug use among adolescents. However, the qualitative analysis revealed adaptations made by police instructors.
Melo, et al., 2022 ^( 53 )^	#Tamojunto 2.0 (Unplugged)	Process	Observational, Longitudinal Teachers and Principals, Pedagogical Coordinators (n* = 21)	Low implementation fidelity, good quality in the application of the program and high student absenteeism.
Schneider, et al., 2022 ^( 54 )^	ELOS (Good Behavior Game)	Result	Quasi-experimental, longitudinal Students, Adolescents (n* = 1731)	Decreased aggression and disruptiveness among boys. It didn’t have the same effect on girls.
Valente, et al., 2022 ^( 55 )^	PROERD ^†^ (DARE ^‡^ , Keepin’ it REAL - kiR)	Result	Experimental, Longitudinal Students, Adolescents (n* = 4300)	There is no evidence of the effects of changes in the pattern of drug use when comparing intervention with control in any of the PROERD curricula aimed at 5th and 7th grade students.
Garcia-Cerde, et al., 2022 ^( 56 )^	#Tamojunto 2.0 (Unplugged)	Result	Experimental, Longitudinal Students, Adolescents (n* = 5208)	It increased knowledge about drugs and negative/non-positive beliefs about alcohol. No evidence was found on the effect of the program with marijuana.
Almeida, et al., 2023 ^( 57 )^	#Tamojunto 2.0 (Unplugged)	Result	Experimental, Longitudinalz Students, Adolescents (n* = 5208)	Factors such as: being a girl, older age, previous drug use and greater presence of mental health symptoms were the main predictors of drug use at follow-up.
Gusmões, et al., 2023 ^( 58 )^	PROERD ^†^ (DARE ^‡^ , Keepin’ it REAL - kiR)	Process	Observational, Cross-sectional Military Police (n* = 19)	Most instructors adapt the program. The main extrinsic reasons for changes were the challenges arising from the cultural reality and the school performance of the students and the lack of support from the school. The intrinsic reason was the performance of the instructors.
Valente, et al., 2023 ^( 59 )^	#Tamojunto 2.0 (Unplugged)	Result	Experimental, Longitudinal Students, Adolescents (n* = 5208)	It indirectly reduced bullying by reducing alcohol use. But the program was not directly effective in reducing the prevalence of bullying (victimization and perpetration).
Valente and Sanchez, 2023 ^( 60 )^	PROERD ^†^ (DARE ^‡^ , Keepin’ it REAL - kiR)	Result	Experimental, Longitudinal Students, Adolescents (n* = 4030)	It had no effect on decision-making skills, attitudes towards drugs, refusal skills and communication skills, as proposed by the theoretical model.
Garcia-Cerde, et al., 2023 ^( 61 )^	#Tamojunto 2.0 (Unplugged)	Result	Experimental, Longitudinal Students, Adolescents (n* = 5208)	It indirectly reduced lifetime alcohol use by increasing negative beliefs about alcohol. Although an indirect effect on decreasing binge drinking was observed, the direct effect on decreasing alcohol consumption was statistically significant.
Oliveira, et al., 2023 ^( 62 )^	*Descolado*	Process	Observational, Cross-sectional Program key informants (n* = 6)	The program can be evaluated. The results of the evaluation helped to organize and describe the program logically, as well as helping to improve its structure, indicating the need for adjustments to objectives, activities and resources.
Ferreira Junior, et al., 2023 ^( 63 )^	PROERD ^†^ (DARE ^‡^ , Keepin’ it REAL - kiR)	Result	Experimental, Longitudinal Students, Adolescents (n* = 4030)	Association between bullying outcomes, school violence, drug use patterns and socioeconomic descriptors.
Schneider, et al., 2023 ^( 34 )^	ELOS (Good Behavior Game)	Result	Pre-experimental, Longitudinal Students, Children (n* = 624)	Classes that received the program with greater fidelity showed more positive results in reducing target behaviors. Classes with low fidelity showed innocuous or possibly iatrogenic results.

*n = Number of participants; ^†^PROERD = Drug and Violence Resistance Education Program; ^‡^DARE = Drug Abuse Resistance Education; ^§^RCT = Randomized Clinical Trial; ^||^PERAE = Program to Stimulate Health and Reduce Risks Associated with Alcohol Use Applied to the Educational Environment; ^¶^SHAHRP = The School Health and Alcohol Harm Reduction Project

### Prevention programs on drug use in Brazil

#### Characteristics of preventive programs and strategies on drug use in Brazil

Of the eleven drug use prevention programs or strategies that have been evaluated in Brazil and identified in this review, seven are universal prevention programs, two are updated versions of previously evaluated programs, one is a selective prevention program and two are configured as preventive strategies. The delivery format was generally designed as a class or some pedagogical strategy, such as a workshop or game, as is the case with the ELOS Program and the Digital Wave Game. The average duration of daily implementation of the programs is 45 to 50 minutes, corresponding to one hour-lesson (ranging from 30 minutes to 2h30 minutes).

With regard to the target substance of the intervention, there is only one program that specifically addresses alcohol, which is the Program for Stimulating Health and Reducing Risks Associated with Alcohol Use Applied to the Educational Environment (PERAE), a cultural adaptation of SHAHRP. All the others address the consumption of alcohol and other drugs, especially tobacco, marijuana, inhalants, cocaine and crack. There are also those that don’t mention specific psychoactive substances. This is the case with the ELOS Program, as it is aimed at children and does not directly address alcohol and other drug use, only its medium and long-term preventive measures. Below is a summary of each program studied.

The most widely evaluated program in Brazil is #Tamojunto, a cultural adaptation of the European Unplugged program, carried out in schools with 8th grade students. Nineteen evaluation studies were identified, eight of which focused on process evaluation, including the acceptability of different actors, feasibility, fidelity, analysis of intersectorality and its inclusion in public policies^(14,16-17,19,21,24,26,29)^. In terms of evaluating results, there was one efficacy study related to the pilot implementation carried out in 2014^(18)^ and two effectiveness studies carried out during the 2016 implementation, one with a nine-month follow-up and the other with a 21-month follow-up^(23,27)^. Four studies addressed secondary effects, such as the relationship between drug use and bullying/school violence, parenting styles and binge drinking^(22,25,30,37)^. Three other studies evaluated the mediators of the program’s effects in the 2016 implementation^(34,36,41)^.

After the negative results, the program was revised between 2018 and 2019, defined as #Tamojunto 2.0, which was closer to the original content of #Tamojunto 2.0. There were six evaluative studies, one of which was a process evaluation focused on fidelity^(53)^, and an effectiveness study^(44)^ which showed that students exposed to the program were less likely to start using alcohol than those in the control group. Also investigated were the secondary effects on the relationship between drug use and bullying^(59)^, two mediation analysis studies that evaluated which mechanisms worked to achieve the program’s effects^(56,61)^ and a study of the moderation of the mental health condition and consumption pattern of the adolescents participating in the project^(57)^.

As for the ELOS Program: Building Collectives, a cultural adaptation of the North American program Good Behavior Game (GBG), six evaluative studies were found, four of them focused on process evaluation^(8,20,31-32)^. A study of temporal results mediated by fidelity, related to the 2014 implementation^(64)^ and a study of the effectiveness of the 2016 implementation, which pointed to a reduction in aggression and disruptiveness in boys (but not in girls), corroborating the international results of the GBG^(54)^. ELOS 2.0 was the result of a modification of some of the procedures of the first version of ELOS, and a record of the protocol of an effectiveness study has been located^(49)^.

The Strong Families Program is a cultural adaptation of the Strengthening Families Program (SFP), of English origin. Nine evaluative studies were found, including a needs assessment study for cultural adaptation^(28)^, a thesis on the process of cultural adaptation^(12)^ and a study on local adaptations for the Northeast^(38)^. Four studies evaluated the implementation process^(35,39,42,51)^. Two time-resolved studies showed an increase in self-efficacy for school learning, but no effect on changes in alcohol and drug use among adolescents^(7)^. On the other hand, there was an improvement in the relationship between parents and children and in parenting styles^(47)^.

PROERD is the most widely implemented preventive activity in schools in the country and is present in all Brazilian regions. Nine studies were found, the first of which was a quasi-experimental study from 2014, when PROERD still followed the model of the DARE program in the United States, without finding significant effects on the target results^(15)^. Three studies carried out in 2020-2021 analyzed results on the effectiveness of PROERD, already under the Keepin’it REAL model, which indicated no evidence of effects on changing the pattern of drug use, having worsened use in the form of binge drinking^(43,45,55)^. Analyses of the effects on bullying were carried out^(48,63)^ and mediation analyses found a worsening in communication skills, decision-making skills, attitudes towards drugs and refusal skills^(60)^. Studies have also evaluated the PROERD implementation process to understand the reasons for innocuous or potentially iatrogenic results^(52,58)^.

PERAE, a cultural adaptation of the Australian SHAHRP, was evaluated in a single study on the feasibility of the program for the Brazilian reality, with positive findings^(6)^. It is an Australian program, based on harm reduction for alcohol in young people, which has shown evidence in its country.

*Descolado*, created in 2018 by Recife City Hall in partnership with the Federal University of Pernambuco (UFPE), is a universal prevention program for the use of alcohol and other drugs, based on life skills and the Paulo Freire-inspired method, implemented in Recife schools. The study analyzed discusses the evaluability of *Descolado* ^(62)^.

The Digital Wave Game, an educational strategy on drugs, aims to contribute to preventive education through the teaching of science and biology. Originally developed in 2003^(65)^, it was adapted to the digital modality in 2019. The article found evaluated the process of the digital game^(40)^.

Program M (M, for *Mulher* “women”) is an intervention aimed at young women from low-income communities in the city of Rio de Janeiro, focused on changing attitudes, self-efficacy, knowledge and various risk behaviors for the health of these women, including the use of alcohol and other drugs. The program was analyzed in a doctoral thesis defended at Harvard University, through an effectiveness evaluation^(13)^.

The Drug Education and Social Skills strategy is an intervention carried out in state public schools in the interior of the state of Espírito Santo, with 10 thematic meetings on alcohol and other drugs and social skills in physical education classes. The study carried out a process evaluation^(50)^.

Figure 3 presents a detailed description of all the drug prevention programs identified in the review.

**Figure 3 -:** Description of drug use prevention programs evaluated in Brazil, according to program name, type of evaluation and category, theoretical model, intervention and outcome. Florianópolis, SC and São Paulo, SP, Brazil, 2024

**Program name**	**Type of evaluation**	**Type and category**	**Theoretical model**	**Intervention**	**Target outcome**
#Tamojunto	Process (n*=9) Result (n*=10)	Universal Prevention Program	Social learning theory, life skills, health belief model and critical attitude-action theory	Classes / 45-60 min / 12 meetings / held by trained teachers, in public schools, with the support of facilitators	Delaying the onset and reducing the pattern of use of alcohol, tobacco, marijuana, inhalants, cocaine and crack cocaine
#Tamojunto 2.0	Process (n*=1) Result (n*=5)	Universal Prevention Program	Social learning theory, life skills, health belief model and critical attitude-action theory	Classes / 45-60 min / 12 meetings / held by trained teachers, in public schools, with the support of facilitators	Delaying the onset and reducing the pattern of use of alcohol, tobacco, marijuana, inhalants, cocaine and crack cocaine
*Famílias Fortes*	Process (n*=8) Resultado (n*=2)	Universal Prevention Program	Family systems theory, social cognition theory, resilience model and socio-ecological models	Workshops / 2 to 2 hours and 30 minutes / 7 to 11 meetings, with parents and teenagers (10-14 years old), held at CRAS ^†^ , by SUAS ^‡^ professionals	Decrease adolescents’ use of alcohol and other drugs and modify parent-child relationship patterns and parenting styles
ELOS: *Construindo Coletivos*	Process (n*=5) Result (n*=1)	Universal Prevention Program	Life skills training, based on experimental behavior analysis	Pedagogical Strategy / 30 min. / varied frequency, usually 3 times a week	Modify aggressive and disruptive behavior and strengthen concentration on the task and collaboration among peers in children.
ELOS 2.0	Result (n*=1)	Universal Prevention Program	Life skills training, based on experimental behavior analysis	Pedagogical Strategy / 30 min. / varied frequency, usually 3 times a week	Modify aggressive and disruptive behavior and strengthen concentration on the task and collaboration among peers in children.
PROERD ^§^	Process (n*=2) Result (n*=7)	Universal Prevention Program	Social-emotional learning theory, social skills and the social influence model of drug education	Lessons / 50 min. / 10 meetings	Change the pattern of alcohol and other drug use
*Jogo da onda digital* (Digital Wave Game)	Result (n*=1)	Universal Prevention Strategy	Psychoeducation strategy and normative beliefs about drug use	Game / 2h / 1 meeting or more	Change the pattern of alcohol and other drug use
PERAE ^||^	Process (n*=1)	Universal Prevention Program	Socio-emotional Learning and Harm Reduction	Lessons / 1 hour / 8 meetings	Change the pattern of alcohol use
*Programa* *M* ( *M, de mulheres* )	Result (n*=1)	Selective Prevention Program	Bandura’s self-efficacy, attitude change, critical knowledge and perception of risk behavior	Manual and group discussions: video exposure, social media campaign / 18 meetings	Strengthening self-efficacy in interpersonal relationships of women impacted by alcohol and other drug use
*Descolado*	Avaliabilility (n*=1)	Universal Prevention Program	Paulo Freire Pedagogy, Life Skills	Classes in schools by teachers on drug use, youth protagonism and the student’s Personal Promotion Plan (PPP)	Change the pattern of alcohol and other drug use
*Educação Sobre Drogas e Habilidades Sociais*	Process (n*=1)	Universal Prevention Strategy	Psychoeducation	10 thematic meetings on alcohol and other drugs and social skills in Physical Education classes	Change the pattern of alcohol and other drug use

*n = Number of studies; ^†^CRAS = Social Assistance Reference Center; ^‡^SUAS = Unified Social Assistance System; ^§^PROERD = Educational Program for Resistance to Drugs and Violence; ^||^PERAE = Program to Stimulate Health and Reduce Risks Associated with Alcohol Use Applied to the Educational Environment

## Discussion

It could be argued that eleven is still a low number of drug use prevention strategies or programs evaluated in the country, with more than half of them lacking efficacy and effectiveness studies to help compose a more robust menu of evidence-based preventive actions. However, it is important to note that there are already programs that are consistently consolidated in their evidence, aimed at audiences at different stages of the life cycle (children, adolescents and families), which is an important starting point to support the implementation of public policies.

However, the intervention scenarios for the strategies and programs found are still limited, with the vast majority being in public schools or Social Assistance Reference Centers (CRAS). In this sense, it would be interesting to invest in the development or adaptation of community-based programs or systems, since community prevention involves the protagonism and co-responsibility of local leaders and the production of data on the specific needs of each territory, and is currently considered one of the most significant areas of action, as it affects local values and brings a sense of belonging to the participants^(65)^. Likewise, it is essential to invest more in environmental prevention, which is considered to be the most effective area of prevention. This approach acts broadly, covering the social landscape and with the objectives of reducing the availability of drugs, changing social norms and controlling advertising and marketing, among others. A successful example of this strategy was tobacco prevention, which began in 1986^(65)^.

In the review discussed here, evaluations were categorized as preventive strategies and programs, and prevention science proposes the existence of different levels of organization of preventive actions, distinguishing between them. Strategies are plans or approaches that have a theoretical underpinning and have undergone a process of methodological modeling and intervention aimed at tackling the targeted outcome in a more structured way. They can involve the integration of various coordinated actions guided by medium- or long-term objectives. Strategies can be more flexible in their use, although they must be evaluated and seek to be supported by evidence^(65)^. Preventive programs, on the other hand, are much more systematic than strategies, as they must have a well-structured logic model and/or theory of change, well-defined inputs (resources, efforts and inputs needed to implement an activity), objectives, outputs (immediate results) and expected outcomes (desired results in the medium and long term), as well as evaluation indicators. In addition, programs should preferably be manualized, i.e. have structured and objective guidelines for implementers (those who provide technical support and supervision) and participants, in order to guarantee fidelity of implementation. The programs must also be supported by evidence. All these two levels must organize training for those who are going to carry them out, in order to guarantee the quality of their implementation and build proposals for evaluating the process and the results^(5,65)^.

The classification of preventive actions according to the types of prevention is an important analyzer for making decisions about which program to implement, according to objectives aimed at the level of risk that the target population presents. Universal prevention is so called because it is aimed at the general population, without any stratification of risk factors. This type of prevention qualifies as programs when, for example, implemented in schools, they are aimed at all the students in a given classroom, without specifically selecting students who are more vulnerable to using alcohol and other drugs (AD). Selective prevention, on the other hand, is aimed at populations already at risk of problematic use of AD, which does not mean that these people are already using psychoactive substances, but rather that the action is directed at those most at risk of doing so. For example, programs carried out with children of recovering users or implemented in schools located in highly vulnerable areas where drug trafficking is rife. And finally, indicated prevention, aimed at people already identified as being at high risk of AD use, for whom there is already evidence of problems related to the use of psychoactive substances, with the aim of minimizing this use, reducing damage and improving quality of life, in order to promote health and social reintegration^(5,65)^. The review showed a predominance of universal prevention strategies and programs, making it necessary to reflect on these trends and question the possibility of diversifying the scope of actions in Brazilian public policies.

In its first and second versions, #Tamojunto was the most evaluated program, with cycles of studies on efficacy, effectiveness and fidelity, discussing aspects that played a role in producing or not producing the desired results, either when it had iatrogenic results, requiring it to be readjusted, or when, in the second version, it achieved the expected results by delaying the onset of alcohol use, as well as secondary effects on the practice of bullying. The iatrogenic results challenged analyses of aspects of the cultural adaptation process and the quality of the implementation process^(23,27)^, and “in a roundabout way” contributed to the growth of Brazilian prevention science, in that it required a network of researchers to analyze and learn from mistakes, reinforcing the need to monitor implementation and make adjustments to cultural sensitivity.

Several studies of #Tamojunto, whether in its first or second version, have dialogued with current requirements of advanced studies in prevention science^(66)^, such as the analyses of secondary effects, mediation and moderation of this program. The mediation studies sought to understand and analyze the mechanisms of the program’s logic model that actually acted to achieve the results obtained, and these analyses are important for resizing the program itself or for planning large-scale implementation^(34,36,41,56,61)^. The moderation analyses looked at contextual elements that interfered with the results, such as factors like the quality of the implementation, the psychosocial conditions of the students, etc^(57)^. These more robust analyses help to understand how programs work, helping to consolidate evidence and make decisions^(5,66)^.

ELOS, in its two versions, brought results of efficacy and effectiveness, with changes in aggressive and disruptive behavior and engagement in school tasks, as well as important results in reducing the personal vulnerabilities of children, which anticipates the possibility of improving life skills for the future of their existential trajectory. Its results were in line with those of other countries and showed evidence of developing life skills in children and reducing vulnerabilities and antisocial behavior, which could be an excellent action to be used with children, especially boys, as a mental health promotion strategy for a future with young people with better self-esteem and lower psychosocial risks^(54)^. The study on the moderation of implementation fidelity on temporal results showed that care in implementation is fundamental to obtaining the desired results^(64)^, corroborating what is assumed in implementation science and again corroborating the need to monitor preventive actions^(65)^.

*Famílias Fortes*, likewise, in its study of temporal results or effectiveness, produced an improvement in school self-efficacy, which can already be considered an important secondary effect^(7)^. There were also improvements in relationships between parents and children and in parenting skills and in the modification of negligent and authoritarian parenting styles, which is a very important result for strengthening family ties^(47)^, although there were no direct results on changing the pattern of adolescent alcohol use. Therefore, *Família Fortes* needs more research to prove its evidence.

Likewise, it was important to know that the most widely implemented preventive program in Brazilian schools, involving practically the entire national territory, PROERD, proved to be innocuous in most of the target outcomes or even iatrogenic^(15,43,45,55)^. In its evaluation in 2014, still under the DARE model, or in the 2020 evaluation, already under the Keepin’it Real model, it showed no effects on delaying the age of initial alcohol use, did not change the pattern of alcohol and other drug use and was iatrogenic for the binge drinking pattern among adolescents. It was also innocuous for the practice or victimization of bullying^(48,63)^. This finding should serve to alert municipal and state governments and managers of public and private schools to the need to request a redesign of this program.

With this, we highlight the merit of studies that have pointed to negative results, i.e. robust evaluations that show innocuous or iatrogenic effects of drug prevention programs, in order to indicate the need for changes in the content or methodology of the programs and to suggest alterations in their use in local or large-scale applications. The literature shows the importance of producing reflections and publishing these types of unintended results, in order to support program developers and public policy managers in improving their actions, considering the complexities involved in these negative results and recognizing the multiplicity of intended and unintended consequences when planning or evaluating interventions to prevent drug use^(67)^.

In short, it was found that there are programs that already have well-designed process evaluations, but have not yet carried out outcome evaluations, which points to the need to promote a new series of studies to verify their efficacy and effectiveness. In this sense, preventive public policies face important challenges, such as continuing the evaluations already started for some of the programs mentioned, as well as encouraging the development of new Brazilian preventive programs and systems.

However, one of the challenges of developing new programs is to carefully follow the stages of the prevention research cycle, which require time, the dedication of specialists and high levels of funding, insofar as it involves detailing the stages and processes for producing evidence, starting with needs assessment, development of the intervention and construction of its logic model and its evaluability, pilot testing, efficacy evaluation, evaluation of effectiveness, evaluation of the implementation process, dissemination studies and cultural adaptation to other contexts^(31-32,65)^. Another important requirement for the development of preventive interventions in Brazil is to build participatory processes that promote inclusion, in which “prevention ceases to be yet another mechanism for controlling and persecuting people and broadens its perspective towards actions based on comprehensive care and the dimension of ethics in its fullest sense”^(5)^. In this sense, they must be aligned with theories and methodologies that are consistent with the principles that govern the Unified Health System, the Unified Social Assistance System, the Education Network, Citizen Security, the Culture Network, among other sectors, in order to promote the integration of policies and develop intersectorality^(5,24)^.

The relevance of this review is to highlight the methodological characteristics and results obtained from evaluative studies of strategies and programs for alcohol and other drug use in Brazil, with the aim of contributing to the construction of a menu of preventive actions with evidence, which is necessary to support the implementation of preventive systems and public policies in this field. In this way, Brazil could begin to consolidate the development of a national platform for the certification of preventive strategies and programs, based on clear scientific standards, which would facilitate access to information on validated preventive actions for interested managers, professionals and scientists. This model has already been adopted in other countries, such as Blueprints for Healthy Youth Development^(68)^ in the United States, the European Union Drugs Agency’s Xchange Prevention Registry^(69)^, and *Evidência Viva* ^(70)^, recently created by the European Union Drugs Agency, in partnership with UNODC and the Brazilian government, to compile programs from Latin America. In addition, some websites developed in Latin American countries stand out, such as *Mi Brújula* ^(71)^, by the *Fundación San Carlos de Maipo* in Chile, and *Guía de Programas Preventivos del Consumo de Sustancias Psicoativas en Colombia* ^(72)^, by *Corporación Nuevos Rumblos*.

This shows the importance of public investment by a government in the development of a country’s science, especially in the area of prevention. In the context of scientific production related to findings that support public policies, it is interesting to analyze the investment of regions in the R+D model, which implies a clear relationship between research and the country’s development^(73)^. This investment promotes the advancement of an area of knowledge production and supports the large-scale implementation of evidence-based social technologies, bringing significant benefits for the promotion of the population’s health^(5)^. The main alcohol and other drug programs that have been analyzed and currently have efficacy and effectiveness indicators in our country were the result of these public incentives.

This moment of investment in prevention science by the Federal Government between 2013 and 2018 moved the academic, governance and public policy implementers^(5)^, resulting in the creation of the Brazilian Association for Prevention and Health Promotion (BRAPEP), which was formed to bring together researchers and professionals, advocating improvements in the area, both in research and evaluation, and in the implementation of public policies, with the agenda of expanding and qualifying preventive science in the country^(74)^.

In this sense, we highlight the strengthening of the relationship between public policy makers and managers and academia and researchers, stimulated by the governments of Presidents Lula and Dilma, who understood that the “systematization of knowledge and sensitivity to the Brazilian social question are not antagonistic. Science can and should be another instrument for transforming groups and countries”^(5)^. Brazil has thus gradually stood out for its academic productivity in the field of preventing the use of alcohol and other drugs, as analyzed, for example, in a previous bibliometric study on collaboration networks between Latin American and European Union countries in scientific productions in the field of AD, between 2001 and 2011, which showed a gradual increase in scientific collaboration between these two blocs of countries, with Brazil’s productivity standing out among Latin American countries^(73)^.

Similarly, the *Evidência Viva* website, which compiles evidence from programs in Latin America to establish recommendations, highlighted six programs with more robust evaluation processes, three of which are recommended (Unplugged/#Tamojunto2.0, Keepin’ it REAL, Good Behavior Game/ELOS), one that needs more evidence (Strengthening Families Program/*Familia Fortes*) and two that are not recommended (Unplugged/#Tamojunto, PROERD/D.A.R.E-kiR), using evidence produced by the studies analyzed here. Therefore, of the programs analyzed, five had their studies carried out in Brazil^(70)^.

Finally, some limitations of this study can be pointed out, including the issue of “silence” in the searches, i.e. when some publications related to the objective do not appear in the searches, even with the care taken in preparing the search equation. We tried to fill these gaps by consulting experts in the field. It was also a limitation not to have carried out a systematic analysis of the references of the articles found, which may have meant that publications may have slipped under our radar. Another difficulty was accessing the database of theses and dissertations: not all catalogs contain data from all postgraduate programs, which may imply that many works related to the topic may not have been included. In some cases, we opted for theses and dissertations in the form of articles, and these were found to have already been published, so as not to include the academic work, in order to avoid some repetition.

## Conclusion

Brazil, supported by public investment, has made progress in overcoming the lack of evidence in the field of alcohol and other drug use prevention. It already has a number of successful experiences in evaluating preventive strategies and programs, with some programs already having robust evidence available for public policies and those interested in preventing the use of alcohol and other drugs. The authorships and collaborations in the publications analyzed, as well as the creation of research associations, show the gradual consolidation of a network of researchers and experts in prevention. This network has the potential to contribute significantly to the development of evidence-based public policies, as well as strengthening the continuous production of knowledge through partnerships between academia and governments.

Most of the programs evaluated in this review are universal, aimed at adolescents, developed in schools and focused on various substances. Therefore, it is necessary to broaden the scope of the programs to include community-based prevention and environmental prevention. Likewise, it is necessary to direct efforts towards new population groups, such as young people and young adults, which is an age group with a high risk of harmful involvement in the use of psychoactive substances; indigenous populations, who have had a very harmful relationship with alcohol and drugs in Western consumer society. The development of selective and targeted programs should also be considered in the future planning of government projects, including medium and high-risk populations for the consumption of psychoactive substances. This scenario points to the importance of consolidating a science of prevention in Brazil that is supported by evidence, culturally sensitive and sustainable, contributing to the formulation of more structured and impactful public policies.
