# Supplementary material for: Methodological characteristics of evaluative studies of drug prevention programs in Brazil: scoping review
Source: Rev Lat Am Enfermagem. 2025 Jul 11;33:e4578. doi: 10.1590/1518-8345.7709.4578 (PMC12259218; doi:10.1590/1518-8345.7709.4578)
Supplement: Translation

# Translation: Características metodológicas de estudos avaliativos de programas preventivos para drogas no Brasil: revisão de escopo ^*^

**DOI:** 10.1590/1518-8345.7709.4579

**Authors:** Daniela Ribeiro Schneider, Charlene Fernanda Thurow, Tallita Franzoloso, Leila Gracieli da Silva, Guilherme Gomes Silva, Elaine Lucas dos Santos, Liz Paola Domingues, Leila Pimentel dos Anjos, Fernanda Machado Lopes, Ana Regina Noto

**Affiliations:** 1 Universidade Federal de Santa Catarina, Departamento de Psicologia, Florianópolis, SC, Brasil.; 2 Bolsista do Conselho Nacional de Desenvolvimento Científico e Tecnológico (CNPq), Brasil.; 3 Bolsista da Coordenação de Aperfeiçoamento de Pessoal de Nível Superior (CAPES), Brasil.; 4 Universidade Estadual do Norte do Paraná, Departamento de Enfermagem, Jacarezinho, PR, Brasil.; 5 Universidade Federal de São Paulo, Departamento de Psicobiologia, São Paulo, SP, Brasil.

**Keywords:** Programa, Prevenção, Revisão, Abuso de Drogas, Brasil, Políticas Públicas

## Abstract

mapear estudos que desenvolveram processos avaliativos de programas de prevenção para o uso de álcool e outras drogas no Brasil, analisando suas características metodológicas.

revisão de escopo orientada pelo *Joanna Briggs Institute Manual* e pelo *Preferred Reporting Items for Systematic reviews and Meta-Analyses extension for Scoping Reviews*. Realizou-se as buscas em oito bases de dados e um banco de teses e dissertações, triados com duplo-cego no *Rayyan* ^®^, analisados com foco na metodologia dos estudos avaliativos e por meio de síntese qualitativa.

das 56 publicações incluídas, a maioria utilizou desenho observacional, em geral, relacionado às avaliações de processo (n = 26), seguidas pelo experimental (n = 23) e quasi-experimental (n = 8), nas análises de resultados em termos de efetividade e eficácia, respectivamente. Das onze ações analisadas, oito são programas de prevenção universal, um de prevenção seletiva e dois se configuram como estratégia preventiva.

a maioria dos programas avaliados é de caráter universal, direcionados a adolescentes, realizados em escolas e focados em várias drogas. Recomenda-se focar em novos grupos, como em populações indígenas e em outras condições de risco, além do desenvolvimento de programas seletivos em futuras iniciativas governamentais para prevenir o uso de drogas no Brasil.

## Destaques

**(1)** Localizaram-se nove programas preventivos a drogas com processos de avaliação no Brasil. **(2)** Dos nove programas, oito são de prevenção universal e um de prevenção seletiva. **(3)** Alguns programas têm evidências consistentes para subsidiarem políticas públicas. **(4)** O PROERD não mostrou efetividade na prevenção, precisando de remodelação. **(5)** Há necessidade de ampliar o escopo dos programas preventivos com evidências no Brasil.

## Introdução

Desde a década de 1970, a prevenção tem se consolidado gradualmente como uma ciência fundamentada em bases teóricas consistentes e no rigor metodológico. A construção de evidências tornou-se essencial para assegurar a eficácia das ações preventivas e o alcance dos resultados esperados, com o objetivo de formular políticas públicas que priorizem a qualidade, a economicidade e a promoção da saúde. Nesse contexto, é indispensável que os países adotem essa perspectiva científica para garantir a qualidade de suas políticas^(1)^.

No Brasil, embora muitas ações preventivas sejam realizadas, a avaliação para comprovar a eficácia dessas iniciativas não é tão comum. Esse fato, somado ao caráter pontual das intervenções, dificulta que a área promova suas evidências de maneira eficaz e mostra que o processo de consolidação da ciência da prevenção no país ainda está em estágio emergente^(1)^. Um dos primeiros estudos de revisão sobre programas preventivos ao uso de drogas no Brasil reuniu pesquisas publicadas entre os anos de 1991 e 2001 e concluiu que as ações de prevenção na década de 1990 foram impulsionadas pelo modelo de prevenção adotado para enfrentar os desafios das Doenças Sexualmente Transmissíveis^(2)^. Essas ações receberam críticas por seu caráter reducionista, focado apenas na doença, seguindo a lógica da “guerra às drogas” que predominava nesse campo.

Nesse contexto, começaram a ampliar o olhar para a relação entre as vulnerabilidades pessoais e contextuais e apareceram os primeiros projetos sustentados na lógica da redução de danos. A produção científica ainda era muito voltada aos aspectos farmacológicos da droga em si e ao tratamento da dependência. A maioria dos projetos preventivos analisados tinha por objetivo atingir a escola, influenciados por especialistas que advogavam o espaço escolar como privilegiado para o desenvolvimento deste tipo de intervenção. Prevalecia o desenvolvimento de projetos-piloto em escolas públicas monitoradas pelo Ministério da Saúde, porém que ainda usavam metodologias tradicionais, sustentadas em palestras e passagem de informações, realizadas principalmente por agentes extraescolares, como médicos e policiais. Nessa revisão, realizada até o ano de 2001, não apareceram estudos de eficácia e efetividade dos programas analisados^(2)^.

Em revisão sistemática posterior, com artigos publicados até o ano 2012, foram analisadas publicações de avaliação de programas em saúde mental no Brasil, incluindo problemas de comportamento, violência, abuso sexual, abuso de álcool e drogas e vazio existencial^(3)^. Encontrou-se uma predominância de estudos pré-experimentais e quasi-experimentais, sem avaliações de *follow-up* e com amostras pequenas. Boa parte das avaliações eram de processo ou de levantamento de necessidades, ou ainda testes-pilotos de programas, sendo escassos os estudos para avaliação de eficácia, efetividade e difusão^(3)^. Apareceu, até aqui, uma certa evolução da ciência da prevenção no cenário brasileiro, mas ainda incipiente.

Outro estudo mais recente, publicado em 2020, desenvolvido com 1151 dirigentes de escolas, sobre ações e programas preventivos realizados em escolas públicas e privadas brasileiras, constatou que os programas ainda são implementados sem regularidade e com curta duração, dirigidos principalmente aos alunos, utilizando diferentes modelos teóricos e promovidos principalmente pela Polícia Militar^(4)^. Sendo assim, mais de três décadas de pesquisas e revisões mostram a evolução gradativa da ciência da prevenção brasileira, que ainda enfrenta muitas dificuldades e precisa de mais incentivos para o seu desenvolvimento.

O ano de 2013 foi um marco para a o fortalecimento da ciência da prevenção brasileira, devido a uma importante iniciativa do Governo Federal que impulsionou as principais pesquisas e implementações de programas baseados em evidência no país, contribuindo para mudar o cenário da ciência da prevenção no país. A ação foi realizada em parceria entre a Coordenação de Saúde Mental, Álcool e Outras Drogas, do Ministério da Saúde, com o Escritório das Nações Unidas sobre Drogas e Crimes (UNODC), a Secretaria Nacional de Políticas sobre Droga (SENAD), do Ministério da Justiça e a Fundação Oswaldo Cruz. Três programas sofreram, então, processos de adaptação cultural ao Brasil, o ELOS: Construindo Coletivos (ELOS) (do original em inglês *Good Behavior Game*), o #tamojunto (*Unplugged*) e o Fortalecendo Famílias (*Strengthening Families Programme*), buscando abranger ações preventivas ao longo do ciclo vital. O primeiro é voltado para a infância, o segundo para a adolescência e o terceiro para o fortalecimento das relações parentais no âmbito das famílias^(5)^. Iniciativas independentes também ganharam espaço, como a adaptação brasileira do Programa australiano *The School Health and Alcohol Harm Reduction Project* (SHAHRP)^(6)^. A experiência gerou um processo bem mais consistente e robusto de produção de evidências, fortalecendo a relação entre aqueles que formulam e implementam políticas preventivas e os institutos de pesquisa que avaliam estas iniciativas^(5)^. Por outro lado, essa experiência foi desafiadora, pois a importação de programas estruturados pode trazer dificuldades relacionadas às adaptações culturais e à realidade local^(7-8)^. Isso pode resultar em perda de evidências devido a alterações nos desfechos, que podem se tornar desfavoráveis, além de comprometer a fidelidade aos aspectos centrais do programa original e enfrentar os desafios inerentes à implementação em larga escala^(5)^. Sendo assim, fortalecer os processos de avaliação é uma necessidade e um grande desafio.

Compreender melhor como foram os avanços na implementação de ações preventivas ao uso de drogas e promoção de saúde no Brasil faz-se necessário para subsidiar a implementação de políticas públicas, tais como o Sistema Nacional de Prevenção do Uso de Álcool e Outras Drogas, ligada à SENAD. Sendo assim, um estudo detalhado das características metodológicas das estratégias e programas para uso de álcool e outras drogas que já passaram por processos avaliativos pode auxiliar na oferta de um cardápio de ações preventivas, necessário para a implementação das políticas preventivas. Desse modo, o Brasil poderia começar a consolidar o desenvolvimento de uma plataforma para certificação de estratégias e programas, com base em padrões científicos claros, como já acontece em outros países. Da mesma forma, esta análise auxilia para que a ciência da prevenção brasileira, ainda em processo de amadurecimento, possa olhar para sua história e pensar sobre seus avanços e seus desafios.

Ao considerar a problemática acima descrita, esta atual revisão de escopo objetiva mapear estudos que desenvolveram processos avaliativos de programas de prevenção para o uso de álcool e outras drogas no Brasil, analisando suas características metodológicas, publicados entre os anos de 2011 e 2023. Busca contribuir para a elaboração de um cardápio de iniciativas preventivas baseadas em evidências disponíveis no Brasil, que possa subsidiar políticas públicas preventivas sobre drogas e auxiliar na tomada de decisões de gestores, profissionais e pesquisadores.

## Método

### Tipo de estudo

A revisão de escopo foi o método escolhido para responder ao objetivo desta pesquisa por possibilitar o mapeamento da literatura existente em um determinado campo e sistematizar características conceituais e metodológicas de pesquisas^(9)^. Esta revisão foi orientada pelo *Joanna Briggs Institute Manual* e pelas diretrizes do *Preferred Reporting Items for Systematic reviews and Meta-Analyses extension for Scoping Reviews Checklist* (PRISMA-ScR)^(10)^ e realizou as seguintes etapas: 1) elaboração do protocolo (registrado no PROSPERO sob o número 2022 CRD42022330854, link de acesso: https://www.crd.york.ac.uk/prospero/display_record.php?ID=CRD42022330854), com especificação da pergunta de pesquisa, definição dos critérios de elegibilidade, das bases de dados e das estratégias de busca; 2) identificação dos estudos relevantes aplicando as estratégias de busca nas bases de dados selecionadas; 3) seleção dos estudos com base nos critérios de elegibilidade; 4) extração e análise os dados; 5) sistematização e resumo dos dados e relato dos resultados. O protocolo (disponível mediante solicitação à autora correspondente) foi desenvolvido pela equipe de pesquisa, composta por especialistas na ciência da prevenção em psicologia, profissionais com experiência em revisões sistemáticas na área da saúde e por um bibliotecário.

A pergunta de pesquisa que guiou esta revisão foi: Quais são as características metodológicas dos estudos que investigaram programas de prevenção sobre uso de drogas avaliados no Brasil? Os componentes da pergunta seguiram o acrônimo PCC (item 4 do PRISMA ScR), em que os “participantes” (P) foram estudos empíricos completos conduzidos com seres humanos; “conceito” (C) foram os programas de prevenção sobre drogas que passaram por algum processo de avaliação (de efeito temporal, eficácia, efetividade ou de processo de implementação); e “contexto” (C) foi o brasileiro.

### Critérios de seleção e cenário

Foram incluídos estudos experimentais e quase experimentais, estudos metodológicos, estudos observacionais analíticos e descritivos, abordagens qualitativas, teses e dissertações, cuja temática central fosse a aplicação de um programa de prevenção sobre drogas avaliado do Brasil. Foram incluídos estudos publicados entre 1/1/2010 e 31/12/2023, sem restrições de idioma. Foram excluídos estudos com população não humana (população errada); editoriais, cartas, erratas, livros, capítulos de livro e trabalhos apresentados em congresso (tipo de publicação errada); estudos teóricos e revisões de qualquer natureza - integrativa, narrativa, sistemática, meta-análise (desenho do estudo errado); estudos que investigaram programas de prevenção sobre outro foco que não drogas, ou que não apresentaram resultados de avaliação do programa, ou ainda que não fossem conduzidos no Brasil (tema errado).

A busca sistemática foi realizada em dois momentos: a primeira, em 31 de agosto de 2021, quando se iniciou a presente revisão, e uma atualização em 31 de janeiro de 2024. As bases de dados consultadas foram: *Medical Literature Analysis and Retrieval System Online* (Medline) *via Publisher MEDLINE* (PubMed); *Excerpta Medica Database* (EMBASE); *Psychological Information Database* (PsycINFO); *Web of Science*, *Scientific Electronic Library Online* (SciELO); Literatura Latino-Americana e do Caribe em Ciências da Saúde (LILACS) e Índice de Literatura Técnico-Científica em Psicologia (IndexPsi), ambas parte da Biblioteca Virtual em Saúde - Psicologia Brasil (BVS-Psi Brasil); a provedora de bases de dados *Elton B. Stephens Company* (EBSCO); e Banco de Teses e Dissertações da Coordenação de Aperfeiçoamento de Pessoal de Nível Superior (CAPES). As bases foram escolhidas considerando a sua abrangência mundial com foco multidisciplinar (ex. *Web of Science*), com amplo alcance de artigos na área da saúde (ex. *MEDLINE* via PubMed) e na psicologia (ex. PsycINFO), e pela característica de indexarem artigos publicados em revistas da América Latina e Caribe (ex. LILACS e SciELO), já que o foco era em estudos realizados no Brasil.

Os indexadores utilizados nas buscas foram “*Program*”, “*Prevention*”, “*Evaluation*”, “*Drug*”, e “Brazil”, e as estratégias de busca completas, incluindo os descritores e operadores *booleanos* utilizados, podem ser acessados em https://www.crd.york.ac.uk/PROSPEROFILES/330854_STRATEGY_20220506.pdf. Além das bases de dados, foram consultados especialistas no tema da ciência da prevenção do Brasil para reduzir o viés de seleção e ampliar as possibilidades de acessar o maior número de estudos sobre o tema. Não foram verificadas sistematicamente todas as referências dos artigos incluídos, pois em suas análises preliminares não apareceram artigos novos dentro de nosso período delimitado de busca.

As referências obtidas a partir das buscas nas bases de dados foram importadas com o auxílio do *EndNote*, onde as duplicatas foram excluídas. Posteriormente, as referências foram importadas para o *Rayyan*®^(11)^ para a seleção dos estudos relevantes aplicando os critérios de elegibilidade, em formato duplo-cego.

### Coleta, tratamento e análise de dados

Os dez primeiros resumos foram avaliados em reunião *online* com toda equipe, por meio do *Rayyan*®, para uniformizar a lista de razões de exclusão. Posteriormente, oito juízas independentes fizeram a triagem dos estudos em duplo-cego a partir da leitura dos demais títulos e resumos. As discrepâncias foram resolvidas em reunião com pelo menos 50% da equipe até se chegar a um consenso. Após a triagem, o mesmo procedimento foi utilizado pelas oito juízas para a calibragem da leitura e extração dos textos completos.

Para a extração dos dados, a equipe de pesquisa elaborou uma planilha matriz no *software Excel*, com bases nas diretrizes do PRISMA-ScR. A planilha foi organizada em cinco grandes seções para extrair dados: 1) Caracterização geral dos estudos (ano da publicação e delineamento); 2) Método dos estudos, como especificação do público-alvo (número e sexo), uso de instrumentos de avaliação antes e após a intervenção, procedimentos (ex. fez *follow-up*) e desfechos avaliados (tipo de droga, padrão de consumo); 3) Dados sobre o programa de prevenção sobre drogas, como o nome do Programa, o contexto (ex. escolar, saúde), a abrangência (ex. nacional, regional) e os procedimentos (número de sessões e duração, tipo de droga que o programa é direcionado, tipo – oficina, palestra, vivência – e categoria de prevenção); 4) Dados sobre os procedimentos de avaliação do processo de implementação do programa, ou seja, se foram realizados estudos preparatórios (como levantamento de necessidades e estudos-piloto), estudos de fidelidade, satisfação, viabilidade e sustentabilidade; e 5) Dados sobre a avaliação dos resultados do programa, ou seja, desfechos que melhoraram, pioraram e se mantiveram, e dados sobre eficácia, efetividade e custo-benefício.

Ao se considerar que esta é uma revisão de escopo, que busca apresentar de forma sumarizada as características dos estudos mapeados de programas de prevenção sobre drogas realizados no Brasil, não foram aplicadas ferramentas de análise de qualidade metodológica, risco de viés e qualidade da evidência, pois estes itens são aplicáveis a revisões sistemáticas que visam avaliar efeitos de intervenções.

## Resultados

A partir das buscas nas bases selecionadas, foram identificados 2106 textos, sendo 1631 nas buscas realizadas na data de 31/08/2021 (textos de 1/01/2010 a 31/08/2021) e 475 nas buscas realizadas na data de 31/01/2024 (textos de 01/09/2021 a 31/12/2023). Foram somados, ainda, 44 textos indicados a partir de consulta a especialistas (com o uso dos mesmos descritores), em dois momentos, setembro de 2021 e janeiro de 2024, totalizando 2150 publicações encontradas. Nenhum material foi incluído a partir de leitura de referências dos artigos selecionados. Após a exclusão de 296 estudos duplicados (178 na primeira e 118 na segunda busca), 1854 seguiram para a etapa de triagem. Aplicados os critérios de inclusão a partir da leitura dos resumos (razões de exclusão detalhadas na Figura 1), 84 estudos seguiram para etapa de leitura de texto completo. Destes, 28 textos foram eliminados pelos critérios de exclusão, restando as 56 publicações incluídas para a análise e síntese qualitativa. A Figura 1 apresenta o fluxograma com detalhes deste processo.

**Figura 1 -:** Fluxograma das etapas de busca, triagem, elegibilidade e inclusão

*n = Número de estudos; ^†^ *Medline* via *PubMed* = *Medical Literature Analysis and Retrieval System Online via Publisher Medline*; ^‡^EMBASE = *Excerpta Medica Database*; ^§^PsycINFO = *Psychological Information Database*; ^||^LILACS = Literatura Latino-Americana e do Caribe em Ciências da Saúde; ^¶^ *IndexPsi* = Índice de Literatura Técnico-Científica em Psicologia; **EBSCO = *Elton B. Stephens Company*; ^††^SciELO = *Scientific Electronic Library Online*; ^‡‡^CAPES = Coordenação de Aperfeiçoamento de Pessoal de Nível Superior

## Tipos de Programas de prevenção sobre substâncias psicoativas no Brasil

Foram selecionados 56 estudos, sendo 41 artigos, oito teses e sete dissertações. A primeira publicação na área apareceu em 2011 chegando as buscas até dezembro de 2023, sendo que a maior frequência de publicações foi nos anos de 2021 (n = 11), 2016 (n = 8), 2020 (n = 8) e 2023 (n = 8). Nota-se que houve um aumento de publicações na sequência de anos posteriores às implementações de programas coordenados pelos macroprojetos do Ministério da Saúde e Secretaria Nacional de Políticas sobre Drogas e Gestão de Ativos/Ministério da Justiça, e avaliados em parcerias com universidades públicas. As 15 teses ou dissertações disponíveis em catálogos brasileiros foram desenvolvidas nas Universidades Federal de Santa Catarina (UFSC; n = 06), Universidade Federal de São Paulo (UNIFESP; n = 05), Universidade de Brasília (UnB; n = 3) e Universidade de Harvard (n = 1).

Foram identificadas onze ações preventivas avaliadas no Brasil, das quais nove são programas preventivos. Dentre esses, dois possuem versões revisadas, que devem ser consideradas como programas distintos. Além dos programas, foram encontradas duas estratégias preventivas. Todos serão descritos em maiores detalhes na seção sobre as características dos programas analisados: 1. O *Unplugged*/#Tamojunto, avaliado em 19 estudos, sendo oito em forma de teses e dissertações. Somam-se a estes outros seis artigos que analisaram sua versão revisada, chamada de #Tamojunto 2.0; 2. O *Strengthening Families Program*/Famílias Fortes foi avaliado em 10 publicações, sendo duas em forma de tese; 3. O *Drug Abuse Resistance Education* (DARE)/Programa Educacional de Resistência às Drogas e à Violência (PROERD) foi avaliado em nove publicações, sendo uma tese; 4. O *Good Behavior Game*/ELOS foi avaliado em seis publicações, sendo três teses ou dissertações. Soma-se a estas mais uma publicação sobre a sua versão revisada, chamada de ELOS 2.0. Com uma única publicação, temos o SHAHRP (chamado no Brasil de Programa de Estímulo à Saúde e Redução de Riscos Associados ao Uso de Álcool Aplicado ao Ambiente Educacional/PERAE), o Programa Descolado, o Programa Mulheres (Programa M, apresentado em forma de tese), o Jogo da Onda Digital e a intervenção Educação Sobre Drogas e Habilidades Sociais.

Destaca-se que a maior concentração de investigação e produção na temática está em ações interinstitucionais entre os grupos de pesquisa da UNIFESP e UFSC, sobre os programas #Tamojunto e ELOS, totalizando 25 colaborações, pois, mesmo nas dissertações e teses de cada universidade, os professores das outras participavam das bancas de exame e dos artigos resultantes em conjunto. A UNIFESP ainda tem o protagonismo individual em outras 17 publicações, ainda que de grupos diferentes dentro da mesma instituição. A UnB tem 10 publicações, algumas delas em parcerias com outras instituições.

### Regiões brasileiras e amostra dos Programas de prevenção sobre álcool e outras drogas no Brasil

Dos 56 estudos selecionados, a maioria foi conduzida com amostra de abrangência nacional (n = 27), abarcando mais de uma região brasileira. Em segundo lugar estão as implementações de âmbito municipal (n = 15), seguida das regionais, predominantemente no Nordeste (n = 7), e pelas estaduais, desenvolvidas no estado de São Paulo (n = 7). As regiões mais investigadas foram Sudeste (n = 39, sendo 37 no estado de São Paulo), Nordeste (n = 28, sendo 22 no Ceará), Sul (n = 22, na sua totalidade do estado de Santa Catarina) e Centro-Oeste (n =11, sendo 10 no Distrito Federal). Nenhum estudo foi desenvolvido na Região Norte.

Os estudos incluídos nesta revisão foram conduzidos majoritariamente com estudantes/adolescentes (n = 38), facilitadores/multiplicadores dos Programas (n = 11), professores (n = 8) e equipes pedagógicas das escolas (n = 5), assim como com pais (n = 6). Predominantemente, foram desenvolvidos no contexto escolar (n = 44) ou da assistência social (n = 9), com um estudo realizado no âmbito comunitário (n = 1) e outro em serviço de saúde mental (n = 1). O número de participantes variou de dois a 6637 (Média = 2212; *DP* = 2513; mediana = 809). Majoritariamente foram conduzidos com pessoas de ambos os sexos, à exceção de dois que usaram amostra exclusivamente feminina^(12-13)^ e um exclusivamente masculina^(14)^.

### Características dos Programas de prevenção sobre uso de drogas avaliados no Brasil

Em relação à realização de estudos preparatórios para a implementação dos Programas preventivos, apenas dois mencionam o levantamento de necessidade. Três estudos referiram-se a estudos de avaliabilidade, enquanto 17 mencionaram a realização de estudos pilotos. Sobre o delineamento, em sua maioria, o desenho dos estudos foi observacional, geralmente associado à avaliação de processo (n = 25, sendo cinco advindos de dissertações e quatro de teses); em seguida vêm os experimentais (n = 23), ligados aos estudos de efetividade. Os estudos quasi-experimentais foram menos frequentes (n = 4) e estão relacionados aos estudos de eficácia. Os estudos pré-experimentais também totalizam quatro (n = 4) e estão relacionados aos resultados temporais. Foram utilizadas abordagens quantitativas (n = 23), mistas (n = 20) e qualitativas (n = 13). A temporalidade predominante das avaliações foi longitudinal (n = 36).

A maioria dos estudos avaliou os resultados dos Programas (n = 30), incluindo análises de desfechos alvos (n = 22), ou além deles, também seus efeitos secundários, moderadores ou mediadores dos resultados (n = 18). Seis estudos mencionaram a realização de estudos de impacto e nenhum fez análise de custo/benefício. Na discussão dos resultados, 24 estudos reportaram que os desfechos alvos melhoraram em alguma medida, 10 informaram que os alguns desfechos pioraram e 17 demonstraram efeitos nulos sobre alguns dos desfechos visados.

Houve 26 estudos direcionados à avaliação do processo de implementação. Grande parte desses trabalhos realizaram avaliações de diferentes desfechos nos processos, sendo que a maioria se dedicou a algum tipo de avaliação de fidelidade (n = 22), com ênfase na análise da dimensão da exposição, que inclui a verificação da dose fornecida, dose recebida e alcance (n = 19). Seguiram, assim, os estudos que analisaram a qualidade da implementação (n = 8), a formação da equipe (n = 3) e aderência (n = 4). Além da fidelidade, os trabalhos também avaliaram outras dimensões de processo, dos quais 17 mencionaram avaliação de satisfação ou aceitabilidade dos programas, 12 estudos de viabilidade para a realidade brasileira e 11 investigaram aspectos do contexto internos e externos que influenciaram a implementação. Um único estudo analisou a sustentabilidade do Programa Famílias Fortes. Não foram encontrados estudos sobre a difusão dos Programas.

A Figura 2 apresenta a descrição do tipo de avaliação realizado: processo ou resultado, o delineamento metodológico e uma síntese dos resultados obtidos com os programas preventivos, conforme os estudos incluídos em ordem cronológica.

**Figura 2 -:** Características metodológicas dos estudos encontrados que realizaram avaliação de programas para prevenção ao uso de drogas no Brasil segundo autor, tipo de avaliação, nome do programa/participantes, delineamento e desfecho do estudo. Florianópolis, SC e São Paulo, SP, Brasil, 2024

**Autor, Ano**	**Programa (Nome Original)**	**Tipo de Avaliação**	**Delineamento/Participantes**	**Avaliação de desfecho-alvo**
Rocha, 2011 ^( 13 )^	Programa Mulheres	Resultado	Quasi-Experimental, Longitudinal Mulheres (n* = 273)	Aumento da autoeficácia nas relações interpessoais de mulheres e redução do consumo de drogas.
Shamblen et al., 2014 ^( 15 )^	PROERD ^†^ (DARE ^‡^ )	Resultado	Quasi-Experimental, Longitudinal Estudantes, Adolescentes (n* = 2995)	Inócuo para efeitos no uso de substâncias, comportamento antissocial e de risco para uso de substâncias.
Horr, 2015 ^( 16 )^	#Tamojunto ( *Unplugged* )	Processo	Observacional, Transversal Estudantes, Adolescentes (n* = 353)	Satisfação positiva dos educandos ao participar do programa, indicando boa aceitabilidade.
Peres, et al., 2015 ^( 17 )^	#Tamojunto ( *Unplugged* )	Processo	Observacional, Transversal Profissionais da saúde, professores, multiplicadores do programa (n* = 19)	A implementação fortaleceu vínculos entre saúde e educação. O programa teve alta aceitabilidade por gestores, coordenadores, multiplicadores, profissionais da saúde e educação e pais.
Sanchez, et al., 2016 ^( 18 )^	#Tamojunto ( *Unplugged* )	Resultado	Quasi-Experimental (ECR ^§^ ), Longitudinal Estudantes, Adolescentes (n* = 2459)	Diminuição do uso recente de maconha entre estudantes de 13 a 15 anos. Não houve evidências de efeito em alunos de 11 a 12 anos.
Medeiros, et al., 2016 ^( 19 )^	#Tamojunto ( *Unplugged* )	Processo	Observacional, Longitudinal Estudantes, Professores, multiplicadores e gestores (n* = 84)	Os índices de implementação e monitoramento das aulas atingiram 94%. A duração das aulas foi insuficiente para cobrir todas as atividades propostas. Professores e alunos mostraram satisfação com o programa.
Schneider, et al., 2016 ^( 8 )^	ELOS ( *Good Behavior Game* )	Processo	Observacional, Longitudinal Professores, Equipe Pedagógica das Escolas, *Coaches* (n* = 43)	Alta aceitabilidade por professores e diretores, atribuída a sua estratégia de manejo de sala de aula. Houve indicações da necessidade de adaptações que reflitam o contexto social e econômico brasileiro.
Strelow, 2016 ^( 20 )^	ELOS ( *Good Behavior Game* )	Processo	Observacional, Transversal Estudantes, Adolescentes (n* = 27)	Crianças mostraram alta aceitabilidade do ELOS e expressaram a percepção de resultados de um aumento na colaboração com colegas e desejo de continuidade do jogo.
Lopes, 2016 ^( 14 )^	#Tamojunto ( *Unplugged* )	Processo	Observacional, Longitudinal Professores (n* = 53)	Os professores gradualmente passaram a se interessar mais pela prática de prevenção e pelo programa. Avaliou-se uma mudança no discurso, de uma abordagem moralista e estigmatizante para uma perspectiva mais compreensiva.
Medeiros, 2016 ^( 21 )^	#Tamojunto ( *Unplugged* )	Processo	Observacional, Longitudinal Estudantes, Adolescentes (n* = 1336)	Implementação em 94% das aulas, mas apenas 57% com fidelidade ao manual. Identificou-se exclusão de algumas atividades, dificuldades no planejamento pelos professores e falta de apoio dos administradores. Relataram melhorias no ambiente da sala de aula e nas habilidades pessoais.
Martins, 2016 ^( 22 )^	#Tamojunto ( *Unplugged* )	Resultado	Correlacional, Transversal Estudantes, Adolescentes (n* = 5007)	A análise do perfil da amostra para compreensão de possíveis moderadores de efeitos do programa identificou a relação entre estilos parentais e uso de drogas.
Menezes, 2016 ^( 12 )^	Famílias Fortes ( *Strengthening Families Program* )	Processo	Observacional, Transversal Profissionais da Saúde, Facilitadores, Multiplicadores do programa (n* = 41)	A adaptação cultural foi realizada com qualidade. Entretanto, o programa precisa ser adequado aos aspectos relacionados às desigualdades sociais e ao analfabetismo da população, o que implica em novas adaptações.
Sanchez, et al., 2017 ^( 23 )^	#Tamojunto ( *Unplugged* )	Resultado	Experimental (ECR ^§^ ), Longitudinal Estudantes, Adolescentes (n* = 4253)	Desfecho que melhorou: retardou o primeiro uso de inalantes. Desfecho que piorou: primeiro uso de álcool.
Peres, et al., 2017 ^( 24 )^	#Tamojunto ( *Unplugged* )	Processo	Observacional, Transversal Profissionais da saúde, profissionais da educação (n* = 18)	Percepção positiva dos profissionais sobre estratégias conjuntas para a implementação do programa e fortalecimento da conexão entre saúde e escola. Avaliou-se desafios no entendimento da intersetorialidade na prática.
Conegundes, 2017 ^( 25 )^	#Tamojunto ( *Unplugged* )	Resultado	Experimental (ECR ^§^ ), Longitudinal Estudantes, Adolescentes (n* = 6387)	Estudantes que participaram do grupo intervenção e que não moravam com a mãe aumentaram a chance do consumo de álcool quando comparado ao grupo controle.
Pedroso, 2017 ^( 26 )^	#Tamojunto ( *Unplugged* )	Processo	Observacional, Longitudinal Professores, Facilitadores, Multiplicadores (n* = 671)	Para sustentabilidade e disseminação do programa como política pública indica-se investir em uma mudança paradigmática da abordagem de drogas pelos implementadores, aumentar uso de metodologias interativas, adequar tempo da hora-aula e ampliar intersetorialidade .
Sanchez, et al., 2018 ^( 27 )^	#Tamojunto ( *Unplugged* )	Resultado	Experimental (ECR ^§^ ), Longitudinal Estudantes, Adolescentes (n* = 5007)	Resultados indicam que o programa retardou o uso de inalantes, mas piorou o primeiro uso de álcool entre os estudantes que participaram do programa.
Murta, et al., 2018 ^( 28 )^	Famílias Fortes ( *Strengthening Families Program* SFP 10-14-UK)	Processo	Observacional, Longitudinal Estudantes, Adolescentes, Pais, Facilitadores, Multiplicadores do Programa (n* = 33)	Programa percebido como culturalmente relevante e parcialmente claro. As adaptações culturais devem focar em aspectos linguísticos dos materiais e procedimentos, considerando diferenças culturais, econômicas e educacionais das famílias, conduzidas de forma participativa.
Medeiros, et al., 2018 ^( 29 )^	#Tamojunto ( *Unplugged* )	Processo	Observacional, Transversal Estudantes, Adolescentes, Professores, Equipe pedagógica das escolas (n* = 78)	As facilidades da implementação são suporte técnico, formação, supervisão e apoio dos administradores escolares. As dificuldades envolveram o tempo das aulas, a obtenção de materiais de apoio e o cumprimento do currículo regular. Como potencialidade, destacou-se melhorias na convivência.
Gusmões 2018 ^( 30 )^	#Tamojunto ( *Unplugged* )	Resultado	Experimental (ECR ^§^ ), Longitudinal Estudantes, Adolescentes (n* = 6637)	Reduziu o *bullying* nos primeiros nove meses, mas o efeito não se manteve após 21 meses. Fatores de risco para *bullying* incluíram envolvimento prévio em violência e uso de álcool e inalantes, mães com episódios de embriaguez.
D’Tôllis, 2018 ^( 31 )^	ELOS ( *Good Behavior Game* )	Processo	Observacional, Longitudinal Gestores nacionais (n* = 2)	A adaptação cultural foi avaliada como satisfatória. Os aspectos de planejamento de implementação, sensibilidade cultural, fidelidade ao programa original e processos avaliativos e de refinamento foram bem desenvolvidos.
Garcia, 2018 ^( 32 )^	ELOS ( *Good Behavior Game* )	Processo	Observacional, Longitudinal Professores, Equipe das Escolas, Facilitadores (n* = 60)	A avaliação da fidelidade de implementação foi positiva, mas os resultados foram parcialmente inconclusivos devido à um problema na devolução dos formulários de fidelidade por professores e multiplicadoras.
Abdala, 2018 ^( 33 )^	Famílias Fortes ( *Strengthening Families Program* )	Processo	Observacional, Transversal Facilitadores, Multiplicadores do Programa (n* = 26)	As barreiras apontadas pelos facilitadores para a implementação incluíram as condições de trabalho inadequadas, administração municipal frágil, metodologias de capacitação insuficientes e baixa adesão de profissionais.
Sanchez, et al., 2019 ^( 34 )^	#Tamojunto ( *Unplugged* )	Resultado	Experimental (ECR ^§^ ), Longitudinal Estudantes, Adolescentes (6391)	Os resultados sugerem que há falta de efeito do programa sobre as crenças normativas na relação com o uso de drogas.
Murta, et al., 2020 ^( 7 )^	Famílias Fortes ( *Strengthening Families Program* )	Resultado	Pré-Experimental, Longitudinal Estudantes, Adolescentes, Pais e Facilitadores, Multiplicadores do Programa (n* = 126)	Aumento na autoeficácia de aprendizagem e diminuição da ausência escolar sem permissão dos pais. Efeitos nulos sobre o consumo de álcool, episódios de *binge drinking* e comportamento antissocial no último mês.
Abdala, et al., 2020 ^( 35 )^	Famílias Fortes ( *Strengthening Families Program* )	Processo	Observacional, Transversal Facilitadores, Multiplicadores do Programa (n* = 26)	As barreiras apresentadas pelos facilitadores foram condições de trabalho, administração municipal fraca, infraestrutura precária, grupo inadequado, metodologias de capacitação de facilitadores, baixa adesão de gestores e a escassez de financiamento.
Valente, et al., 2020 ^( 36 )^	#Tamojunto ( *Unplugged* )	Resultado	Experimental (ECR ^§^ ), Longitudinal Estudantes, Adolescentes (n* = 6391)	Resultados mostraram redução na habilidade de tomada de decisão e aumento do uso de drogas e violência.
Valente, et al., 2020^( 37)^	#Tamojunto ( *Unplugged* )	Resultado	Experimental (ECR ^§^ ), Longitudinal Estudantes, Adolescentes (n* = 6391)	Não foi identificado o potencial “reforço” dos comportamentos parentais sobre o uso de drogas entre aqueles que receberam intervenção. Foi identificada a associação entre menor exigência parental e maior uso de drogas por adolescentes.
Menezes, et al., 2020 ^( 38 )^	Famílias Fortes ( *Strengthening Families Program* )	Processo	Observacional, Transversal Facilitadores, Multiplicadores do Programa (n* = 42)	Sobre a fidelidade, metade dos facilitadores relatou excluir itens dos procedimentos e 73,2% dos entrevistados relataram ter feito uma ou mais modificações ao aplicar o programa.
Murta, et al., 2020 ^( 39 )^	Famílias Fortes ( *Strengthening Families Program* SFP 10-14-UK)	Processo	Observacional, Longitudinal Estudantes, Adolescentes, Pais (n* = 74)	Bons níveis de fidelidade e envolvimento dos pais e níveis moderados de envolvimento dos adolescentes. A intervenção é relevante e os procedimentos aceitáveis, mas identificou-se necessidade de adaptações culturais para facilitar compreensão para famílias de baixa escolaridade.
Coelho e Monteiro, 2020 ^( 40 )^	Jogo da onda digital	Processo	Observacional, Transversal Estudantes, Adolescentes (n* = 12)	Percepção de melhoria da compreensão de temas relacionados ao uso de drogas. Os participantes valorizaram o enfoque interativo e lúdico do jogo, que se destacou em comparação com abordagens educativas tradicionais.
Valente, 2020 ^( 41 )^	#Tamojunto ( *Unplugged* )	Resultado	Experimental (ECR ^§^ ), Longitudinal Estudantes, Adolescentes (n* = 6391)	O programa altera as habilidades de tomada de decisão, mas na direção oposta à proposta pelo modelo teórico do programa. A intervenção não mudou o uso de drogas.
Amato et al., 2021 ^( 6 )^	Programa PERAE ^||^ (SHAHRP ^¶^ )	Processo	Observacional, Longitudinal Estudantes, Adolescentes (n* = 348)	A fidelidade de implementação chegou a 66,8%. O percentual de aulas completas implementadas variou de 62,5% a 87,5%. Os alunos e professores relataram boa aceitabilidade do programa.
Murta, et al., 2021 ^( 42 )^	Famílias Fortes ( *Strengthening Families Program* )	Processo	Observacional, Longitudinal Estudantes, Adolescentes, Pais, Facilitadores (n* = 410)	Avaliou-se que a satisfação/aceitabilidade resultou no aumento da viabilidade. O programa melhorou a coesão familiar, mas piorou a sobrecarga dos facilitadores.
Valente e Sanchez, 2021 ^( 43 )^	PROERD ^†^ (DARE ^‡^ *Keepin’ it REAL* )	Resultado	Experimental (ECR ^§^ ), Longitudinal Estudantes, Adolescentes, (n* = 4030)	Melhorou a experiência escolar, mas piorou a intenção de usar drogas, habilidades de tomada de decisão, habilidades de recusa à oferta de drogas.
Sanchez, et al., 2021 ^( 44 )^	#Tamojunto 2.0 ( *Unplugged* )	Resultado	Experimental (ECR ^§^ ), Longitudinal Estudantes, Adolescentes (n* =5208)	Reduziu as chances de os alunos do 8º ano iniciarem o uso de álcool. Não foram encontrados efeitos sobre qualquer outra iniciação de drogas ou prevalência de uso/mês.
Sanchez, et al., 2021 ^( 45 )^	PROERD ^†^ (DARE ^‡^ *Keepin’ it REAL* - kiR)	Resultado	Experimental (ECR ^§^ ), Longitudinal Estudantes, Adolescentes (n* = 4030)	Apresentou um efeito negativo, indicando que alunos do 7º ano que já praticavam *binge drinking* antes e frequentavam o programa tinham chances maiores de manter esse uso.
Cogo-Moreira, et al., 2021 ^( 46 )^	#Tamojunto ( *Unplugged* )	Resultado	Experimental (ECR ^§^ ), Longitudinal Estudantes, Adolescentes (n* = 6390)	Os resultados indicaram que o comportamento violento precoce prediz mais uso de drogas entre adolescentes e mostra a falta de evidências de que #Tamojunto mude a dinâmica entre o uso de drogas e *bullying.*
Pinheiro-Carozzo, et al., 2021 ^( 47 )^	Famílias Fortes ( *Strengthening Families Program* )	Resultado	Pré-Experimental, Longitudinal Estudantes, Adolescentes, Pais (n* = 361)	Afeta dimensões parentais de forma diferente dependendo do estilo parental inicial e pode ajudar a melhorar fragilidades no cumprimento de suas deveres parentais a curto prazo.
Ferreira Junior, 2021 ^( 48 )^	PROERD ^†^ (DARE ^‡^ )	Resultado	Experimental (ECR ^§^ ), Longitudinal Estudantes, Adolescentes (n* = 4045)	O uso de álcool foi associado a todas as classes de *bullying* . O baixo desempenho escolar também esteve fortemente associado ao *bullying* . Alunos negros/pardos tiveram 3,35 vezes mais chances de alta vitimização por *bullying.*
Mariano, et al., 2021 ^( 49 )^	ELOS 2.0 ( *Good Behavior Game* )	Resultado	Experimental (ECR ^§^ ), Longitudinal Não informa	Protocolo do estudo de efetividade
Machado, et al., 2021 ^( 50 )^	Educação Sobre Drogas e Habilidades Sociais	Processo	Observacional, Longitudinal Estudantes/Adolescentes (n* = 145)	A maioria dos estudantes aprovou o programa, favorecendo a possibilidade de continuidade nos anos seguintes.
Farias, 2021 ^( 51 )^	Famílias Fortes ( *Strengthening Families Program* )	Processo	Observacional, Transversal Gestores (n* = 7)	Variáveis relacionadas à capacidade organizacional, adaptação do programa, suporte político, avaliação do programa e parcerias foram citados como essenciais para a sustentabilidade do programa.
Gusmões et al., 2022 ^( 52 )^	PROERD ^†^ (DARE ^‡^ *Keepin’ it REAL* - kiR)	Resultado	Experimental, Longitudinal Estudantes, Adolescentes e Instrutores do PROERD (n* = 4030)	O nível de fidelidade à implementação não teve influência na capacidade do programa de reduzir o uso de drogas entre adolescentes. Porém, a análise qualitativa revelou adaptações feitas pelos instrutores policiais.
Melo, et al., 2022 ^( 53 )^	#Tamojunto 2.0 ( *Unplugged* )	Processo	Observacional, Longitudinal Professores e Diretores, Coordenadores Pedagógicos (n* = 21)	Baixa fidelidade de implementação, boa qualidade na aplicação do programa e alto absenteísmo dos estudantes.
Schneider, et al., 2022 ^( 54 )^	ELOS ( *Good Behavior Game* )	Resultado	Quasi-Experimental, Longitudinal Estudantes, Adolescentes (n* = 1731)	Diminui a agressividade e disruptividade entre meninos. Não teve o mesmo efeito em meninas.
Valente, et al., 2022 ^( 55 )^	PROERD ^†^ (DARE ^‡^ , *Keepin’ it REAL* - kiR)	Resultado	Experimental, Longitudinal Estudantes, Adolescentes (n* = 4300)	Não possui evidências dos efeitos de mudanças no padrão de uso de drogas ao comparar intervenção com controle em qualquer um dos currículos do PROERD direcionados a alunos 5ª e 7ª série.
Garcia-Cerde, et al., 2022 ^( 56 )^	#Tamojunto 2.0 ( *Unplugged* )	Resultado	Experimental, Longitudinal Estudantes, Adolescentes (n* = 5208)	Aumentou o conhecimento sobre drogas e as crenças negativas/não positivas sobre o álcool. Nenhuma evidência foi encontrada sobre o efeito do programa com a maconha.
Almeida, et al., 2023 ^( 57 )^	#Tamojunto 2.0 ( *Unplugged* )	Resultado	Experimental, Longitudinal Estudantes, Adolescentes (n* = 5208)	Fatores como: ser menina, idade avançada, uso prévio de drogas e maior presença de sintomas de saúde mental foram os principais preditores do consumo de drogas no acompanhamento.
Gusmões, et al., 2023 ^( 58 )^	PROERD ^†^ (DARE ^‡^ , *Keepin’ it REAL* - kiR)	Processo	Observacional, Transversal Policiais Militares (n* = 19)	A maioria dos instrutores adaptam o programa. Os principais motivos extrínsecos para as alterações foram os desafios advindos da realidade cultural e o desempenho escolar dos alunos e a falta de suporte da escola. Já o motivo intrínseco foi o desempenho dos instrutores.
Valente, et al., 2023 ^( 59 )^	#Tamojunto 2.0 ( *Unplugged* )	Resultado	Experimental, Longitudinal Estudantes, Adolescentes (n* = 5208)	Reduziu indiretamente o *bullying* ao diminuir o uso de álcool. Mas o programa não foi diretamente eficaz na redução da prevalência de *bullying* (vitimização e perpetração).
Valente e Sanchez, 2023 ^( 60 )^	PROERD ^†^ (DARE ^‡^ , *Keepin’ it REAL* - kiR)	Resultado	Experimental, Longitudinal Estudantes, Adolescentes (n* = 4030)	Não teve efeito nas habilidades de tomada de decisão, nas atitudes em relação às drogas, nas habilidades de recusa e nas habilidades de comunicação, conforme proposto pelo modelo teórico.
Garcia-Cerde, et al., 2023 ^( 61 )^	#Tamojunto 2.0 ( *Unplugged* )	Resultado	Experimental, Longitudinal Estudantes, Adolescentes (n* = 5208)	Reduziu indiretamente o uso de álcool ao longo da vida, aumentando as crenças negativas sobre o álcool. Embora tenha sido observado um efeito indireto na diminuição do consumo excessivo de álcool, o efeito direto na diminuição do consumo de álcool foi estatisticamente significativo.
Oliveira, et al., 2023 ^( 62 )^	Descolado	Processo	Observacional, Transversal Informantes-chave do Programa (n* = 6)	O programa é avaliável. Os resultados da avaliação auxiliaram na organização e descrição de forma lógica do programa, assim como, contribuíram para o aprimoramento da sua estruturação, indicando a necessidade de ajustes nos objetivos, atividades e recursos.
Ferreira Junior, et al., 2023 ^( 63 )^	PROERD ^†^ (DARE ^‡^ , *Keepin’ it REAL* - kiR)	Resultado	Experimental, Longitudinal Estudantes, Adolescentes (n* = 4030)	Associação entre desfechos de *bullying* , violência escolar, o padrão de uso de drogas e descritores socioeconômicos.
Schneider, et al., 2023 ^( 64 )^	ELOS ( *Good Behavior Game* )	Resultado	Pré-Experimental, Longitudinal Estudantes, Crianças (n* = 624)	As turmas que receberam o programa com maior fidelidade apresentaram resultados mais positivos na diminuição dos comportamentos alvos. Turmas com baixa fidelidade apresentaram resultados inócuos ou possivelmente iatrogênicos.

*n = Número de participantes; ^†^PROERD = Programa Educacional de Resistência às Drogas e à Violência; ^‡^DARE = *Drug Abuse Resistance Education*; ^§^ECR = Ensaio Clínico Randomizado; ^||^PERAE = Programa de Estímulo à Saúde e Redução de Riscos Associados ao Uso de Álcool Aplicado ao Ambiente Educacional; ^¶^SHAHRP = *The School Health and Alcohol Harm Reduction Project*

### Programas de Prevenção sobre uso de drogas no Brasil

#### Características dos programas e estratégias preventivas sobre uso de drogas no Brasil

Dos onze programas ou estratégias de prevenção ao uso de drogas que foram avaliados no Brasil e identificados nesta revisão, sete são programas de prevenção universal, dois são versões atualizadas de programas já avaliados anteriormente, um programa é de prevenção seletiva e dois se configuram como estratégias preventivas. O formato da entrega foi geralmente desenhado como aula ou como alguma estratégia pedagógica, a exemplo de oficina ou jogo, como é o caso do Programa ELOS e o Jogo da Onda. A duração média de implementação diária dos programas é de 45 a 50 minutos, correspondendo a uma hora-aula (variando de 30 minutos a 2h30 minutos).

Em relação à substância alvo da intervenção, há somente um programa que se dirige especificamente para o álcool, que é o Programa de Estímulo à Saúde e Redução de Riscos Associados ao Uso de Álcool Aplicado ao Ambiente Educacional (PERAE), adaptação cultural do SHAHRP. Todos os outros abordam o consumo de álcool e outras drogas, com destaque para tabaco, maconha, inalantes, cocaína e *crack.* Há ainda os que não mencionam substâncias psicoativas específicas. É o caso do Programa ELOS, por ser direcionado para crianças e não incidir diretamente sobre o uso álcool e outras drogas, apenas seus preventivos a médio e longo prazo. A seguir, apresenta-se uma síntese de cada programa estudado.

O Programa mais avaliado no Brasil é o #Tamojunto, adaptação cultural do europeu *Unplugged*, realizado em escolas com alunos do 8º ano do ensino fundamental. Foram identificados 19 estudos avaliativos, dos quais oito focaram na avaliação de processo, incluindo a aceitabilidade de diferentes atores, a viabilidade, a fidelidade, a análise da intersetorialidade e da sua inserção nas políticas públicas^(14,16-17,19,21,24,26,29)^. Em termos de avaliação de resultado, houve um estudo de eficácia, relacionado à implementação-piloto feita em 2014^(18)^ e dois estudos de efetividade desenvolvidos na implementação de 2016, um com seguimento de nove meses e outro de 21 meses^(23,27)^. Quatro estudos abordaram efeitos secundários, como a relação entre uso de drogas e *bullying*/violência escolar, estilos parentais e *binge drinking* ^(22,25,30,37)^. Três outros estudos avaliaram os mediadores dos efeitos do programa na implementação de 2016^(34,36,41)^.

Após os resultados negativos, o programa foi revisado entre 2018 e 2019, definido como #Tamojunto 2.0, que se aproximou do conteúdo original do #Tamojunto 2.0. Houve seis estudos avaliativos, sendo um de avaliação de processo, voltado para a fidelidade^(53)^, e um estudo de efetividade^(44)^ que mostrou que os estudantes expostos ao programa apresentaram menor probabilidade de iniciar o uso de álcool do que os do grupo controle. Também foram investigados os efeitos secundários na relação entre uso de drogas e *bullying* ^(59)^, dois estudos de análise de mediação que avaliaram quais mecanismos funcionaram para alcançar os efeitos do programa^(56,61)^ e um estudo da moderação da condição da saúde mental e padrão de consumo dos adolescentes participantes do projeto^(57)^.

Já sobre o Programa ELOS: Construindo Coletivos, uma adaptação cultural do programa norte-americano *Good Behavior Game (GBG)*, foram encontrados seis estudos avaliativos, quatro deles focados na avaliação de processo^(8,20,31-32)^. Um estudo de resultados temporais mediado pela fidelidade, relacionado à implementação de 2014^(64)^ e um estudo de eficácia da implementação de 2016, que apontou a diminuição da agressividade e disruptividade em meninos (mas não em meninas), corroborando com resultados internacionais do GBG^(54)^. O ELOS 2.0 foi resultante de uma modificação de alguns procedimentos da primeira versão do ELOS, tendo sido localizado um registro do protocolo de um estudo de efetividade^(49)^.

O programa Famílias Fortes é uma adaptação cultural do *Strengthening Families Program* (SFP), de origem inglesa. Foram encontrados nove estudos avaliativos, sendo um estudo de levantamento de necessidade para adaptação cultural^(28)^, uma tese sobre o processo da adaptação cultural^(12)^ e um estudo sobre adaptações locais para o Nordeste^(38)^. Quatro estudos avaliaram o processo de implementação^(35,39,42,51)^. Dois estudos de resultados temporais mostraram o aumento na autoeficácia da aprendizagem escolar, mas efeito nulo para mudança no consumo de álcool e drogas nos adolescentes^(7)^. Por outro lado, houve melhora na relação entre pais e filhos e nos estilos parentais^(47)^.

O PROERD é a atividade preventiva mais implementada em escolas do país, estando presente em todas as regiões brasileiras. Foram encontrados nove estudos, sendo o primeiro deles um estudo quasi-experimental de 2014, quando o PROERD ainda seguia o modelo do programa DARE, dos Estados Unidos da América sem encontrar efeitos significativos nos resultados alvo^(15)^. Três estudos realizados em 2020-2021 analisaram resultados sobre a efetividade do PROERD, já sob o modelo do *Keepin’it REAL,* que indicaram não possuir evidências de efeitos na mudança do padrão de uso de drogas, tendo piorado o uso em forma de *binge drinking* ^(43,45,55)^. Foram realizadas análises dos efeitos sobre o *bullying* ^(48,63)^ e em análises de mediação, encontrou-se piora nas habilidades de comunicação, habilidades de tomada de decisão, atitudes em relação às drogas e habilidades de recusa^(60)^. Estudos também avaliaram o processo de implementação do PROERD para compreender as razões para os resultados inócuos ou potencialmente iatrogênicos^(52,58)^.

O PERAE, adaptação cultural do australiano SHAHRP, foi avaliado em um único estudo sobre viabilidade do programa para a realidade brasileira, com achados positivos^(6)^. É um programa australiano, com base em redução de danos para álcool em jovens, que mostrou evidências em seu país.

O Descolado, criado em 2018 pela Prefeitura de Recife em parceria com a Universidade Federal de Pernambuco (UFPE), é um programa de prevenção universal ao uso de álcool e outras drogas, baseado em habilidades de vida e no método inspirado em Paulo Freire, implementado nas escolas do Recife. O estudo analisado discute a avaliabilidade do Descolado^(62)^.

O Jogo da Onda Digital, uma estratégia educativa sobre drogas, almeja contribuir para a educação preventiva pelo Ensino de Ciências e Biologia. Originalmente desenvolvido em 2003^(65)^, foi adaptado à modalidade digital em 2019. O artigo encontrado fez uma avaliação de processo do jogo digital^(40)^.

O Programa M (M, de mulheres) é uma intervenção dirigida a mulheres jovens de comunidades de baixa renda na cidade do Rio de Janeiro, focado em mudança de atitudes, autoeficácia, conhecimento sobre vários comportamentos de risco para a saúde dessas mulheres, incluindo o uso de álcool e outras drogas. O programa foi analisado em uma tese de doutorado defendida na *Harvard University*, por meio de uma avaliação de eficácia^(13)^.

A estratégia Educação Sobre Drogas e Habilidades Sociais é uma intervenção realizada em escolas públicas estaduais no interior do estado do Espírito Santo, com 10 encontros temáticos sobre álcool e outras drogas e habilidades sociais em aulas de educação física. O estudo realizou uma avaliação de processo^(50)^.

A Figura 3 apresenta a descrição detalhada de todos os programas preventivos ao uso de drogas identificados na revisão.

**Figura 3 -:** Descrição dos Programas de prevenção ao uso de drogas avaliados no Brasil, segundo nome do programa, tipo de avaliação e de categoria, modelo teórico, intervenção e desfecho. Florianópolis, SC e São Paulo, SP, Brasil, 2024

**Nome do Programa**	**Tipo de Avaliação**	**Tipo e Categoria**	**Modelo Teórico**	**Intervenção**	**Desfecho-Alvo**
#Tamojunto	Processo (n*=9) Resultado (n*=10)	Programa de Prevenção Universal	Teoria aprendizagem social, habilidades de vida, modelo de crença em saúde e teoria de ação-atitude crítica	Aulas / 45-60 min. / 12 encontros/ realizadas por professores treinados, em escolas públicas, com apoio de facilitadores	Retardar o início e diminuir o padrão de uso de álcool, tabaco, maconha, inalantes, cocaína e *crack*
#Tamojunto 2.0	Processo (n*=1) Resultado (n*=5)	Programa de Prevenção Universal	Teoria aprendizagem social, habilidades de vida, modelo de crença em saúde e teoria de ação-atitude crítica	Aulas / 45-60 min. / 12 encontros/ realizadas por professores treinados, em escolas públicas, com apoio de facilitadores	Retardar o início e diminuir o padrão de uso de álcool, tabaco, maconha, inalantes, cocaína e *crack*
Famílias Fortes	Processo (n*=8) Resultado (n*=2)	Programa de Prevenção Universal	Teoria dos sistemas familiares, teoria da cognição social, modelo de resiliência e modelos socioecológicos	Oficinas / 2h a 2h e 30 min. / 7 a 11 encontros, com pais e adolescentes (10-14 anos), realizado nos CRAS ^†^ , por profissionais do SUAS ^‡^	Diminuir padrão de uso de álcool e outras drogas de adolescentes e modificar padrões de relacionamento entre pais e filhos e estilos parentais
ELOS: Construindo Coletivos	Processo (n*=5) Resultado (n*=1)	Programa de Prevenção Universal	Treinamento de habilidade de vida, com base na análise experimental do comportamento	Estratégia Pedagógica / 30 min. / frequência variada, geralmente três vezes na semana	Modificar comportamentos agressivos e disruptivos e fortalecer concentração na tarefa e colaboração entre pares em crianças.
ELOS 2.0	Resultado (n*=1)	Programa de Prevenção Universal	Treinamento de habilidade de vida, com base na análise experimental do comportamento	Estratégia Pedagógica / 30 min. / frequência variada, geralmente três vezes na semana	Modificar comportamentos agressivos e disruptivos e fortalecer concentração na tarefa e colaboração entre pares em crianças.
PROERD ^§^	Processo (n*=2) Resultado (n*=7)	Programa de Prevenção Universal	Teoria de Aprendizagem Socioemocional, Habilidades sociais e modelo de influência social da educação sobre drogas	Aulas / 50 min. / 10 encontros	Modificar o padrão do uso de álcool e outras drogas
Jogo da onda digital	Resultado (n*=1)	Estratégia de Prevenção Universal	Estratégia de psicoeducação e de crenças normativas sobre uso de drogas	Jogo / 2h / 1 encontro ou mais	Modificar o padrão do uso de álcool e outras drogas
PERAE ^||^	Processo (n*=1)	Programa de Prevenção Universal	Aprendizagem Socioemocional e Redução de Danos	Aulas / 1h / 8 encontros	Modificar o padrão do uso de álcool
Programa M (M, de mulheres)	Resultado (n*=1)	Programa de Prevenção Seletiva	Autoeficácia de Bandura, Mudança de atitudes, Conhecimento Crítico e percepção sobre comportamentos de risco	Manual e discussões em grupo: exposição à vídeo, campanha de comunicação social / 18 encontros	Fortalecer autoeficácia em relações interpessoais de mulheres com impactos para o uso álcool e outras drogas
Descolado	Avaliabilidade (n*=1)	Programa de Prevenção Universal	Pedagogia Paulo Freire, Habilidades de Vida	Aulas em escolas por professores, sobre uso de drogas, protagonismo juvenil e Plano de Promoção Pessoal (PPP) do estudante	Modificar o padrão do uso de álcool e outras drogas
Educação Sobre Drogas e Habilidades Sociais	Processo (n*=1)	Estratégia de Prevenção Universal	Psicoeducação	10 encontros temáticos sobre álcool e outras drogas e habilidades sociais em aulas de Educação Física.	Modificar o padrão do uso de álcool e outras drogas

*n = Número de estudos; ^†^CRAS = Centro de Referência de Assistência Social; ^‡^SUAS = Sistema Único de Assistência Social; ^§^PROERD = Programa Educacional de Resistência às Drogas e à Violência; ^||^PERAE = Programa de Estímulo à Saúde e Redução de Riscos Associados ao Uso de Álcool Aplicado ao Ambiente Educacional

## Discussão

Poder-se-ia discutir que onze é ainda um número baixo de estratégias ou programas preventivos ao uso de drogas avaliados no país, sendo que mais da metade não tem estudos de eficácia e efetividade para ajudar a compor um cardápio mais robusto de ações preventivas baseadas em evidência. No entanto, é importante destacar que já existem programas consistentemente consolidados em suas evidências, direcionados para públicos em diferentes fases do ciclo vital (crianças, adolescentes e famílias), o que é um ponto de partida importante para sustentar a implementação de políticas públicas.

Porém, os cenários de intervenção das estratégias e programas encontrados ainda são restritos, sendo a sua grande maioria em escolas públicas ou nos Centros de Referência em Assistência Social (CRAS). Nesse sentido, seria interessante investir no desenvolvimento ou adaptação de programas ou sistemas baseados na comunidade. Na medida em que a prevenção comunitária envolve o protagonismo e corresponsabilização de lideranças locais, a produção de dados sobre necessidades específicas de cada território é considerada um dos âmbitos de atuação mais significativos por incidir sobre valores locais e trazer o sentido de pertencimento para os participantes^(65)^. Da mesma forma, é fundamental investir mais na prevenção ambiental, considerada o âmbito mais eficaz da prevenção. Essa abordagem atua de forma ampla, abrangendo o cenário social e com objetivos de redução da disponibilidade de drogas, mudança de normas sociais e o controle da publicidade e do marketing, entre outros. Um exemplo bem-sucedido dessa estratégia foi a prevenção ao tabaco, iniciada em 1986^(65)^.

Na revisão aqui discutida, apareceram avaliações que foram categorizadas como estratégias e programas preventivos, sendo que a ciência da prevenção propõe a existência de diferentes níveis de organização das ações preventivas, distinguindo entre elas. As estratégias são planos ou abordagens que têm uma sustentação teórica e sofreram um processo de modelagem metodológica e de intervenção visando enfrentar o desfecho que tem como alvo de forma mais estruturada. Podem envolver a integração de várias ações coordenadas e orientadas por objetivos a médio ou longo prazo. As estratégias podem ser mais flexíveis em seu uso, ainda que devam ser avaliadas e procurar sustentar-se em evidências^(65)^. Já os programas preventivos são bem mais sistematizados do que as estratégias, pois devem ter um modelo lógico e/ou uma teoria da mudança bem estruturados, ter bem definidos os seus *inputs* (recursos, esforços e insumos necessários para implementar uma atividade), seus objetivos, os *outputs* (resultados imediatos) e *outcomes* esperados (resultados almejados, a médio e longo prazo), e os indicadores de sua avaliação. Além disso, os programas devem ser, de preferência, manualizados, ou seja, ter orientações estruturadas e objetivas para os implementadores (os que fazem o suporte técnico e supervisão) e para os participantes, a fim de garantir a fidelidade de sua implementação. Os programas também têm de ser sustentados em evidências. Todos os dois níveis devem organizar a formação para quem vai realizá-los, visando garantir a qualidade da sua implementação e construir propostas de avaliação de processo e de resultados^(5,65)^.

A classificação de ações preventivas conforme os tipos de prevenção é um importante analisador para a tomada de decisão sobre qual programa implementar, conforme objetivos direcionados ao nível de risco que a população alvo apresenta. Com isso, a prevenção universal é assim chamada por ser dirigida à população em geral, sem qualquer estratificação de fatores de risco. São qualificados nesse tipo de prevenção os programas quando, por exemplo, implementados nas escolas, são direcionados a todos os alunos de uma determinada sala de aula, sem haver uma seleção específica de estudantes com maior vulnerabilidade para o uso de álcool e outras drogas (AD). Já a prevenção seletiva é voltada para populações já com alguma condição de risco para o uso problemático em AD, o que não significa que essas pessoas já consumam substâncias psicoativas, mas sim, que a ação se direciona para aquelas com mais risco de fazê-lo. Por exemplo, programas realizados com filhos de usuários em recuperação ou implementados em escolas localizadas em regiões de alta vulnerabilidade, onde há tráfico de droga fortemente presente. E, por fim, a prevenção indicada, destinada a pessoas já identificadas com alto risco para consumo AD, em relação às quais já há a constatação de problemas relacionados ao consumo de substâncias psicoativas, que visam minimizar o consumo, reduzir danos e melhorar a qualidade de vida, visando promover saúde e reinserção social^(5,65)^. Apareceu na revisão um predomínio de estratégias e programas do tipo de prevenção universal, sendo necessário refletir sobre tais tendências e questionar sobre a possibilidade de diversificar o escopo das ações nas políticas públicas brasileiras.

O #Tamojunto, em sua primeira e segunda versões, foi o programa mais avaliado, com ciclos de estudos de eficácia, efetividade, fidelidade, discutindo aspectos que atuaram para produzir ou não os resultados almejados, seja quando teve resultados iatrogênicos, exigindo sua readequação, seja quando, na segunda versão, alcançou os resultados esperados ao retardar o início do uso de álcool, assim como efeitos secundários sobre a prática de *bullying*. Os resultados iatrogênicos desafiaram análises sobre aspectos do processo de adaptação cultural e da qualidade do processo de implementação^(23,27)^, sendo que “por vias tortas”, contribuiu para o crescimento da ciência da prevenção brasileira, na medida em que exigiu que uma rede de pesquisadores analisasse e aprendesse com erros, reforçando a necessidade de monitoramento da implementação e de adequações na sensibilidade cultural.

Vários estudos do #Tamojunto, seja na primeira ou segunda versão, dialogaram com exigências atuais de estudos avançados em ciência da prevenção^(66)^, como as análises de efeitos secundários, mediação e moderação deste programa. Pelos estudos de mediação buscaram conhecer e analisar os mecanismos do modelo lógico do programa que atuaram de fato no alcance dos resultados obtidos, sendo importantes análises para redimensionar o próprio programa ou para o planejamento da implementação em larga escala^(34,36,41,56,61)^. Pelas análises de moderação analisaram elementos contextuais interferiram no resultado, como fatores como qualidade da implementação, condições psicossociais dos estudantes, dentre outros^(57)^. As análises mais robustas auxiliam na compreensão de como os programas funcionam, auxiliando na consolidação das evidências e na tomada de decisões^(5,66)^.

O ELOS, em suas duas versões, trouxe resultados de eficácia e efetividade, com mudanças nos comportamentos agressivos, disruptivos e no engajamento em tarefas escolares, como resultados importantes na diminuição de vulnerabilidades pessoais das crianças, o que antecipa a possibilidade de melhoria nas habilidades de vida para o futuro de sua trajetória existencial. Seus resultados dialogaram com os de outros países e mostraram evidências de desenvolver habilidades de vida em crianças e diminuir vulnerabilidades e comportamentos antissociais, podendo ser uma ótima ação para ser utilizada com crianças, em especial meninos, pensando em uma estratégia de promoção de saúde mental para um futuro com jovens com melhor autoestima e menor riscos psicossociais^(54)^. O estudo feito sobre a moderação da fidelidade de implementação sobre os resultados temporais demostrou que o cuidado na implementação é fundamental para obter resultados almejados^(64)^, corroborando aquilo que é pressuposto na ciência da implementação e corroborando, novamente, a necessidade de monitoramento das ações preventivas^(65)^.

O Famílias Fortes, da mesma forma, em seu estudo de resultados temporais ou de efetividade, produziu melhoria na autoeficácia escolar, o que já pode ser considerado um efeito secundário importante^(7)^. Também houve melhoras nos relacionamentos entre pais e filhos e nas habilidades parentais e na modificação de estilos parentais negligentes e autoritários, o que é um resultado muito importante para o fortalecimento dos vínculos familiares^(47)^, ainda que não tenham aparecido resultados diretos sobre a mudança do padrão do uso de álcool dos adolescentes. Sendo assim, o Famílias Fortes necessita mais pesquisa para ter suas evidências comprovadas.

Da mesma forma, foi importante ter o conhecimento de que o programa preventivo mais largamente implementado nas escolas brasileiras, envolvendo praticamente todo o território nacional, o PROERD, mostrou-se inócuo na maioria dos desfechos alvos ou até iatrogênico^(15,43,45,55)^. Em sua avaliação em 2014, ainda sob o modelo DARE, ou na avaliação de 2020, já sob o modelo *Keepin’it Real*, ele não mostrou efeitos para retardar a idade do uso inicial de álcool, não modificou o padrão de uso de álcool e outras drogas e foi iatrogênico para o padrão *binge drinking* entre os adolescentes. Além disso, foi também inócuo para a prática ou vitimização do *bullying* ^(48,63)^. Esta constatação deve servir para alertar governos municipais, estaduais e gestores de escolas públicas e privadas sobre a necessidade de solicitarem a remodelação do programa.

Destacamos, com isso, o mérito de estudos que apontaram resultados negativos, ou seja, avaliações robustas que mostram efeitos inócuos ou iatrogênicos dos programas preventivos ao uso de drogas, a fim de indicar a necessidade de mudanças nos conteúdos ou metodologia dos programas e de sugerir alterações no uso destes em aplicações locais ou em larga escala. A literatura mostra a importância de produzir reflexões e publicar esses tipos de resultados não esperados, a fim de subsidiar desenvolvedores de programas e gestores de políticas públicas na melhoria de suas ações, devendo-se considerar as complexidades envolvidas nestes resultados negativos e reconhecer a multiplicidade de consequências intencionais e não intencionais ao planejar ou avaliar intervenções para prevenir o uso de drogas^(67)^.

Em suma, constatou-se que existem programas que já apresentam avaliações bem elaboradas de processo, mas ainda não realizaram avaliações de resultado, o que aponta para a necessidade de promover uma nova série de estudos para verificar sua eficácia e efetividade. Nesse sentido, as políticas públicas preventivas enfrentam reptos importantes, como a continuidade das avaliações já iniciadas para alguns dos programas mencionados, além de incentivar o desenvolvimento de novos programas e sistemas preventivos brasileiros.

Entretanto, um dos desafios do desenvolvimento de programas novos é o de seguir criteriosamente as etapas do ciclo da pesquisa em prevenção, que exigem tempo, dedicação de especialistas e financiamento alto, na medida em que implicar o detalhamento das etapas e processos para produzir evidências, iniciando com o levantamento de necessidades, desenvolvimento da intervenção e construção do seu modelo lógico e de sua avaliabilidade, teste-piloto, avaliação de eficácia, avaliação de efetividade, avaliação do processo de implementação, estudos de difusão e de adaptação cultural para outros contextos^(31-32,65)^. Outra exigência importante para o desenvolvimento de intervenções preventivas no Brasil é construir processos participativos e promotores de inclusão, nos quais “a prevenção deixe de ser mais um mecanismo de controle e perseguição de pessoas e que amplie sua perspectiva para ações baseadas na integralidade do cuidado e para a dimensão da ética em seu sentido pleno”^(5)^. Nessa direção eles devem estar alinhados com teorias e metodologias coerentes com os princípios que regem o Sistema Único de Saúde, o Sistema Único de Assistência Social, a Rede de Educação, a Segurança Cidadã, a Rede de Cultura, entre outros setores, a fim de promover a integração das políticas e desenvolver a intersetorialidade^(5,24)^.

A relevância desta revisão passa por destacar as características metodológicas e os resultados obtidos dos estudos avaliativos de estratégias e programas para uso de álcool e outras drogas no Brasil, visando contribuir para a construção de um cardápio de ações preventivas com evidências, necessário para dar sustentação à implementação de sistemas preventivos e de políticas públicas neste campo. Desse modo, o Brasil poderia começar a consolidar o desenvolvimento de uma plataforma nacional para certificação de estratégias e programas preventivos, fundamentada em padrões científicos claros, que facilitaria o acesso a informações sobre ações preventivas validadas para gestores, profissionais e cientistas interessados. Esse modelo já é adotado em outros países, como o *Blueprints for Healthy Youth Development* ^(68)^ nos Estados Unidos, o *Xchange Prevention Registry* ^(69)^ do *European Union Drugs Agency*, e a Evidência Viva^(70)^, recentemente criada pela *European Union Drugs Agency*, em parceria com o UNODC e governo brasileiro, para compilar programas da América Latina. Além disso, destacam-se alguns *sites* desenvolvidos em países latinos, como o *Mi Brújula* ^(71)^, da Fundación San Carlos de Maipo do Chile, e o *Guía de Programas Preventivos del Consumo de Sustancias Psicoativas en Colombia* ^(72)^, da *Corporación Nuevos Rumblos*.

Vislumbra-se, com isto, a importância do investimento público por parte de um governo no desenvolvimento da ciência de um país, especialmente na área de prevenção. No contexto da produção científica relacionada aos achados que subsidiam políticas públicas, é interessante analisar o investimento de regiões no modelo I+D, que implica a clareza da relação entre investigação e desenvolvimento do país^(73)^. Esse investimento promove o avanço de uma área de produção de conhecimento e apoia a implementação em larga escala de tecnologias sociais baseadas em evidências, trazendo benefícios significativos para a promoção da saúde da população^(5)^. Os principais programas ao uso de álcool e outras drogas analisados e atualmente com indicadores de eficácia e efetividade em nosso país foram fruto desses incentivos públicos.

O momento de investimento na ciência da prevenção que Governo Federal realizou entre 2013 e 2018 movimentou o cenário acadêmico, de governança e de implementadores de políticas públicas^(5)^, resultando na criação da Associação Brasileira de Prevenção e Promoção da Saúde (BRAPEP), que se constituiu para articular pesquisadores e profissionais que advogavam por melhorias na área, tanto na investigação e avaliação quanto na implementação de políticas públicas, com a pauta da expansão e qualificação da ciência preventiva no país^(74)^.

Nesse sentido, destaca-se o fortalecimento da relação entre os formuladores e gestores de políticas públicas e a academia e pesquisadores, estimulada nos governos do Presidente Lula e Dilma, ao compreenderem que a “sistematização do conhecimento e sensibilidade para a questão social brasileira não são antagônicos. A ciência pode e deve ser mais um instrumento de transformação de grupos e países”^(5)^. O Brasil veio, assim, pouco a pouco, destacando-se por sua produtividade acadêmica no campo da prevenção ao uso de álcool e outras drogas, como analisado, por exemplo, em pesquisa anterior, de caráter bibliométrico, sobre as redes de colaboração entre países latino-americanos e da União Europeia em produções científicas no campo AD, entre 2001 e 2011, a qual mostrou um aumento gradativo da colaboração científica entre esses dois blocos de países, sendo que sobressaiu a produtividade feita pelo Brasil dentre os países da América Latina^(73)^.

Da mesma forma, o *site* Evidência Viva, que compila evidências de programas da América Latina para estabelecer recomendações, ao destacar seis programas com processos mais robustos de avaliação – sendo três recomendados (*Unplugged*/#Tamojunto2.0, *Keepin’ it REAL*, *Good Behavior Game*/ELOS), um que necessita de mais evidências (*Strengthening Families Program*/Família Fortes) e dois não recomendados (*Unplugged*/#Tamojunto, PROERD/D.A.R.E-kiR) – utilizou evidências produzidas por estudos aqui analisados. Portanto, dos programas analisados, cinco deles tiveram seus estudos realizados no Brasil^(70)^.

Por fim, algumas limitações deste estudo podem ser apontadas, entre elas a questão do “silêncio” nas buscas, ou seja, quando algumas publicações relacionadas ao objetivo não aparecem nas pesquisas, mesmo com o cuidado da elaboração da equação de busca. Buscou-se suprir tais lacunas com a consulta a especialistas na área. Foi também uma limitação não ter realizado uma análise sistemática de referências dos artigos encontrados, o que pode ter implicado que publicações podem ter escapado de nosso radar. Outra dificuldade de acesso foi quanto ao banco de teses e dissertações: nem todos os catálogos contêm dados de todos os programas de pós-graduação, o que pode implicar que muitos trabalhos relacionados ao tema podem não ter sido incluídos. Optou-se, em alguns casos, por teses e dissertações realizadas na modalidade de artigos, os quais foram encontrados já publicados, por não incluir o trabalho acadêmico, para evitar algumas repetições.

## Conclusão

O Brasil, com apoio em investimentos públicos, avançou para superar a falta de evidências no campo da prevenção ao uso de álcool e outras drogas, já possui um conjunto de experiências bem-sucedidas em avaliação de estratégias e programas preventivos, com alguns programas já com evidências robustas disponíveis para as políticas públicas e interessados em prevenção ao uso de álcool e outras drogas. As autorias e colaborações nas publicações analisadas, bem como a criação de associações de pesquisa evidenciam a consolidação gradual de uma rede de pesquisadores e especialistas em prevenção. Tal rede possui o potencial de contribuir significativamente para o desenvolvimento de políticas públicas baseadas em evidências, além de fortalecer a produção contínua de conhecimento por meio da parceria entre academia e governos.

A maioria dos programas avaliados nesta revisão é de caráter universal, direcionados a adolescentes, desenvolvidos em escolas e focados em várias substâncias. Portanto, é necessário ampliar o escopo dos programas, para incluir a prevenção baseada na comunidade e a prevenção ambiental. Da mesma forma, faz-se necessário direcionar esforços para novos grupos populacionais, como jovens e adultos jovens, que é uma faixa etária com alto risco de envolvimento prejudicial no uso de psicoativos; tanto quanto as populações indígenas, que têm tido uma relação bastante prejudicial ao ter contato com o álcool e as drogas da sociedade de consumo ocidental. O desenvolvimento de programas seletivos e indicados também deve ser considerado no planejamento de projetos governamentais, incluindo populações de médio e alto risco para o consumo de substâncias psicoativas. Esse cenário aponta para a importância de consolidar uma ciência da prevenção no Brasil que seja sustentada em evidências, culturalmente sensível e sustentável, contribuindo para a formulação de políticas públicas mais estruturadas e impactantes.

# Translation: Características metodológicas de estudios evaluativos de programas preventivos para drogas en Brasil: revisión de alcance ^*^

**DOI:** 10.1590/1518-8345.7709.4577

**Authors:** Daniela Ribeiro Schneider, Charlene Fernanda Thurow, Tallita Franzoloso, Leila Gracieli da Silva, Guilherme Gomes Silva, Elaine Lucas dos Santos, Liz Paola Domingues, Leila Pimentel dos Anjos, Fernanda Machado Lopes, Ana Regina Noto

**Affiliations:** 1 Universidade Federal de Santa Catarina, Departamento de Psicologia, Florianópolis, SC, Brasil.; 2 Becaria (o) del Conselho Nacional de Desenvolvimento Científico e Tecnológico (CNPq), Brasil.; 3 Becaria de la Coordenação de Aperfeiçoamento de Pessoal de Nível Superior (CAPES), Brasil.; 4 Universidade Estadual do Norte do Paraná, Departamento de Enfermagem, Jacarezinho, PR, Brasil.; 5 Universidade Federal de São Paulo, Departamento de Psicobiologia, São Paulo, SP, Brasil.

**Keywords:** Programas, Prevención, Revisión, Abuso de drogas, Brasil, Política Pública

## Abstract

mapear estudios que desarrollaron procesos evaluativos de programas de prevención para el uso de alcohol y otras drogas en Brasil, analizando sus características metodológicas.

revisión de alcance orientada por el *Joanna Briggs Institute Manual* y por el *Preferred Reporting Items for Systematic Reviews and Meta-Analyses extension for Scoping Reviews*. Se realizaron búsquedas en ocho bases de datos y un banco de tesis y disertaciones, seleccionados a doble ciego en *Rayyan*®, analizados con enfoque en la metodología de los estudios evaluativos y mediante síntesis cualitativa.

de las 56 publicaciones incluidas, la mayoría utilizó diseño observacional, generalmente relacionado con las evaluaciones de proceso (n = 26), seguido por el experimental (n = 23) y cuasiexperimental (n = 8), en los análisis de resultados en términos de efectividad y eficacia, respectivamente. De las once acciones analizadas, ocho son programas de prevención universal, uno de prevención selectiva y dos se configuran como estrategia preventiva.

la mayoría de los programas evaluados es de carácter universal, dirigidos a adolescentes, realizados en escuelas y enfocados en varias drogas. Se recomienda enfocar en nuevos grupos, como en poblaciones indígenas y en otras condiciones de riesgo, además del desarrollo de programas selectivos en futuras iniciativas gubernamentales para prevenir el uso de drogas en Brasil.

## Destacados

**(1)** Se localizaron nueve programas preventivos de drogas con procesos de evaluación en Brasil. **(2)** De los nueve programas, ocho son de prevención universal y uno de prevención selectiva. **(3)** Algunos programas presentan evidencias consistentes para respaldar políticas públicas. **(4)** El PROERD no demostró efectividad en la prevención, requiriendo una remodelación. **(5)** Existe la necesidad de ampliar el alcance de los programas preventivos con evidencias en Brasil.

## Introducción

Desde la década de 1970, la prevención se ha consolidado gradualmente como una ciencia fundamentada en bases teóricas consistentes y en el rigor metodológico. La construcción de evidencias se ha vuelto esencial para asegurar la eficacia de las acciones preventivas y el alcance de los resultados esperados, con el objetivo de formular políticas públicas que prioricen la calidad, la rentabilidad y la promoción de la salud. En este contexto, es indispensable que los países adopten esta perspectiva científica para garantizar la calidad de sus políticas^(1)^.

En Brasil, aunque se realizan muchas acciones preventivas, la evaluación para comprobar la eficacia de estas iniciativas no es tan común. Este hecho, sumado al carácter puntual de las intervenciones, dificulta que el área promueva sus evidencias de manera eficaz y muestra que el proceso de consolidación de la ciencia de la prevención en el país aún está en una etapa emergente^(1)^. Uno de los primeros estudios de revisión sobre programas preventivos para el uso de drogas en Brasil recopiló investigaciones publicadas entre los años 1991 y 2001 y concluyó que las acciones de prevención en la década de 1990 fueron impulsadas por el modelo de prevención adoptado para enfrentar los desafíos de las Enfermedades de Transmisión Sexual^(2)^. Estas acciones recibieron críticas por su carácter reduccionista, centrado únicamente en la enfermedad, siguiendo la lógica de la “guerra contra las drogas” que predominaba en este campo.

En este contexto, se comenzó a ampliar la mirada hacia la relación entre las vulnerabilidades personales y contextuales, y surgieron los primeros proyectos sustentados en la lógica de la reducción de daños. La producción científica aún estaba muy enfocada en los aspectos farmacológicos de la droga en sí y en el tratamiento de la dependencia. La mayoría de los proyectos preventivos analizados tenía por objetivo alcanzar la escuela, influenciados por especialistas que defendían el espacio escolar como privilegiado para el desarrollo de este tipo de intervención. Prevalecía el desarrollo de proyectos piloto en escuelas públicas monitoreadas por el Ministerio de Salud, que aún utilizaban metodologías tradicionales, sustentadas en conferencias y transmisión de información, realizadas principalmente por agentes extraescolares, como médicos y policías. En esta revisión, realizada hasta el año 2001, no aparecieron estudios de eficacia y efectividad de los programas analizados^(2)^.

En una revisión sistemática posterior, con artículos publicados hasta el año 2012, se analizaron publicaciones de evaluación de programas en salud mental en Brasil, incluyendo problemas de comportamiento, violencia, abuso sexual, abuso de alcohol y drogas, y vacío existencial^(3)^. Se encontró una predominancia de estudios preexperimentales y cuasiexperimentales, sin evaluaciones de seguimiento y con muestras pequeñas. Gran parte de las evaluaciones eran de proceso o de levantamiento de necesidades, o bien pruebas piloto de programas, siendo escasos los estudios para evaluación de eficacia, efectividad y difusión^(3)^. Hasta aquí, se observa cierta evolución de la ciencia de la prevención en el escenario brasileño, pero aún incipiente.

Otro estudio más reciente, publicado en 2020, desarrollado con 1151 directores de escuelas, sobre acciones y programas preventivos realizados en escuelas públicas y privadas brasileñas, constató que los programas aún se implementan sin regularidad y con corta duración, dirigidos principalmente a los alumnos, utilizando diferentes modelos teóricos y promovidos principalmente por la Policía Militar^(4)^. De este modo, más de tres décadas de investigaciones y revisiones demuestran la evolución gradual de la ciencia de la prevención brasileña, que aún enfrenta muchas dificultades y necesita más incentivos para su desarrollo.

El año 2013 fue un hito para el fortalecimiento de la ciencia de la prevención brasileña, debido a una importante iniciativa del Gobierno Federal que impulsó las principales investigaciones e implementaciones de programas basados en evidencia en el país, contribuyendo a cambiar el escenario de la ciencia de la prevención en Brasil. La acción fue realizada en asociación entre la *Coordenação de Saúde Mental*, Álcool *e Outras Drogas*, del *Ministério da Saúde*, con la Oficina de las Naciones Unidas contra la Droga y el Delito (UNODC), la *Secretaria Nacional de Políticas sobre Drogas* (SENAD), del *Ministério da Justiça*, y la *Fundação Oswaldo Cruz*. Tres programas fueron sometidos a procesos de adaptación cultural en Brasil: ELOS: *Construindo Coletivos* (ELOS) (del original en inglés *Good Behavior Game*), #*tamojunto* (*Unplugged*) y *Fortalecendo Familias* (*Strengthening Families Programme*), buscando abarcar acciones preventivas a lo largo del ciclo vital. El primero está dirigido a la infancia, el segundo a la adolescencia y el tercero al fortalecimiento de las relaciones parentales en el ámbito familiar^(5)^. Iniciativas independientes también ganaron espacio, como la adaptación brasileña del programa australiano *The School Health and Alcohol Harm Reduction Project* (SHAHRP)^(6)^. Esta experiencia generó un proceso mucho más consistente y robusto de producción de evidencias, fortaleciendo la relación entre quienes formulan e implementan políticas preventivas y los institutos de investigación que evalúan estas iniciativas^(5)^. Por otro lado, esta experiencia fue desafiante, ya que la importación de programas estructurados puede traer dificultades relacionadas con las adaptaciones culturales a la realidad local^(7-8)^. Esto puede resultar en pérdida de evidencias debido a alteraciones en los desenlaces, que pueden volverse desfavorables, además de comprometer la fidelidad a los aspectos centrales del programa original y enfrentar los desafíos inherentes a la implementación a gran escala^(5)^. Por lo tanto, fortalecer los procesos de evaluación es una necesidad y un gran desafío.

Comprender mejor cómo han sido los avances en la implementación de acciones preventivas para el uso de drogas y la promoción de la salud en Brasil es necesario para respaldar la implementación de políticas públicas, tales como el *Sistema Nacional de Prevenção do Uso de Álcool e Outras Drogas*, vinculado a la SENAD. De este modo, un estudio detallado de las características metodológicas de las estrategias y programas para el uso de alcohol y otras drogas que ya han pasado por procesos evaluativos puede ayudar en la oferta de un conjunto de acciones preventivas, necesario para la implementación de las políticas preventivas. Así, Brasil podría comenzar a consolidar el desarrollo de una plataforma para la certificación de estrategias y programas, con base en estándares científicos claros, como ya ocurre en otros países. De la misma forma, este análisis ayuda a que la ciencia de la prevención brasileña, aún en proceso de maduración, pueda mirar su historia y reflexionar sobre sus avances y desafíos.

Considerando la problemática descrita anteriormente, esta actual revisión de alcance tiene como objetivo mapear estudios que desarrollaron procesos evaluativos de programas de prevención para el uso de alcohol y otras drogas en Brasil, analizando sus características metodológicas, publicados entre los años 2011 y 2023. Busca contribuir a la elaboración de un conjunto de iniciativas preventivas basadas en evidencias disponibles en Brasil, que pueda respaldar políticas públicas preventivas sobre drogas y ayudar en la toma de decisiones de gestores, profesionales e investigadores.

## Método

### Tipo de estudio

La revisión de alcance fue el método elegido para responder al objetivo de esta investigación, ya que permite el mapeo de la literatura existente en un campo determinado y la sistematización de características conceptuales y metodológicas de investigaciones^(9)^. Esta revisión fue orientada por el *Joanna Briggs Institute Manual* y las directrices del *Preferred Reporting Items for Systematic Reviews and Meta-Analyses extension for Scoping Reviews Checklist* (PRISMA-ScR)^(10)^ y se llevaron a cabo las siguientes etapas: 1) elaboración del protocolo (registrado en PROSPERO bajo el número 2022 CRD42022330854, enlace de acceso: https://www.crd.york.ac.uk/prospero/display_record.php?ID=CRD42022330854), con especificación de la pregunta de investigación, definición de los criterios de elegibilidad, de las bases de datos y de las estrategias de búsqueda; 2) identificación de los estudios relevantes aplicando las estrategias de búsqueda en las bases de datos seleccionadas; 3) selección de los estudios con base en los criterios de elegibilidad; 4) extracción y análisis de los datos; 5) sistematización y resumen de los datos y presentación de los resultados. El protocolo (disponible bajo solicitud a la autora de correspondencia) fue desarrollado por el equipo de investigación, compuesto por especialistas en la ciencia de la prevención en psicología, profesionales con experiencia en revisiones sistemáticas en el área de la salud y un bibliotecario.

La pregunta de investigación que guió esta revisión fue: ¿Cuáles son las características metodológicas de los estudios que investigaron programas de prevención sobre el uso de drogas evaluados en Brasil? Los componentes de la pregunta siguieron el acrónimo PCC (ítem 4 del PRISMA-ScR), en el que los “participantes” (P) fueron estudios empíricos completos realizados con seres humanos; el “concepto” (C) fueron los programas de prevención sobre drogas que pasaron por algún proceso de evaluación (de efecto temporal, eficacia, efectividad o de proceso de implementación); y el “contexto” (C) fue el brasileño.

### Criterios de selección y contexto

Se incluyeron estudios experimentales y cuasiexperimentales, estudios metodológicos, estudios observacionales analíticos y descriptivos, enfoques cualitativos, tesis y disertaciones cuya temática central fuera la aplicación de un programa de prevención sobre drogas evaluado en Brasil. Se incluyeron estudios publicados entre el 1/1/2010 y el 31/12/2023, sin restricciones de idioma. Se excluyeron estudios con población no humana (población incorrecta); editoriales, cartas, erratas, libros, capítulos de libros y trabajos presentados en congresos (tipo de publicación incorrecto); estudios teóricos y revisiones de cualquier naturaleza - integrativa, narrativa, sistemática, meta-análisis (diseño del estudio incorrecto); estudios que investigaron programas de prevención sobre otro foco que no fueran drogas, o que no presentaran resultados de evaluación del programa, o que no fueran realizados en Brasil (tema incorrecto).

La búsqueda sistemática se realizó en dos momentos: la primera, el 31 de agosto de 2021, cuando se inició la presente revisión, y una actualización el 31 de enero de 2024. Las bases de datos consultadas fueron: *Medical Literature Analysis and Retrieval System Online* (MEDLINE) vía *Publisher* MEDLINE (PubMed); *Excerpta Medica Database* (EMBASE); *Psychological Information Database* (PsycINFO); *Web of Science*, *Scientific Electronic Library Online* (SciELO); Literatura Latinoamericana y del Caribe en Ciencias de la Salud (LILACS) e Índice de Literatura Técnico-Científica em Psicologia (IndexPsi), ambas parte de la *Biblioteca Virtual em Saúde* - *Psicologia Brasil* (BVS-Psi Brasil); la proveedora de bases de datos *Elton B. Stephens Company* (EBSCO); y el *Banco de Teses e Dissertações da Coordenação de Aperfeiçoamento de Pessoal de Nível Superior* (CAPES). Las bases fueron elegidas considerando su alcance mundial con enfoque multidisciplinario (por ejemplo, *Web of Science*), con amplio acceso a artículos en el área de la salud (por ejemplo, MEDLINE vía PubMed) y en la psicología (por ejemplo, PsycINFO), y por la característica de indexar artículos publicados en revistas de América Latina y el Caribe (por ejemplo, LILACS y SciELO), ya que el enfoque era en estudios realizados en Brasil.

Los indexadores utilizados en las búsquedas fueron “*Program*”, “*Prevention*”, “*Evaluation*”, “*Drug*”, y “*Brazil*”, y las estrategias de búsqueda completas, incluyendo los descriptores y operadores booleanos utilizados, pueden ser consultadas en https://www.crd.york.ac.uk/PROSPEROFILES/330854_STRATEGY_20220506.pdf. Además de las bases de datos, se consultaron especialistas en el tema de la ciencia de la prevención de Brasil para reducir el sesgo de selección y ampliar las posibilidades de acceder al mayor número de estudios sobre el tema. No se verificaron sistemáticamente todas las referencias de los artículos incluidos, ya que en un análisis preliminar de las mismas no aparecieron artículos nuevos dentro de nuestro período delimitado de búsqueda.

Las referencias obtenidas a partir de las búsquedas en las bases de datos fueron importadas con la ayuda de *EndNote*, donde se eliminaron los duplicados. Posteriormente, las referencias fueron importadas a *Rayyan*®^(11)^ para la selección de los estudios relevantes aplicando los criterios de elegibilidad, en formato doble ciego.

### Recolección, tratamiento y análisis de datos

Los diez primeros resúmenes fueron evaluados en una reunión en línea con todo el equipo, mediante el uso de *Rayyan*®, para uniformizar la lista de razones de exclusión. Posteriormente, ocho juezas independientes realizaron la selección de los estudios a doble ciego a partir de la lectura de los demás títulos y resúmenes. Las discrepancias se resolvieron en reuniones con al menos el 50% del equipo hasta llegar a un consenso. Después de la selección, se utilizó el mismo procedimiento por parte de las ocho juezas para la calibración de la lectura y extracción de los textos completos.

Para la extracción de los datos, el equipo de investigación elaboró una hoja de cálculo matriz en el *softwareExcel*, basada en las directrices del PRISMA-ScR. La hoja de cálculo fue organizada en cinco grandes secciones para la extracción de datos: 1) Caracterización general de los estudios (año de publicación y diseño); 2) Método de los estudios, especificando el público objetivo (número y sexo), uso de instrumentos de evaluación antes y después de la intervención, procedimientos (ej. se realizó *follow-up*) y desenlaces evaluados (tipo de droga, patrón de consumo); 3) Datos sobre el programa de prevención de drogas, como el nombre del programa, el contexto (ej. escolar, salud), el alcance (ej. nacional, regional) y los procedimientos (número de sesiones y duración, tipo de droga a la que está dirigido el programa, tipo – taller, conferencia, vivencia – y categoría de prevención); 4) Datos sobre los procedimientos de evaluación del proceso de implementación del programa, es decir, si se realizaron estudios preparatorios (como levantamiento de necesidades y estudios piloto), estudios de fidelidad, satisfacción, viabilidad y sostenibilidad; y 5) Datos sobre la evaluación de los resultados del programa, es decir, desenlaces que mejoraron, empeoraron o se mantuvieron, y datos sobre eficacia, efectividad y costo-beneficio.

Considerando que esta es una revisión de alcance, que busca presentar de forma resumida las características de los estudios mapeados de programas de prevención sobre drogas realizados en Brasil, no se aplicaron herramientas de análisis de calidad metodológica, riesgo de sesgo ni calidad de la evidencia, ya que estos ítems son aplicables a revisiones sistemáticas que tienen como objetivo evaluar los efectos de intervenciones.

## Resultados

A partir de las búsquedas en las bases seleccionadas, se identificaron 2106 textos, siendo 1631 en las búsquedas realizadas el 31/08/2021 (textos del 01/01/2010 al 31/08/2021) y 475 en las búsquedas realizadas el 31/01/2024 (textos del 01/09/2021 al 31/12/2023). Se sumaron, además, 44 textos indicados tras la consulta a especialistas (con el uso de los mismos descriptores) en dos momentos, septiembre de 2021 y enero de 2024, totalizando 2150 publicaciones encontradas. No se incluyó ningún material a partir de la lectura de referencias de los artículos seleccionados. Tras la exclusión de 296 estudios duplicados (178 en la primera y 118 en la segunda búsqueda), 1854 pasaron a la etapa de selección. Aplicando los criterios de inclusión a partir de la lectura de los resúmenes (razones de exclusión detalladas en la Figura 1), 84 estudios avanzaron a la etapa de lectura del texto completo. De estos, 28 textos fueron eliminados por los criterios de exclusión, quedando 56 publicaciones incluidas para el análisis y síntesis cualitativa. La Figura 1 presenta el diagrama de flujo con los detalles de este proceso.

**Figura 1 -:** Diagrama de flujo de las etapas de búsqueda, cribado, elegibilidad e inclusión

*n = Número de estudios; ^†^MEDLINE vía PubMed = *Medical Literature Analysis and Retrieval System Online* vía *Publisher* MEDLINE; ^‡^EMBASE = *Excerpta Medica Database*; ^§^PsycINFO = *Psychological Information Database*; ^||^LILACS = Literatura Latinoamericana y del Caribe en Ciencias de la Salud; ^¶^ *IndexPsi* = *Índice de Literatura Técnico-Científica em Psicologia*; **EBSCO = *Elton B. Stephens Company*; ^††^SciELO = *Scientific Electronic Library Online*; ^‡‡^CAPES = *Coordenação de Aperfeiçoamento de Pessoal de Nível Superior*

### Tipos de Programas de prevención sobre sustancias psicoactivas en Brasil

Se seleccionaron 56 estudios, siendo 41 artículos, ocho tesis y siete disertaciones. La primera publicación en el área apareció en 2011, abarcando las búsquedas hasta diciembre de 2023, con la mayor frecuencia de publicaciones en los años 2021 (n = 11), 2016 (n = 8), 2020 (n = 8) y 2023 (n = 8). Se observa un aumento de publicaciones en los años posteriores a la implementación de programas coordinados por macroproyectos del *Ministério da Saúde* y la *Secretaria Nacional de Políticas sobre Drogas e Gestão de Ativos/Ministério da Justiça*, evaluados en colaboración con universidades públicas. Las 15 tesis o disertaciones disponibles en catálogos brasileños fueron desarrolladas en la *Universidade Federal de Santa Catarina* (UFSC; n = 06), *Universidade Federal de São Paulo* (UNIFESP; n = 05), *Universidade de Brasília* (UnB; n = 3) y *Harvard University* (n = 1).

Se identificaron once acciones preventivas evaluadas en Brasil, de las cuales nueve son programas preventivos. Entre estos, dos poseen versiones revisadas que deben considerarse como programas distintos. Además de los programas, se encontraron dos estrategias preventivas. Todos serán descritos en mayor detalle en la sección sobre las características de los programas analizados: 1. *El Unplugged/#Tamojunto*, evaluado en 19 estudios, siendo ocho en forma de tesis y disertaciones. A estos se suman otros seis artículos que analizaron su versión revisada, llamada *#Tamojunto 2.0*; 2. El *Strengthening Families Program/Famílias Fortes*, fue evaluado en 10 publicaciones, siendo dos en forma de tesis; 3. El *Drug Abuse Resistance Education* (DARE)/*Programa Educacional de Resistência às Drogas e à Violência* (PROERD), fue evaluado en nueve publicaciones, siendo una tesis; 4. El *Good Behavior Game*/ELOS, fue evaluado en seis publicaciones, siendo tres tesis o disertaciones. A estas se suma una publicación sobre su versión revisada, llamada ELOS 2.0. Con una única publicación se encuentran el *SHAHRP* (llamado en Brasil *Programa de Estímulo à Saúde e Redução de Riscos Associados ao Uso de Álcool Aplicado ao Ambiente Educacional*/PERAE), el *Programa Descolado*, el *Programa Mulheres* (Programa M, presentado en forma de tesis), el *Jogo da Onda Digital* y la intervención *Educação Sobre Drogas e Habilidades Sociais*.

Se destaca que la mayor concentración de investigación y producción en esta temática está en acciones interinstitucionales entre los grupos de investigación de la UNIFESP y la UFSC, sobre los programas *#Tamojunto* y ELOS, totalizando 25 colaboraciones, ya que incluso en las disertaciones y tesis de cada universidad los profesores de la otra participaban en los tribunales de examen y en los artículos resultantes en conjunto. La UNIFESP también tiene protagonismo individual en otras 17 publicaciones, aunque de grupos diferentes dentro de la misma institución. La UnB tiene 10 publicaciones, algunas de estas en colaboración con otras instituciones.

### Regiones brasileñas y muestra de los Programas de prevención sobre alcohol y otras drogas en Brasil

De los 56 estudios seleccionados, la mayoría fue conducida con muestra de alcance nacional (n = 27), abarcando más de una región brasileña. En segundo lugar están las implementaciones de ámbito municipal (n = 15), seguidas de las regionales, predominantemente en el Nordeste (n = 7), y las estatales, desarrolladas en el estado de São Paulo (n = 7). Las regiones más investigadas fueron Sudeste (n = 39, siendo 37 en el estado de São Paulo), Nordeste (n = 28, siendo 22 en Ceará), Sur (n = 22, en su totalidad en el estado de Santa Catarina) y Centro-Oeste (n =11, siendo 10 en el Distrito Federal). No hubo ningún estudio desarrollado en la Región Norte.

Los estudios incluidos en esta revisión fueron conducidos mayoritariamente con estudiantes/adolescentes (n = 38), facilitadores/multiplicadores de los programas (n = 11), profesores (n = 8) y equipos pedagógicos de las escuelas (n = 5), así como con padres (n = 6). Predominantemente, fueron desarrollados en el contexto escolar (n = 44) o de la asistencia social (n = 9), con un estudio realizado en el ámbito comunitario (n = 1) y otro en un servicio de salud mental (n = 1). El número de participantes varió de dos a 6637 (Media = 2212; DE = 2513; mediana = 809). Mayoritariamente fueron conducidos con personas de ambos sexos, a excepción de dos que usaron muestra exclusivamente femenina^(12-13)^ y uno exclusivamente masculina^(14)^.

### Características de los Programas de prevención sobre el uso de drogas evaluados en Brasil

En relación con la realización de estudios preparatorios para la implementación de los programas preventivos, solo dos mencionan la identificación de necesidades. Tres estudios se refirieron a estudios de evaluabilidad, mientras que 17 mencionaron la realización de estudios piloto. En cuanto al diseño, la mayoría de los estudios fueron observacionales, generalmente asociados a la evaluación de procesos (n = 25, de los cuales cinco provienen de disertaciones y cuatro de tesis); seguidos por los experimentales (n = 23), relacionados con estudios de efectividad. Los estudios cuasiexperimentales fueron menos frecuentes (n = 4) y están vinculados a estudios de eficacia. Los estudios preexperimentales también suman cuatro (n = 4) y están relacionados con resultados temporales. Se utilizaron enfoques cuantitativos (n = 23), mixtos (n = 20) y cualitativos (n = 13). La temporalidad predominante de las evaluaciones fue longitudinal (n = 36).

La mayoría de los estudios evaluó los resultados de los programas (n = 30), incluyendo análisis de resultados objetivos (n = 22), y además de estos, también de efectos secundarios, moderadores o mediadores de los resultados (n = 18). Seis estudios mencionaron la realización de estudios de impacto y ninguno realizó análisis de costo/beneficio. En la discusión de los resultados, 24 estudios reportaron que los resultados objetivos mejoraron en alguna medida, 10 informaron que algunos resultados empeoraron y 17 demostraron efectos nulos sobre algunos de los resultados previstos.

Hubo 26 estudios dirigidos a la evaluación del proceso de implementación. Gran parte de estos trabajos realizaron evaluaciones de diferentes resultados en los procesos, siendo que la mayoría se dedicó a algún tipo de evaluación de fidelidad (n = 22), con énfasis en el análisis de la dimensión de la exposición, que incluye la verificación de la dosis proporcionada, la dosis recibida y el alcance (n = 19). Le siguen los estudios que analizaron la calidad de la implementación (n = 8), la formación del equipo (n = 3) y la adherencia (n = 4). Además de la fidelidad, estos trabajos también evaluaron otras dimensiones del proceso, de los cuales 17 mencionaron la evaluación de la satisfacción o aceptabilidad de los programas, 12 estudios de viabilidad para la realidad brasileña y 11 investigaron aspectos del contexto interno y externo que influyeron en la implementación. Un único estudio analizó la sostenibilidad del *Programa Familias Fortes*. No se encontraron estudios sobre la difusión de los programas.

La Figura 2 presenta la descripción del tipo de evaluación realizada: proceso o resultado, el diseño metodológico y una síntesis de los resultados obtenidos con los programas preventivos, según los estudios incluidos en orden cronológico.

**Figura 2 -:** Características metodológicas de los estudios encontrados que realizaron la evaluación de programas para la prevención del consumo de drogas en Brasil según autor, tipo de evaluación, nombre del programa/participantes, diseño y resultado del estudio. Florianópolis, SC y São Paulo, SP, Brasil, 2024

**Autor, A** ñ **o**	**Programa (Nombre original)**	**Tipo de evaluación**	**Diseño/Participantes**	**Evaluación de resultado - objetivo**
Rocha, 2011 ^( 13 )^	*Programa Mulheres*	Resultado	Cuasiexperimental, Longitudinal Mujeres (n* = 273)	Aumento de la autoeficacia en las relaciones interpersonales de las mujeres y reducción del consumo de drogas.
Shamblen et al., 2014 ^( 15 )^	PROERD ^†^ (DARE ^‡^ )	Resultado	Cuasiexperimental, Longitudinal Estudiantes, Adolescentes (n* = 2995)	Inocuo para efectos en el uso de sustancias, comportamiento antisocial y de riesgo para el uso de sustancias.
Horr, 2015 ^( 16 )^	# *Tamojunto* ( *Unplugged* )	Proceso	Observacional, Transversal Estudiantes, Adolescentes (n* = 353)	Satisfacción positiva de los educandos al participar en el programa, indicando buena aceptabilidad.
Peres, et al., 2015 ^( 17 )^	# *Tamojunto* ( *Unplugged* )	Proceso	Observacional, Transversal Profesionales de la salud, profesores, multiplicadores del programa (n* = 19)	La implementación fortaleció vínculos entre salud y educación. El programa tuvo alta aceptabilidad por parte de gestores, coordinadores, multiplicadores, profesionales de la salud y educación, y padres.
Sanchez, et al., 2016 ^( 18 )^	# *Tamojunto* ( *Unplugged* )	Resultado	Cuasiexperimental (ECA ^§^ ), Longitudinal Estudiantes, Adolescentes (n* = 2459)	Disminución del uso reciente de marihuana entre estudiantes de 13 a 15 años. No hubo evidencias de efecto en alumnos de 11 a 12 años.
Medeiros, et al., 2016 ^( 19 )^	# *Tamojunto* ( *Unplugged* )	Proceso	Estudiantes, Profesores, multiplicadores y gestores (n* = 84)	Los índices de implementación y monitoreo de las clases alcanzaron el 94%. La duración de las clases fue insuficiente para cubrir todas las actividades propuestas. Profesores y alumnos demostraron satisfacción con el programa.
Schneider, et al., 2016 ^( 8 )^	ELOS ( *Good Behavior Game* )	Proceso	Observacional, Longitudinal Profesores, Equipo Pedagógico de las Escuelas, *Coaches* (n* = 43)	Alta aceptabilidad por parte de profesores y directores, atribuida a su estrategia de manejo del aula. Hubo indicaciones de la necesidad de adaptaciones que reflejen el contexto social y económico brasileño.
Strelow, 2016 ^( 20 )^	ELOS ( *Good Behavior Game* )	Proceso	Observacional, Transversal Estudiantes, Adolescentes (n* = 27)	Los niños demostraron alta aceptabilidad del ELOS y expresaron la percepción de resultados en un aumento de la colaboración con compañeros y el deseo de continuar con el juego.
Lopes, 2016 ^( 14 )^	# *Tamojunto* ( *Unplugged* )	Proceso	Observacional, Longitudinal Profesores (n* = 53)	Los profesores gradualmente comenzaron a interesarse más por la práctica de la prevención y por el programa. Se evaluó un cambio en el discurso, de un enfoque moralista y estigmatizante hacia una perspectiva más comprensiva.
Medeiros, 2016 ^( 21 )^	# *Tamojunto* ( *Unplugged* )	Proceso	Observacional, Longitudinal Estudiantes, Adolescentes (n* = 1336)	Implementación en el 94% de las clases, pero solo el 57% con fidelidad al manual. Se identificó la exclusión de algunas actividades, dificultades en la planificación por parte de los profesores y falta de apoyo de los administradores. Se reportaron mejoras en el ambiente del aula y en las habilidades personales.
Martins, 2016 ^( 22 )^	# *Tamojunto* ( *Unplugged* )	Resultado	Correlacional, Transversal Estudiantes, Adolescentes (n* = 5007)	El análisis del perfil de la muestra para comprender posibles moderadores de los efectos del programa identificó la relación entre estilos parentales y el uso de drogas.
Menezes, 2016 ^( 12 )^	*Famílias Fortes* ( *Strengthening Families Program* )	Proceso	Observacional, Transversal Profesionales de la Salud, Facilitadores, Multiplicadores del programa (n* = 41)	La adaptación cultural se realizó con calidad. Sin embargo, el programa necesita ser adecuado a los aspectos relacionados con las desigualdades sociales y el analfabetismo de la población, lo que implica nuevas adaptaciones.
Sanchez, et al., 2017 ^( 23 )^	# *Tamojunto* ( *Unplugged* )	Resultado	Experimental (ECA ^§^ ), Longitudinal Estudiantes, Adolescentes (n* = 4253)	Resultado que mejoró: retraso en el primer uso de inhalantes. Resultado que empeoró: primer uso de alcohol.
Peres, et al., 2017 ^( 24 )^	# *Tamojunto* ( *Unplugged* )	Proceso	Observacional, Transversal Profesionales de la salud, profesionales de la educación (n* = 18)	Percepción positiva de los profesionales sobre estrategias conjuntas para la implementación del programa y fortalecimiento de la conexión entre salud y escuela. Se evaluaron desafíos en la comprensión de la intersectorialidad en la práctica.
Conegundes, 2017 ^( 25 )^	# *Tamojunto* ( *Unplugged* )	Resultado	Experimental (ECA ^§^ ), Longitudinal Estudiantes, Adolescentes (n* = 6387)	Los estudiantes que participaron en el grupo de intervención y que no vivían con la madre aumentaron la probabilidad de consumo de alcohol en comparación con el grupo de control.
Pedroso, 2017 ^( 26 )^	# *Tamojunto* ( *Unplugged* )	Proceso	Observacional, Longitudinal Profesores, Facilitadores, Multiplicadores (n* = 671)	Para la sostenibilidad y diseminación del programa como política pública, se recomienda invertir en un cambio paradigmático en el enfoque de drogas por parte de los implementadores, aumentar el uso de metodologías interactivas, ajustar el tiempo de las clases y ampliar la intersectorialidad.
Sanchez, et al., 2018 ^( 27 )^	# *Tamojunto* ( *Unplugged* )	Resultado	Experimental (ECA ^§^ ), Longitudinal Estudiantes, Adolescentes (n* = 5007)	Los resultados indican que el programa retrasó el uso de inhalantes, pero empeoró el primer consumo de alcohol entre los estudiantes que participaron en el programa.
Murta, et al., 2018 ^( 28 )^	*Famílias Fortes* ( *Strengthening Families Program* SFP 10-14-UK)	Proceso	Observacional, Longitudinal Estudiantes, Adolescentes, Padres, Facilitadores, Multiplicadores del Programa (n* = 33)	El programa fue percibido como culturalmente relevante y parcialmente claro. Las adaptaciones culturales deben centrarse en aspectos lingüísticos de los materiales y procedimientos, considerando las diferencias culturales, económicas y educativas de las familias, y deben ser conducidas de forma participativa.
Medeiros, et al., 2018 ^( 29 )^	# *Tamojunto* ( *Unplugged* )	Proceso	Observacional, Transversal Estudiantes, Adolescentes, Profesores, Equipo pedagógico de las escuelas (n* = 78)	Las facilidades para la implementación incluyen el soporte técnico, la formación, la supervisión y el apoyo de los administradores escolares. Las dificultades involucraron el tiempo de las clases, la obtención de materiales de apoyo y el cumplimiento del currículo regular. Como potencialidad, se destacó la mejora en la convivencia.
Gusmões 2018 ^( 30 )^	# *Tamojunto* ( *Unplugged* )	Resultado	Experimental (ECA ^§^ ), Longitudinal Estudiantes, Adolescentes (n* = 6637)	Redujo el *bullying* en los primeros 9 meses, pero el efecto no se mantuvo después de 21 meses. Los factores de riesgo para el *bullying* incluyeron participación previa en violencia, consumo de alcohol e inhalantes, y madres con episodios de embriaguez.
D’Tôllis, 2018 ^( 31 )^	ELOS ( *Good Behavior Game* )	Proceso	Observacional, Longitudinal Gestores nacionales (n* = 2)	La adaptación cultural fue evaluada como satisfactoria. Los aspectos de planificación de la implementación, sensibilidad cultural, fidelidad al programa original y los procesos evaluativos y de refinamiento fueron bien desarrollados.
Garcia, 2018 ^( 32 )^	ELOS ( *Good Behavior Game* )	Proceso	Observacional, Longitudinal Profesores, Equipos de las Escuelas, Facilitadores (n* = 60)	La evaluación de la fidelidad de la implementación fue positiva, pero los resultados fueron parcialmente inconclusos debido a problemas en la devolución de los formularios de fidelidad por parte de profesores y multiplicadores.
Abdala, 2018 ^( 33 )^	*Famílias Fortes* ( *Strengthening Families Program* )	Proceso	Observacional, Transversal Facilitadores, Multiplicadores del Programa (n* = 26)	Las barreras señaladas por los facilitadores para la implementación incluyeron condiciones de trabajo inadecuadas, una administración municipal frágil, metodologías de capacitación insuficientes y baja adhesión de los profesionales.
Sanchez, et al., 2019 ^( 34 )^	# *Tamojunto* ( *Unplugged* )	Resultado	Experimental (ECA ^§^ ), Longitudinal Estudiantes, Adolescentes (6391)	Los resultados sugieren la falta de efecto del programa sobre las creencias normativas en relación con el consumo de drogas.
Murta, et al., 2020 ^( 7 )^	*Famílias Fortes* ( *Strengthening Families Program* )	Resultado	Pre-Experimental, Longitudinal Estudiantes, Adolescentes, Padres y Facilitadores, Multiplicadores del Programa (n* = 126)	Aumento en la autoeficacia del aprendizaje y disminución de la ausencia escolar sin permiso de los padres. Efectos nulos sobre el consumo de alcohol, episodios de *binge* *drinking* y comportamiento antisocial en el último mes.
Abdala, et al., 2020 ^( 35 )^	*Famílias Fortes* ( *Strengthening Families Program* )	Proceso	Observacional, Transversal Facilitadores, Multiplicadores del Programa (n* = 26)	Las barreras presentadas por los facilitadores incluyeron condiciones de trabajo, administración municipal débil, infraestructura precaria, grupo inadecuado, metodologías de capacitación de facilitadores, baja adhesión de los gestores y escasez de financiamiento.
Valente, et al., 2020 ^( 36 )^	# *Tamojunto* ( *Unplugged* )	Resultado	Experimental (ECA ^§^ ), Longitudinal Estudiantes, Adolescentes (n* = 6391)	Los resultados demostraron una reducción en la habilidad de toma de decisiones y un aumento en el consumo de drogas y violencia.
Valente, et al., 2020 ^( 37 )^	# *Tamojunto* ( *Unplugged* )	Resultado	Experimental (ECA ^§^ ), Longitudinal Estudiantes, Adolescentes (n* = 6391)	No se identificó un potencial “refuerzo” de los comportamientos parentales sobre el uso de drogas entre aquellos que recibieron la intervención. Se identificó una asociación entre menor exigencia parental y mayor consumo de drogas por parte de los adolescentes.
Menezes, et al., 2020 ^( 38 )^	*Famílias Fortes* ( *Strengthening Families Program* )	Proceso	Observacional, Transversal Facilitadores, Multiplicadores del Programa (n* = 42)	Respecto a la fidelidad, la mitad de los facilitadores informó haber excluido ítems de los procedimientos y el 73,2% de los entrevistados declaró haber hecho una o más modificaciones al aplicar el programa.
Murta, et al., 2020 ^( 39 )^	*Famílias Fortes* ( *Strengthening Families Program* SFP 10-14-UK)	Proceso	Observacional, Longitudinal Estudiantes, Adolescentes, Padres (n* = 74)	Buenos niveles de fidelidad y participación de los padres, y niveles moderados de participación de los adolescentes. La intervención es relevante y los procedimientos son aceptables, pero se identificó la necesidad de adaptaciones culturales para facilitar la comprensión en familias con baja escolaridad.
Coelho y Monteiro, 2020 ^( 40 )^	*Jogo da Onda Digital*	Proceso	Observacional, Transversal Estudiantes, Adolescentes (n* = 12)	Percepción de mejora en la comprensión de temas relacionados con el consumo de drogas. Los participantes valoraron el enfoque interactivo y lúdico del juego, que se destacó en comparación con los enfoques educativos tradicionales.
Valente, 2020 ^( 41 )^	# *Tamojunto* ( *Unplugged* )	Resultado	Experimental (ECA ^§^ ), Longitudinal Estudiantes, Adolescentes (n* = 6391)	El programa alteró las habilidades de toma de decisiones, pero en la dirección opuesta a la propuesta por el modelo teórico del programa. La intervención no cambió el consumo de drogas.
Amato et al., 2021 ^( 6 )^	Programa PERAE ^||^ (SHAHRP ^¶^ )	Proceso	Observacional, Longitudinal Estudiantes, Adolescentes (n* = 348)	La fidelidad de implementación alcanzó el 66,8%. El porcentaje de clases completas implementadas varió del 62,5% al 87,5%. Los alumnos y profesores informaron una buena aceptabilidad del programa.
Murta, et al., 2021 ^( 42 )^	*Famílias Fortes* ( *Strengthening Families Program* )	Proceso	Observacional, Longitudinal Estudiantes, Adolescentes, Padres, Facilitadores (n* = 410)	Se evaluó que la satisfacción/aceptabilidad resultó en un aumento de la viabilidad. El programa mejoró la cohesión familiar, pero aumentó la sobrecarga de los facilitadores.
Valente y Sanchez, 2021 ^( 43 )^	PROERD ^†^ (DARE ^‡^ *Keepin’ it REAL* )	Resultado	Experimental (ECA ^§^ ), Longitudinal Estudiantes, Adolescentes, (n* = 4030)	Mejoró la experiencia escolar, pero empeoró la intención de consumir drogas, las habilidades de toma de decisiones y las habilidades para rechazar ofertas de drogas.
Sanchez, et al., 2021 ^( 44 )^	# *Tamojunto* 2.0 ( *Unplugged* )	Resultado	Experimental (ECA ^§^ ), Longitudinal Estudiantes, Adolescentes (n* =5208)	Redujo las probabilidades de que los alumnos de 8º grado iniciaron el consumo de alcohol. No se encontraron efectos sobre el inicio de otras drogas o la prevalencia de uso mensual.
Sanchez, et al., 2021 ^( 45 )^	PROERD ^†^ (DARE ^‡^ *Keepin’ it REAL* - kiR)	Resultado	Experimental (ECA ^§^ ), Longitudinal Estudiantes, Adolescentes (n* = 4030)	Presentó un efecto negativo, indicando que los alumnos de 7º grado que ya practicaban *binge drinking* antes y que asistían al programa tenían mayores probabilidades de mantener ese consumo.
Cogo-Moreira, et al., 2021 ^( 46 )^	# *Tamojunto* ( *Unplugged* )	Resultado	Experimental (ECA ^§^ ), Longitudinal Estudiantes, Adolescentes (n* = 6390)	Los resultados indicaron que el comportamiento violento temprano predice un mayor consumo de drogas entre adolescentes y muestran la falta de evidencia de que # *Tamojunto* cambie la dinámica entre el uso de drogas y el *bullying* .
Pinheiro-Carozzo, et al., 2021 ^( 47 )^	*Famílias Fortes* ( *Strengthening Families Program* )	Resultado	Pre-Experimental, Longitudinal Estudiantes, Adolescentes, Padres (n* = 361)	Afecta las dimensiones parentales de forma diferente dependiendo del estilo parental inicial y puede ayudar a mejorar debilidades en el cumplimiento de los deberes parentales a corto plazo.
Ferreira Junior, 2021 ^( 48 )^	PROERD ^†^ (DARE ^‡^ )	Resultado	Experimental (ECA ^§^ ), Longitudinal Estudiantes, Adolescentes (n* = 4045)	El consumo de alcohol se asoció con todas las categorías de *bullying* . El bajo rendimiento académico también estuvo fuertemente asociado al *bullying* . Los alumnos negros/mestizos tuvieron 3,35 veces más probabilidades de ser altamente victimizados por *bullying* .
Mariano, et al., 2021 ^( 49 )^	ELOS 2.0 ( *Good Behavior Game* )	Resultado	Experimental (ECA ^§^ ), Longitudinal No informado	Protocolo del estudio de efectividad.
Machado, et al., 2021 ^( 50 )^	*Educação Sobre Drogas e Habilidades Sociais*	Proceso	Observacional, Longitudinal Estudiantes, Adolescentes (n* = 145)	La mayoría de los estudiantes aprobó el programa, favoreciendo la posibilidad de continuidad en los años siguientes.
Farias, 2021 ^( 51 )^	*Famílias Fortes* ( *Strengthening Families Program* )	Proceso	Observacional, Transversal Gestores (n* = 7)	Se mencionaron variables relacionadas con la capacidad organizativa, la adaptación del programa, el apoyo político, la evaluación del programa y las asociaciones como esenciales para la sostenibilidad del programa.
Gusmões et al., 2022 ^( 52 )^	PROERD ^†^ (DARE ^‡^ *Keepin’ it REAL* - kiR)	Resultado	Experimental, Longitudinal Estudiantes, Adolescentes e Instructores del PROERD (n* = 4030)	El nivel de fidelidad a la implementación no influyó en la capacidad del programa para reducir el consumo de drogas entre adolescentes. Sin embargo, el análisis cualitativo reveló adaptaciones realizadas por los instructores policiales.
Melo, et al., 2022 ^( 53 )^	# *Tamojunto* 2.0 ( *Unplugged* )	Proceso	Observacional, Longitudinal Profesores y Directores, Coordinadores Pedagógicos (n* = 21)	Baja fidelidad de implementación, buena calidad en la aplicación del programa y alta inasistencia de los estudiantes.
Schneider, et al., 2022 ^( 54 )^	ELOS ( *Good Behavior Game* )	Resultado	Cuasiexperimental, Longitudinal Estudiantes, Adolescentes (n* = 1731)	Reduce la agresividad y el comportamiento disruptivo en niños. No tuvo el mismo efecto en niñas.
Valente, et al., 2022 ^( 55 )^	PROERD ^†^ (DARE ^‡^ , *Keepin’ it REAL* - kiR)	Resultado	Experimental, Longitudinal Estudiantes, Adolescentes (n* = 4300)	No hay evidencia de efectos en el cambio de patrones de consumo de drogas al comparar la intervención con el grupo control en ninguno de los currículos del PROERD dirigidos a alumnos de 5º y 7º grado.
Garcia-Cerde, et al., 2022 ^( 56 )^	# *Tamojunto* 2.0 ( *Unplugged* )	Resultado	Experimental, Longitudinal Estudiantes, Adolescentes (n* = 5208)	Aumentó el conocimiento sobre drogas y las creencias negativas/no positivas sobre el alcohol. No se encontró evidencia de efecto del programa en el consumo de marihuana.
Almeida, et al., 2023 ^( 57 )^	# *Tamojunto* 2.0 ( *Unplugged* )	Resultado	Experimental, Longitudinal Estudiantes, Adolescentes (n* = 5208)	Factores como ser mujer, mayor edad, uso previo de drogas y mayor presencia de síntomas de salud mental fueron los principales predictores del consumo de drogas durante el seguimiento.
Gusmões, et al., 2023 ^( 58 )^	PROERD ^†^ (DARE ^‡^ , *Keepin’ it REAL* - kiR)	Proceso	Observacional, Transversal Policías Militares (n* = 19)	La mayoría de los instructores adaptan el programa. Las principales razones extrínsecas para las modificaciones fueron los desafíos derivados de la realidad cultural, el rendimiento escolar de los estudiantes y la falta de apoyo de la escuela. La razón intrínseca fue el desempeño de los instructores.
Valente, et al., 2023 ^( 59 )^	# *Tamojunto* 2.0 ( *Unplugged* )	Resultado	Experimental, Longitudinal Estudiantes, Adolescentes (n* = 5208)	Redujo indirectamente el *bullying* al disminuir el consumo de alcohol. Sin embargo, el programa no fue directamente eficaz en la reducción de la prevalencia del *bullying* (victimización y perpetración).
Valente y Sanchez, 2023 ^( 60 )^	PROERD ^†^ (DARE ^‡^ , *Keepin’ it REAL* - kiR)	Resultado	Experimental, Longitudinal Estudiantes, Adolescentes (n* = 4030)	No tuvo efecto en las habilidades de toma de decisiones, actitudes hacia las drogas, habilidades de rechazo y habilidades de comunicación, según lo propuesto por el modelo teórico.
Garcia-Cerde, et al., 2023 ^( 61 )^	# *Tamojunto* 2.0 ( *Unplugged* )	Resultado	Experimental, Longitudinal Estudiantes, Adolescentes (n* = 5208)	Redujo indirectamente el consumo de alcohol a lo largo de la vida al aumentar las creencias negativas sobre el alcohol. Aunque se observó un efecto indirecto en la disminución del consumo excesivo de alcohol, el efecto directo en la reducción del consumo de alcohol fue estadísticamente significativo.
Oliveira, et al., 2023 ^( 62 )^	*Descolado*	Proceso	Observacional, Transversal Informantes clave del Programa (n* = 6)	El programa es evaluable. Los resultados de la evaluación ayudaron en la organización y descripción lógica del programa, así como en la mejora de su estructuración, indicando la necesidad de ajustes en los objetivos, actividades y recursos.
Ferreira Junior, et al., 2023 ^( 63 )^	PROERD ^†^ (DARE ^‡^ , *Keepin’ it REAL* - kiR)	Resultado	Experimental, Longitudinal Estudiantes, Adolescentes (n* = 4030)	Asociación entre resultados de *bullying* , violencia escolar, patrones de consumo de drogas y descriptores socioeconómicos.
Schneider, et al., 2023 ^( 64 )^	ELOS ( *Good Behavior Game* )	Resultado	Preexperimental, Longitudinal Estudiantes, Niños (n* = 624)	Los grupos que recibieron el programa con mayor fidelidad mostraron resultados más positivos en la disminución de los comportamientos objetivos. Las clases con baja fidelidad presentaron resultados inofensivos o posiblemente iatrogénicos.

*n = Número de participantes; ^†^PROERD = *Programa Educacional de Resistência às Drogas e à Violência*; ^‡^DARE = *Drug Abuse Resistance Education*; ^§^ECR = Ensayo Clínico Aleatorizado; ^||^PERAE = *Programa de Estímulo à Saúde e Redução de Riscos Associados ao Uso de Álcool Aplicado ao Ambiente Educacional*; ^¶^SHAHRP = *The School Health and Alcohol Harm Reduction Project*

### Programas de Prevención sobre el uso de drogas en Brasil

#### Características de los programas y estrategias preventivas sobre el uso de drogas en Brasil

De los once programas o estrategias de prevención del uso de drogas que fueron evaluados en Brasil e identificados en esta revisión, siete son programas de prevención universal, dos son versiones actualizadas de programas ya evaluados anteriormente, uno es un programa de prevención selectiva y dos se configuran como estrategias preventivas. El formato de entrega generalmente fue diseñado como clase o alguna estrategia pedagógica, como taller o juego, como es el caso del Programa ELOS y el *Jogo da Onda Digital*. La duración media de implementación diaria de los programas es de 45 a 50 minutos, correspondiente a una hora de clase (variando de 30 minutos a 2h30 minutos).

En relación con la sustancia objetivo de la intervención, hay solo un programa dirigido específicamente al alcohol, que es el *Programa de Estímulo à Saúde e Redução de Riscos Associados ao Consumo de Álcool Aplicado ao Ambiente Educativo* (PERAE), adaptación cultural del SHAHRP. Todos los otros abordan el consumo de alcohol y otras drogas, destacando el tabaco, la marihuana, los inhalantes, la cocaína y el crack. También hay programas que no mencionan sustancias psicoactivas específicas. Este es el caso del Programa ELOS, por estar dirigido a niños y no incidir directamente sobre el consumo de alcohol y otras drogas, sino en sus factores preventivos a medio y largo plazo. A continuación, se presenta una síntesis de cada programa estudiado.

El programa más evaluado en Brasil es el #*Tamojunto*, adaptación cultural del europeo *Unplugged*, realizado en escuelas con alumnos del 8º año de la educación primaria. Se identificaron 19 estudios evaluativos, de los cuales ocho se centraron en la evaluación de procesos, incluyendo la aceptabilidad de diferentes actores, la viabilidad, la fidelidad, el análisis de la intersectorialidad y su inserción en las políticas públicas^(14,16-17,19,21,24,26,29)^. En términos de evaluación de resultados, hubo un estudio de eficacia, relacionado con la implementación piloto realizada en 2014^(18)^ y dos estudios de efectividad desarrollados en la implementación de 2016, uno con seguimiento de nueve meses y otro de 21 meses^(23,27)^. Cuatro estudios abordaron efectos secundarios, como la relación entre el uso de drogas y el *bullying*/violencia escolar, estilos parentales y *binge drinking* ^(22,25,30,37)^. Otros tres estudios evaluaron los mediadores de los efectos del programa en la implementación de 2016^(34,36,41)^.

Tras los resultados negativos, el programa fue revisado entre 2018 y 2019, definido como #*Tamojunto* 2.0, que se acercó al contenido original del *Unplugged*. Hubo seis estudios evaluativos, siendo uno de evaluación de procesos, centrado en la fidelidad^(53)^, y un estudio de efectividad^(44)^ que mostró que los estudiantes expuestos al programa presentaron menor probabilidad de iniciar el consumo de alcohol que los del grupo de control. También se investigaron los efectos secundarios en la relación entre el uso de drogas y el *bullying* ^(59)^, dos estudios de análisis de mediación que evaluaron qué mecanismos funcionaron para lograr los efectos del programa^(56,61)^ y un estudio sobre la moderación de la condición de salud mental y el patrón de consumo de los adolescentes participantes del proyecto^(57)^.

En cuanto al Programa ELOS: *Construindo Coletivos*, una adaptación cultural del programa estadounidense *Good Behavior Game* (GBG), se encontraron seis estudios evaluativos, cuatro de ellos centrados en la evaluación de procesos^(8,20,31-32)^. Un estudio de resultados temporales mediado por la fidelidad, relacionado con la implementación de 2014^(64)^ y un estudio de eficacia de la implementación de 2016, que apuntó a la disminución de la agresividad y la disruptividad en los niños (pero no en las niñas), corroborando los resultados internacionales del GBG^(54)^. El ELOS 2.0 resultó de una modificación de algunos procedimientos de la primera versión del ELOS, habiéndose localizado un registro del protocolo de un estudio de efectividad^(49)^.

El programa *Famílias Fortes* es una adaptación cultural del *Strengthening Families Program* (SFP), de origen inglés. Se encontraron nueve estudios evaluativos, siendo uno un estudio de levantamiento de necesidades para la adaptación cultural^(28)^, una tesis sobre el proceso de adaptación cultural^(12)^ y un estudio sobre adaptaciones locales para el Nordeste^(38)^. Cuatro estudios evaluaron el proceso de implementación^(35,39,42,51)^. Dos estudios de resultados temporales demostraron el aumento en la autoeficacia del aprendizaje escolar, pero efecto nulo para el cambio en el consumo de alcohol y drogas en los adolescentes^(7)^. Por otro lado, hubo mejora en la relación entre padres e hijos y en los estilos parentales^(47)^.

El PROERD es la actividad preventiva más implementada en escuelas del país, estando presente en todas las regiones brasileñas. Se encontraron nueve estudios, siendo el primero de ellos un estudio cuasiexperimental de 2014, cuando el PROERD aún seguía el modelo del programa DARE, de Estados Unidos, sin encontrar efectos significativos en los resultados objetivo^(15)^. Tres estudios realizados entre 2020 y 2021 analizaron resultados sobre la efectividad del PROERD, ya bajo el modelo del *Keepin’ it REAL*, que indicaron no poseer evidencias de efectos en el cambio del patrón de consumo de drogas, habiendo empeorado el consumo en forma de *binge drinking* ^(43,45,55)^. Se realizaron análisis de los efectos sobre el *bullying* ^(48,63)^ y, en análisis de mediación, se encontró un empeoramiento en las habilidades de comunicación, habilidades de toma de decisiones, actitudes hacia las drogas y habilidades de rechazo^(60)^. Los estudios también evaluaron el proceso de implementación del PROERD para comprender las razones de los resultados inofensivos o potencialmente iatrogénicos^(52,58)^.

El PERAE, adaptación cultural del australiano SHAHRP, fue evaluado en un único estudio sobre la viabilidad del programa para la realidad brasileña, con hallazgos positivos^(6)^. Es un programa australiano, basado en la reducción de daños para el alcohol en jóvenes, que demostró evidencias en su país.

El *Descolado*, creado en 2018 por la *Prefeitura de Recife* en asociación con la *Universidade Federal de Pernambuco* (UFPE), es un programa de prevención universal al uso de alcohol y otras drogas, basado en habilidades para la vida y en el método inspirado en Paulo Freire, implementado en las escuelas de la ciudad. El estudio analizado discute la evaluabilidad del *Descolado* ^(62)^.

El *Jogo da Onda Digital*, una estrategia educativa sobre drogas, busca contribuir a la educación preventiva mediante la enseñanza de Ciencias y Biología. Originalmente desarrollado en 2003^(65)^, fue adaptado a la modalidad digital en 2019. El artículo encontrado realizó una evaluación de proceso del juego digital^(40)^.

El Programa M (*M, de Mulheres*) es una intervención dirigida a mujeres jóvenes de comunidades de bajos ingresos en la ciudad de Rio de Janeiro, centrada en el cambio de actitudes, autoeficacia, conocimiento y varios comportamientos de riesgo para la salud de estas mujeres, incluyendo el uso de alcohol y otras drogas. El programa fue analizado en una tesis doctoral defendida en la Universidad de Harvard, mediante una evaluación de eficacia^(13)^.

La estrategia *Educação Sobre Drogas e Habilidades Sociais* es una intervención realizada en escuelas públicas estatales en el interior del estado de Espírito Santo, con 10 encuentros temáticos sobre alcohol y otras drogas y habilidades sociales en clases de educación física. El estudio realizó una evaluación de proceso^(50)^.

Figura 3 presenta la descripción detallada de todos los programas preventivos al uso de drogas identificados en la revisión.

**Figura 3 -:** Descripción de los Programas de prevención del consumo de drogas evaluados en Brasil, según el nombre del programa, tipo de evaluación y categoría, modelo teórico, intervención y resultado. Florianópolis, SC y São Paulo, SP, Brasil, 2024

**Nombre del programa**	**Tipo de evaluación**	**Tipo y categoría**	**Modelo teórico**	**Intervención**	**Resultado-Objetivo**
# *Tamojunto*	Proceso (n*=9) Resultado (n*=10)	Programa de Prevención Universal	Teoría del aprendizaje social, habilidades para la vida, modelo de creencias en salud y teoría de acción-actitud crítica	Clases / 45-60 min. / 12 sesiones / realizadas por profesores capacitados en escuelas públicas, con apoyo de facilitadores	Retrasar el inicio y reducir el patrón de consumo de alcohol, tabaco, marihuana, inhalantes, cocaína y crack
# *Tamojunto* 2.0	Proceso (n*=1) Resultado (n*=5)	Programa de Prevención Universal	Teoría del aprendizaje social, habilidades para la vida, modelo de creencias en salud y teoría de acción-actitud crítica	Clases / 45-60 min. / 12 sesiones / realizadas por profesores capacitados en escuelas públicas, con apoyo de facilitadores	Retrasar el inicio y reducir el patrón de consumo de alcohol, tabaco, marihuana, inhalantes, cocaína y crack
*Famílias Fortes*	Proceso (n*=8) Resultado (n*=2)	Programa de Prevención Universal	Teoría de los sistemas familiares, teoría de la cognición social, modelo de resiliencia y modelos socioecológicos	Talleres / 2h a 2h30 min. / 7 a 11 sesiones, con padres y adolescentes (10-14 años), realizado en los CRAS ^†^ , por profesionales del SUAS ^‡^	Reducir el patrón de consumo de alcohol y otras drogas en adolescentes y modificar patrones de relación entre padres e hijos y estilos parentales
ELOS: *Construindo Coletivos*	Proceso (n*=5) Resultado (n*=1)	Programa de Prevención Universal	Entrenamiento de habilidades para la vida, basado en el análisis experimental del comportamiento	Estrategia pedagógica / 30 min. / frecuencia variable, generalmente 3 veces por semana	Modificar comportamientos agresivos y disruptivos y fortalecer la concentración en la tarea y la colaboración entre pares en niños
ELOS 2.0	Resultado (n*=1)	Programa de Prevención Universal	Entrenamiento de habilidades para la vida, basado en el análisis experimental del comportamiento	Estrategia pedagógica / 30 min. / frecuencia variable, generalmente 3 veces por semana	Modificar comportamientos agresivos y disruptivos y fortalecer la concentración en la tarea y la colaboración entre pares en niños
PROERD ^§^	Proceso (n*=2) Resultado (n*=7)	Programa de Prevención Universal	Teoría del Aprendizaje Socioemocional, Habilidades Sociales y modelo de influencia social en la educación sobre drogas	Clases / 50 min. / 10 sesiones	Modificar el patrón de consumo de alcohol y otras drogas
*Jogo da Onda Digital*	Resultado (n*=1)	Estrategia de Prevención Universal	Estrategia de psicoeducación y de creencias normativas sobre el consumo de drogas	Juego / 2h / 1 sesión o más	Modificar el patrón de consumo de alcohol y otras drogas
PERAE ^||^	Proceso (n*=1)	Programa de Prevención Universal	Aprendizaje Socioemocional y Reducción de Daños	Clases / 1h / 8 sesiones	Modificar el patrón de consumo de alcohol
Programa M ( *M, de Mulheres* )	Resultado (n*=1)	Programa de Prevención Selectiva	Autoeficacia de Bandura, Cambio de actitudes, Conocimiento Crítico y percepción sobre comportamientos de riesgo	Manual y discusiones en grupo: exposición a videos, campaña de comunicación social / 18 sesiones	Fortalecer la autoeficacia en las relaciones interpersonales de mujeres con impacto en el consumo de alcohol y otras drogas
*Descolado*	Evaluabilidad (n*=1)	Programa de Prevención Universal	Pedagogía de Paulo Freire, Habilidades para la Vida	Clases en escuelas por profesores sobre el consumo de drogas, protagonismo juvenil y Plan de Promoción Personal (PPP) del estudiante	Modificar el patrón de consumo de alcohol y otras drogas
*Educação Sobre Drogas e Habilidades Sociais*	Proceso (n*=1)	Estrategia de Prevención Universal	Psicoeducación	10 sesiones temáticas sobre alcohol y otras drogas y habilidades sociales en clases de Educación Física	Modificar el patrón de consumo de alcohol y otras drogas

*n = Número de estudios; ^†^CRAS = *Centro de Referência de Assistência Social*; ^‡^SUAS = *Sistema Único de Assistência Social*; ^§^PROERD = *Programa Educacional de Resistência às Drogas e à Violência*; ^||^PERAE = *Programa de Estímulo à Saúde e Redução de Riscos Associados ao Uso de Álcool Aplicado ao Ambiente Educacional*

## Discusión

Podría argumentarse que once sigue siendo un número bajo de estrategias o programas preventivos sobre el uso de drogas evaluados en el país, considerando que más de la mitad no cuenta con estudios de eficacia y efectividad que contribuyan a conformar un repertorio más robusto de acciones preventivas basadas en evidencia. No obstante, es importante destacar que ya existen programas sólidamente consolidados en sus evidencias, dirigidos a públicos en diferentes fases del ciclo vital (niños, adolescentes y familias), lo cual constituye un punto de partida fundamental para sustentar la implementación de políticas públicas.

Sin embargo, los escenarios de intervención de las estrategias y programas encontrados siguen siendo limitados, concentrándose principalmente en escuelas públicas o en los *Centros de Referencia de Asistencia Social* (CRAS). En este sentido, sería interesante invertir en el desarrollo o adaptación de programas o sistemas basados en la comunidad, ya que la prevención comunitaria implica el protagonismo y la corresponsabilidad de líderes locales, así como la producción de datos sobre necesidades específicas de cada territorio. Esta es actualmente considerada una de las áreas de actuación más significativas, al incidir sobre los valores locales y proporcionar un sentido de pertenencia a los participantes^(65)^. Del mismo modo, es fundamental invertir más en la prevención ambiental, considerada el ámbito más eficaz de la prevención. Este enfoque actúa de manera amplia, abarcando el escenario social con objetivos como la reducción de la disponibilidad de drogas, el cambio de normas sociales y el control de la publicidad y el marketing, entre otros. Un ejemplo exitoso de esta estrategia fue la prevención del tabaco, iniciada en 1986^(65)^.

En la revisión aquí discutida, se identificaron evaluaciones categorizadas como estrategias y programas preventivos, siendo que la ciencia de la prevención propone la existencia de diferentes niveles de organización de las acciones preventivas, distinguiéndolas entre sí. Las estrategias son planes o enfoques que tienen una base teórica y que han pasado por un proceso de modelado metodológico e intervención orientado a abordar el desenlace objetivo de manera más estructurada. Pueden involucrar la integración de varias acciones coordinadas y orientadas por objetivos a mediano o largo plazo. Las estrategias pueden ser más flexibles en su aplicación, aunque deben ser evaluadas y buscar sustentarse en evidencias^(65)^. Por su parte, los programas preventivos están mucho más sistematizados que las estrategias, ya que deben contar con un modelo lógico y/o una teoría del cambio bien estructurados, con *inputs* claramente definidos (recursos, esfuerzos e insumos necesarios para implementar una actividad), objetivos, *outputs* (resultados inmediatos) y *outcomes* esperados (resultados deseados a mediano y largo plazo), así como indicadores para su evaluación. Además, los programas deben estar, preferentemente, manualizados, es decir, contar con orientaciones estructuradas y objetivas para los implementadores (quienes brindan soporte técnico y supervisión) y para los participantes, con el fin de garantizar la fidelidad de su implementación. Los programas también deben estar sustentados en evidencias. Ambos niveles deben organizar la formación de quienes los implementarán, buscando garantizar la calidad de su implementación y construir propuestas de evaluación de procesos y resultados^(5,65)^.

La clasificación de acciones preventivas según los tipos de prevención es un análisis importante para la toma de decisiones sobre qué programa implementar, de acuerdo con los objetivos dirigidos al nivel de riesgo que presenta la población objetivo. La prevención universal se denomina así porque está dirigida a la población en general, sin ninguna estratificación de factores de riesgo. Se califican como de este tipo los programas que, por ejemplo, cuando se implementan en las escuelas, están dirigidos a todos los alumnos de un aula determinada, sin una selección específica de estudiantes con mayor vulnerabilidad al uso de alcohol y otras drogas (AD). La prevención selectiva, en cambio, está orientada a poblaciones que ya presentan alguna condición de riesgo para el uso problemático de AD, lo que no significa que estas personas ya consuman sustancias psicoactivas, sino que la acción se dirige a quienes tienen mayor riesgo de hacerlo. Por ejemplo, programas dirigidos a hijos de usuarios en recuperación o implementados en escuelas ubicadas en regiones de alta vulnerabilidad, donde el tráfico de drogas está fuertemente presente. Finalmente, la prevención indicada está destinada a personas ya identificadas con alto riesgo de consumo de AD, en quienes ya se ha constatado problemas relacionados con el consumo de sustancias psicoactivas, con el objetivo de minimizar dicho consumo, reducir daños y mejorar la calidad de vida, promoviendo la salud y la reinserción social^(5,65)^. En la revisión predominan las estrategias y programas de tipo prevención universal, siendo necesario reflexionar sobre estas tendencias y cuestionar la posibilidad de diversificar el alcance de las acciones en las políticas públicas brasileñas.

El #*Tamojunto*, en su primera y segunda versión, fue el programa más evaluado, con ciclos de estudios de eficacia, efectividad y fidelidad, discutiendo aspectos que influyeron en la producción o no de los resultados deseados, ya sea cuando obtuvo resultados iatrogénicos que exigieron su readaptación, o cuando, en la segunda versión, alcanzó los resultados esperados al retrasar el inicio del consumo de alcohol, así como efectos secundarios sobre la práctica del *bullying*. Los resultados iatrogénicos desafiaron análisis sobre aspectos del proceso de adaptación cultural y la calidad del proceso de implementación^(23,27)^, y “por vías tortuosas”, contribuyeron al crecimiento de la ciencia de la prevención en Brasil, al exigir que una red de investigadores analizara y aprendiera de los errores, reforzando la necesidad de monitoreo de la implementación y de adecuaciones en la sensibilidad cultural.

Varios estudios del #*Tamojunto*, tanto en su primera como en su segunda versión, dialogaron con las exigencias actuales de estudios avanzados en ciencia de la prevención^(66)^, como los análisis de efectos secundarios, mediación y moderación de este programa. Los estudios de mediación buscaron conocer y analizar los mecanismos del modelo lógico del programa que actuaron efectivamente en la consecución de los resultados obtenidos, siendo estos análisis importantes para redimensionar el propio programa o para la planificación de su implementación a gran escala^(34,36,41,56,61)^. A través de los análisis de moderación, se examinaron elementos contextuales que interfirieron en el resultado, como la calidad de la implementación, las condiciones psicosociales de los estudiantes, entre otros factores^(57)^. Estos análisis más robustos ayudan a comprender cómo funcionan los programas, contribuyendo a la consolidación de las evidencias y a la toma de decisiones^(5,66)^.

El ELOS, en sus dos versiones, presentó resultados de eficacia y efectividad, con cambios en los comportamientos agresivos, disruptivos y en el compromiso con las tareas escolares, mostrando resultados importantes en la disminución de las vulnerabilidades personales de los niños, lo que anticipa la posibilidad de mejora en sus habilidades de vida a futuro. Sus resultados coincidieron con los de otros países y demostraron evidencias de desarrollo de habilidades de vida en los niños y de reducción de vulnerabilidades y comportamientos antisociales, pudiendo ser una excelente acción para utilizar con niños, especialmente varones, como una estrategia de promoción de la salud mental hacia un futuro con jóvenes con mejor autoestima y menores riesgos psicosociales^(54)^. El estudio realizado sobre la moderación de la fidelidad de la implementación en los resultados temporales demostró que el cuidado en la implementación es fundamental para lograr los resultados deseados^(64)^, lo que corrobora los supuestos de la ciencia de la implementación y refuerza, una vez más, la necesidad de monitorear las acciones preventivas^(65)^.

El *Famílias Fortes*, de manera similar, en su estudio de resultados temporales o de efectividad, mostró una mejora en la autoeficacia escolar, lo que ya puede considerarse un efecto secundario importante^(7)^. También se observaron mejoras en las relaciones entre padres e hijos, en las habilidades parentales y en la modificación de estilos parentales negligentes y autoritarios, lo que representa un resultado muy relevante para el fortalecimiento de los vínculos familiares^(47)^, aunque no se observaron resultados directos en el cambio del patrón de consumo de alcohol de los adolescentes. Por lo tanto, el *Famílias Fortes* requiere más investigación para confirmar sus evidencias.

Asimismo, fue importante conocer que el programa preventivo más ampliamente implementado en las escuelas brasileñas, presente en casi todo el territorio nacional, el PROERD, resultó ser inocuo en la mayoría de los desenlaces objetivos o incluso iatrogénico^(15,43,45,55)^. En su evaluación de 2014, aún bajo el modelo DARE, o en la evaluación de 2020, ya bajo el modelo *Keepin’it Real*, no mostró efectos para retrasar la edad del primer consumo de alcohol, no modificó el patrón de consumo de alcohol y otras drogas y fue iatrogénico en relación con el consumo en forma de *binge drinking* entre los adolescentes. Además, también fue inocuo para la práctica o la victimización por *bullying* ^(48,63)^. Esta constatación debe servir como una alerta para los gobiernos municipales, estatales y los gestores de escuelas públicas y privadas sobre la necesidad de solicitar la remodelación de este programa.

Destacamos, con ello, el mérito de los estudios que señalaron resultados negativos, es decir, evaluaciones robustas que muestran efectos inocuos o iatrogénicos de los programas preventivos sobre el uso de drogas, con el fin de indicar la necesidad de cambios en los contenidos o la metodología de los programas y de sugerir modificaciones en su aplicación a nivel local o a gran escala. La literatura muestra la importancia de reflexionar y publicar este tipo de resultados no esperados, para apoyar a los desarrolladores de programas y a los gestores de políticas públicas en la mejora de sus acciones, debiendo considerar las complejidades involucradas en estos resultados negativos y reconocer la multiplicidad de consecuencias intencionadas y no intencionadas al planificar o evaluar intervenciones para prevenir el uso de drogas^(67)^.

En resumen, se constató que existen programas que ya presentan evaluaciones bien elaboradas de proceso, pero que aún no han realizado evaluaciones de resultados, lo que indica la necesidad de promover una nueva serie de estudios para verificar su eficacia y efectividad. En este sentido, las políticas públicas preventivas enfrentan importantes desafíos, como la continuidad de las evaluaciones ya iniciadas para algunos de los programas mencionados, además de fomentar el desarrollo de nuevos programas y sistemas preventivos brasileños.

Sin embargo, uno de los desafíos en el desarrollo de nuevos programas es seguir rigurosamente las etapas del ciclo de investigación en prevención, que requieren tiempo, dedicación de especialistas y una financiación considerable, ya que implican el detalle de las etapas y procesos para producir evidencias, comenzando con el levantamiento de necesidades, el desarrollo de la intervención y la construcción de su modelo lógico y su evaluabilidad, la prueba piloto, la evaluación de la eficacia, la evaluación de la efectividad, la evaluación del proceso de implementación, los estudios de difusión y la adaptación cultural a otros contextos^(31-32,65)^. Otra exigencia importante para el desarrollo de intervenciones preventivas en Brasil es construir procesos participativos y promotores de inclusión, en los cuales “la prevención deje de ser otro mecanismo de control y persecución de personas y amplíe su perspectiva hacia acciones basadas en la integralidad del cuidado y en la dimensión de la ética en su sentido pleno”^(5)^. En esta dirección, deben estar alineados con teorías y metodologías coherentes con los principios que rigen el *Sistema Único de Saúde*, el *Sistema Único de Assistência Social*, la Red de Educación, la Seguridad Ciudadana, la Red de Cultura, entre otros sectores, con el fin de promover la integración de políticas y desarrollar la intersectorialidad^(5,24)^.

La relevancia de esta revisión radica en destacar las características metodológicas y los resultados obtenidos de los estudios evaluativos de estrategias y programas para el uso de alcohol y otras drogas en Brasil, con el objetivo de contribuir a la construcción de un repertorio de acciones preventivas basadas en evidencias, necesario para sustentar la implementación de sistemas preventivos y políticas públicas en este campo. De este modo, Brasil podría comenzar a consolidar el desarrollo de una plataforma nacional para la certificación de estrategias y programas preventivos, fundamentada en estándares científicos claros, que facilitaría el acceso a información sobre acciones preventivas validadas para gestores, profesionales y científicos interesados. Este modelo ya es adoptado en otros países, como el *Blueprints for Healthy Youth Development* ^(68)^ en Estados Unidos, el *Xchange Prevention Registry* ^(69)^ de la *European Union Drugs Agency*, y la *Evidência Viva* ^(70)^, recientemente creada por la *European Union Drugs Agency*, en colaboración con la UNODC y el gobierno brasileño, para compilar programas de América Latina. Además, se destacan algunos sitios desarrollados en países latinoamericanos, como Mi Brújula^(71)^, de la Fundación San Carlos de Maipo de Chile, y la Guía de Programas Preventivos del Consumo de Sustancias Psicoactivas en Colombia^(72)^, de la Corporación Nuevos Rumbos.

Se vislumbra, con ello, la importancia de la inversión pública por parte de un gobierno en el desarrollo de la ciencia de un país, especialmente en el área de la prevención. En el contexto de la producción científica relacionada con los hallazgos que respaldan las políticas públicas, es interesante analizar la inversión de las regiones en el modelo I+D, que implica la claridad de la relación entre investigación y desarrollo del país^(73)^. Esta inversión promueve el avance de un área de producción de conocimiento y apoya la implementación a gran escala de tecnologías sociales basadas en evidencias, trayendo beneficios significativos para la promoción de la salud de la población^(5)^. Los principales programas sobre el uso de alcohol y otras drogas analizados y que actualmente cuentan con indicadores de eficacia y efectividad en nuestro país fueron fruto de estos incentivos públicos.

Este período de inversión en la ciencia de la prevención que el Gobierno Federal realizó entre 2013 y 2018 movilizó el escenario académico, de gobernanza y de implementadores de políticas públicas^(5)^, resultando en la creación de la *Associação Brasileira de Prevenção e Promoção da Saúde* (BRAPEP), que se constituyó para articular investigadores y profesionales, abogando por mejoras en el área, tanto en la investigación y evaluación como en la implementación de políticas públicas, con el objetivo de expandir y cualificar la ciencia preventiva en el país^(74)^.

En este sentido, se destaca el fortalecimiento de la relación entre los formuladores y gestores de políticas públicas y la academia e investigadores, impulsada durante los gobiernos de los presidentes Lula y Dilma, al comprender que la “sistematización del conocimiento y la sensibilidad hacia la cuestión social brasileña no son antagónicas. La ciencia puede y debe ser un instrumento más de transformación de grupos y países”^(5)^. Brasil, de esta manera, poco a poco, comenzó a destacarse por su productividad académica en el campo de la prevención del uso de alcohol y otras drogas, como se analizó, por ejemplo, en una investigación anterior de carácter bibliométrico sobre las redes de colaboración entre países latinoamericanos y de la Unión Europea en producciones científicas en el campo de las AD, entre 2001 y 2011, que demostró un aumento gradual de la colaboración científica entre estos dos bloques de países, sobresaliendo la productividad de Brasil entre los países de América Latina^(73)^.

De la misma forma, el sitio *Evidência Viva*, que compila evidencias de programas de América Latina para establecer recomendaciones, al destacar seis programas con procesos más robustos de evaluación, siendo tres recomendados (*Unplugged*/#*Tamojunto2.0*, *Keepin’ it REAL*, *Good Behavior Game*/ELOS), uno que necesita más evidencias (*Strengthening Families Program/Família Fortes*) y dos no recomendados (*Unplugged/#Tamojunto,* PROERD/D.A.R.E-kiR), utilizó evidencias producidas por estudios aquí analizados. Por lo tanto, de los programas analizados, cinco de ellos tuvieron sus estudios realizados en Brasil^(70)^.

Por último, se pueden señalar algunas limitaciones de este estudio, entre ellas la cuestión del “silencio” en las búsquedas, es decir, cuando algunas publicaciones relacionadas con el objetivo no aparecen en las investigaciones, incluso con el cuidado en la elaboración de la ecuación de búsqueda. Se intentó suplir estas lagunas con la consulta a especialistas en el área. También fue una limitación no haber realizado un análisis sistemático de las referencias de los artículos encontrados, lo que podría haber implicado que algunas publicaciones escaparan de nuestro radar. Otra dificultad de acceso fue la del banco de tesis y disertaciones: no todos los catálogos contienen datos de todos los programas de posgrado, lo que puede implicar que muchos trabajos relacionados con el tema no hayan sido incluidos. Se optó, en algunos casos, por tesis y disertaciones realizadas en modalidad de artículos, y estos fueron encontrados ya publicados, por lo que no se incluyó el trabajo académico, para evitar algunas repeticiones.

## Conclusión

Brasil, con apoyo en inversiones públicas, avanzó para superar la falta de evidencias en el campo de la prevención del uso de alcohol y otras drogas. Ya posee un conjunto de experiencias exitosas en la evaluación de estrategias y programas preventivos, con algunos programas que ya cuentan con evidencias robustas disponibles para las políticas públicas y los interesados en la prevención del uso de alcohol y otras drogas. Las autorías y colaboraciones en las publicaciones analizadas, así como la creación de asociaciones de investigación, evidencian la consolidación gradual de una red de investigadores y especialistas en prevención. Esta red tiene el potencial de contribuir significativamente al desarrollo de políticas públicas basadas en evidencias, además de fortalecer la producción continua de conocimiento mediante la asociación entre la academia y los gobiernos.

La mayoría de los programas evaluados en esta revisión son de carácter universal, dirigidos a adolescentes, desarrollados en escuelas y centrados en varias sustancias. Por lo tanto, es necesario ampliar el alcance de los programas para incluir la prevención basada en la comunidad y la prevención ambiental. De la misma manera, es necesario dirigir esfuerzos hacia nuevos grupos poblacionales, como jóvenes y adultos jóvenes, que constituyen un grupo etario con alto riesgo de involucrarse perjudicialmente en el uso de psicoactivos; y poblaciones indígenas, que han tenido una relación bastante perjudicial al tener contacto con el alcohol y las drogas de la sociedad de consumo occidental. El desarrollo de programas selectivos e indicados también debe considerarse en la planificación futura de proyectos gubernamentales, incluyendo poblaciones de riesgo medio y alto para el consumo de sustancias psicoactivas. Este escenario apunta a la importancia de consolidar una ciencia de la prevención en Brasil que esté sustentada en evidencias, que sea culturalmente sensible y sostenible, contribuyendo a la formulación de políticas públicas más estructuradas e impactantes.
